# Multi-Omics Approaches for Freshness Estimation and Detection of Illicit Conservation Treatments in Sea Bass (*Dicentrarchus Labrax*): Data Fusion Applications

**DOI:** 10.3390/ijms25031509

**Published:** 2024-01-26

**Authors:** Alessandro Benedetto, Elisa Robotti, Masho Hilawie Belay, Arianna Ghignone, Alessia Fabbris, Eleonora Goggi, Simone Cerruti, Marcello Manfredi, Elettra Barberis, Simone Peletto, Alessandra Arillo, Nunzia Giaccio, Maria Angela Masini, Jessica Brandi, Daniela Cecconi, Emilio Marengo, Paola Brizio

**Affiliations:** 1 Istituto Zooprofilattico Sperimentale del Piemonte, Liguria e Valle d’Aosta, Via Bologna 148, 10154 Torino, Italy; alessandro.benedetto@izsto.it (A.B.); simone.peletto@izsto.it (S.P.); alessandra.arillo@izsto.it (A.A.); nunzia.giaccio@izsto.it (N.G.); paola.brizio@izsto.it (P.B.); 2 Department of Sciences and Technological Innovation, University of Piemonte Orientale, Viale Michel 11, 15121 Alessandria, Italy; masho.belay@uniupo.it (M.H.B.); arianna.ghignone@uniupo.it (A.G.); alessia.fabbris@uniupo.it (A.F.); ele.goggi@gmail.com (E.G.); simone.cerruti@uniupo.it (S.C.); elettra.barberis@uniupo.it (E.B.); maria.masini@uniupo.it (M.A.M.); emilio.marengo@uniupo.it (E.M.); 3 Department of Chemistry, Mekelle University, Mekelle P.O. Box 231, Ethiopia; 4 Department of Translational Medicine, University of Piemonte Orientale, Via Solaroli 17, 28100 Novara, Italy; marcello.manfredi@uniupo.it; 5 Department of Biotechnology, University of Verona, Strada le Grazie 15, 37134 Verona, Italy; jessica.brandi@univr.it (J.B.); daniela.cecconi@univr.it (D.C.)

**Keywords:** fish freshness, illicit conservation treatment, Cafodos, data fusion, multivariate statistics, BE-PLS-DA, metabolomics, proteomics, lipidomics, metagenomics

## Abstract

Fish freshness consists of complex endogenous and exogenous processes; therefore, the use of a few parameters to unravel illicit practices could be insufficient. Moreover, the development of strategies for the identification of such practices based on additives known to prevent and/or delay fish spoilage is still limited. The paper deals with the identification of the effect played by a Cafodos solution on the conservation state of sea bass at both short-term (3 h) and long-term (24 h). Controls and treated samples were characterized by a multi-omic approach involving proteomics, lipidomics, metabolomics, and metagenomics. Different parts of the fish samples were studied (muscle, skin, eye, and gills) and sampled through a non-invasive procedure based on EVA strips functionalized by ionic exchange resins. Data fusion methods were then applied to build models able to discriminate between controls and treated samples and identify the possible markers of the applied treatment. The approach was effective in the identification of the effect played by Cafodos that proved to be different in the short- and long-term and complex, involving proteins, lipids, and small molecules to a different extent.

## 1. Introduction

The European Commission (EC) Regulation 853/2004, which establishes specific hygiene rules for foods of animal origin, defines fresh fishery products as “unprocessed fishery products, whether whole or prepared, including products packaged under vacuum or in a modified atmosphere, that have not undergone any treatment to ensure preservation other than chilling”. Over the years, producers and suppliers have made significant progresses in improving the commercial life of fish products [1]. Indeed, considering how easily perishable these products are, the development of technologies aimed at maintaining their economic values and marketability became more and more desirable [2].

On the other hand, processed products are defined as food commodities subjected to treatments able to modify their physico-chemical and organoleptic characteristics (for example canning in oil, smoking, salting, drying, marinating, and freezing).

The degree of freshness of the fish product can be verified by the competent authority, or by the food sector operators responsible for quality control, through a visual (e.g., skin, eye, gills, and consistency) and olfactory examination. For these assessments, discriminant criteria and categories of freshness of fishery products are specified by EC Regulation 2406/96, which can be further integrated by supplementary chemical and microbiological analyses that can be requested during the inspection activity. These are mainly chemical (e.g., lipid oxidation and protein assays, ATP degradation release, biogenic amines quantification, etc.), and microbiological methods carried out according to the available ISO standards (e.g., total viable counts and other evaluation of Pseudomonadaceae, Enterobacteriaceae, etc.). All the cited methods are known to be time-consuming and destructive, often not suitable in the context of field analysis. Therefore, non-destructive, cheap, and rapid techniques, such as near-infrared (NIR) spectroscopy, have also been recently applied [3,4,5,6,7]. However, the use of omics techniques is certainly useful for deeper characterization of fish freshness since it is characterized by complex processes that can be influenced by several endogenous (e.g., spoilage) and exogenous (e.g., undeclared freezing/thawing treatment, adulteration with illicit additives, etc.), factors.

Consequently, while some interesting applications of both omics and spectroscopy have been reported to differentiate the most common frozen/thawed fish frauds [8,9,10,11], novel methods to unveil other illicit field practices based on the misuse of additives that are known to prevent and/or delay fish spoilage are still limited [4] and therefore needed.

In this context, the combination of multiple sources of biological information through the application of omics technologies (e.g., proteomics, metabolomics, lipidomics, and metagenomics) has been evaluated here to develop novel diagnostic tools for the reliable detection of such illicit practices. The application of proper multivariate and data fusion analyses nowadays seems indeed mandatory to reach this aim, being able to improve the classification performance of multivariate models [10,11].

The aim of this study was to determine the effect played by an illicit conservation treatment by simulating the effect of Cafodos on sea bass (*Dicentrarchus labrax*), an economically important species widely cultured worldwide. Cafodos is a mixture of sodium citrate and hydrogen peroxide, used as an approved additive for chilled octopus’ conservation prior to freezing, but also often fraudulently applied to preserve some fish characteristics such as smell, color, or texture [12]. Its use for cosmetic purposes, to mask spontaneous tissue ripening, could pose a potential risk to consumers’ health, like, for example, the progressive accumulation of biogenic amines and histamine, which can cause the so-called scombroid syndrome [13]. Sea bass individuals were then subjected to a short (3 h) and long (24 h)-term treatment with a Cafodos solution and compared to the corresponding controls. Each sea bass sample was characterized by different omics tools (proteomics, lipidomics, metabolomics, and metagenomics) applied to different sampling sites (muscle, skin, gills, and eye). Moreover, an innovative sampling method based on the use of ethyl vinyl acetate (EVA) strips functionalized with different resins [14] was applied to each sampling site (different parts of the fish samples), allowing the analytes, to adsorb directly on the strip surface. The huge amount of data obtained has been tackled by multivariate data fusion approaches [15] to identify the effects of both short- and long-term treatments on all the investigated sampling sites. The merging of different layers of omics data could therefore allow us to better identify and characterize the main endogenous and exogenous biological events that drive and modulate spoilage progression, in order to develop fit-for-purpose diagnostic tools. To the authors’ knowledge, it is the first time that such an exhaustive multi-omic approach has been applied to the study of fish exposed to illicit treatments. 

## 2. Results

### 2.1. Multi-Omic Characterization

#### 2.1.1. Proteomic, Lipidomic and Metabolomic Characterization

The samples were characterized by a multi-omic approach; in particular, the proteomic, lipidomic, and metabolomic characterization of the samples allowed the identification of more than 900 lipids (921 for muscle and 944 for skin), from 340 to 464 metabolites (340 for eye, 380 for gills, 384 for skin, and 464 for muscle), and from 66 to 108 proteins (66 for muscle, 99 for skin, and 108 for gills). Details about the identification of proteins, lipids, and metabolites are provided in Section 4.3, Omics determinations. 

#### 2.1.2. Metagenomics Analysis

**Figure 1:**
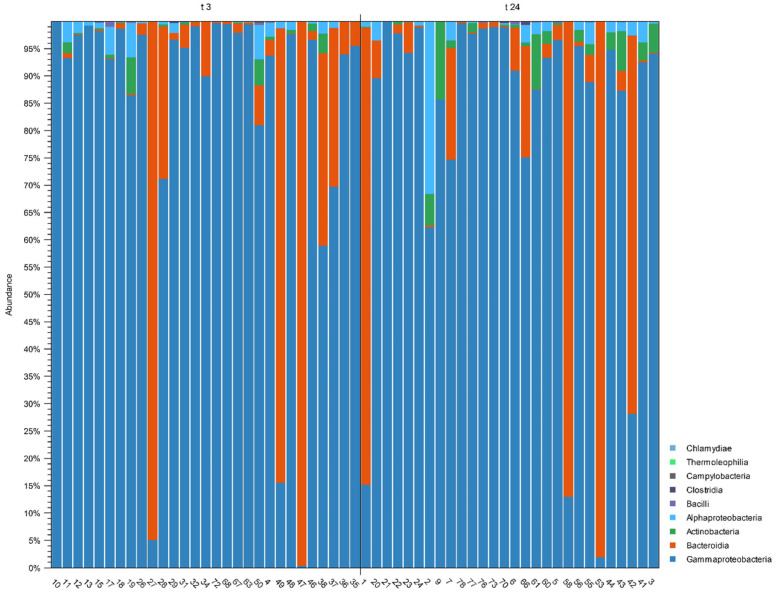
Operational Taxonomic Unit OTU distribution (class level) of all the analyzed samples grouped after 3 h (t3, **left**) and 24 h (t24, **right**) treatment.

As for the metagenomic analyses, a total of 72 samples were analyzed from the skin, gills, and eyes of all the fish samples involved in the study (24 fish × 3 sampling sites = 72 analyzed samples). During the filtering analysis of sequencing data, based on both the quality of the reads (Qscore) and the minimum number of reads per sample, 14 samples were removed from the study being under the minimum fixed thresholds. The total number of paired trimmed reads for the specimens collected from treated animals was 8,698,341 (1,710,396 reads from the gill samples, 4,427,528 reads from the eye samples, and 2,560,417 reads from skin samples). Regarding the samples from untreated control animals, 11,524,798 reads were collected (2,385,590 reads from gill samples, 6,568,250 reads from eye samples, and 2,570,958 reads from skin samples). Sample labels, group subdivisions, and other relevant metadata are reported in Supplementary Material S1. The alignments against sequences available in the reference database (SILVA ver.138) allowed the identification of 428 OTUs, aggregated in 58 families, belonging to nine classes (Figure 1).

At the class level, Gammaproteobacteria, Bacterioda, Actinobacteria, and Alphaproteobacteria were more highly represented, being present in more than 80% of the analyzed samples. Within these classes, the most represented families were Pseudoalteromonadaceae, Moraxellaceae, Comamonadaceae, Shewanellaceae, Flavobacteriaceae, Vibrionaceae, Xanthobacteraceae, Propionibacteriaceae, Pseudomonadaceae, Corynebacteriaceae, and Moritellaceae (see Supplementary Material S1). The clustering of the collected data for each separated sampling site (gill, eye, and skin), grouped at the family level, is detailed in Table 1.

In addition to the reported taxa compositions related to each sampling site, the control and treatment groups with Cafodos (3 h and 24 h, respectively), were then considered. The Alpha and Beta diversity analyses showed no significant differences in microbiome compositions within both single sample and larger sample groups (e.g., eye, skin, and gills). Regarding differential abundance analysis (DAA), the comparisons that resulted in significant differences (FDR adjusted *p* value < 0.05) were recorded only in one specific case: eye and skin samples treated with Cafodos at 3 h vs. 24 h revealed significant alteration in the abundances of Xanthobacteraceae, Comamonadaceae, Oxalobacteraceae, Corynebacteriaceae, Propionibacteriaceae, Rhizobiaceae, Flavobacteriaceae, and Pseudomonadaceae (see Table 2).

Other significant differences in taxa abundances were noted in the comparison of all the specimens (e.g., skin + gills + eye) from the control group (3 h) vs. all the respective specimens from the Cafodos-treated group (after 3 h exposure). The DAA identified a significant increase in Flavobacteriaceae abundance when compared to the untreated control group.

Detailed data of this specific comparison and all other analyses (Alpha diversity, Beta diversity, and multiple DAA comparisons), as described in metagenomics methods (see Section 4.3.4), are detailed in Supplementary Material S1.

### 2.2. Multivariate Statistical Analysis

The results of the multi-omic characterization (proteomic, lipidomic, and metabolomic) were investigated by multivariate statistics to identify the most discriminant molecules separating control and treated samples at both 3 h and 24 h treatments. A data fusion approach was applied to merge all the results from the different platforms together, based on pattern recognition by multi-factor analysis (MFA) and classification by partial least squares discriminant analysis (PLS-DA) coupled to a backward elimination (BE) selection of the most discriminant variables. See Section 4.4, Multivariate data analysis, for more details about these methods.

#### 2.2.1. Multiple Factor Analysis

MFA was applied to each sampling site separately, dividing the variables into blocks depending on the omics characterizations performed: metabolomics, proteomics, and lipidomics. A categorical variable was then added (called “classes”) containing a different label for each class (3 h control, 3 h treated, 24 h control, and 24 h treated). Data were treated as coupled comparisons: measurements from each day (one control and one treated sample) were first centered with respect to the day of measurement, followed by autoscaling (global centering and normalization to unit variance) before MFA. 

**Figure 2:**
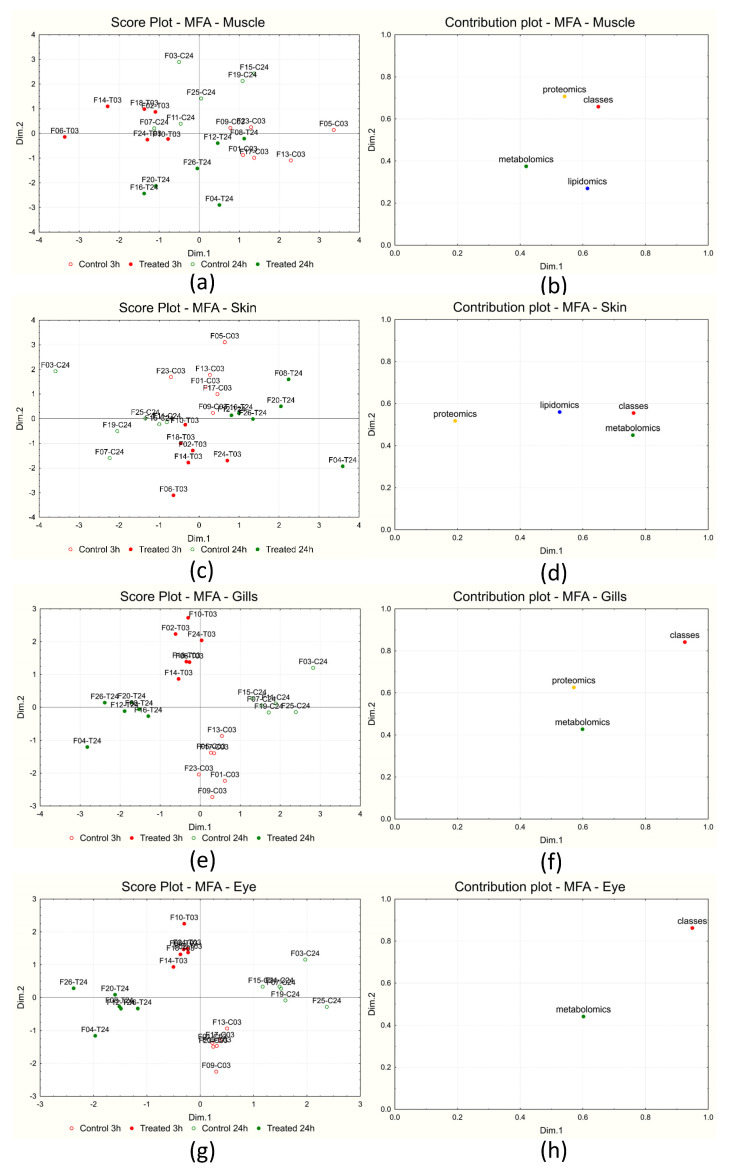
MFA results obtained for the different sampling sites: muscle score plot (**a**) and variable block contribution plot (**b**); skin score plot (**c**) and variable block contribution plot (**d**); gills score plot (**e**) and variable block contribution plot (**f**); eye score plot (**g**) and variable block contribution plot (**h**).

Figure 2 reports the score plots and the block contribution plots obtained for each sampling site (muscle—Figure 2a,b; skin—Figure 2c,d; gills—Figure 2e,f; eye—Figure 2g,h). The score plot reports each sample in the space given by the first two calculated dimensions (Dim.1 and Dim.2), while the block contribution plot reports the contribution of each block of variables on the same first two dimensions. 

Regarding muscle (Figure 2b), the first and second dimensions show the correlation between class belonging and lipidomics and proteomics, respectively; the contribution of metabolomics appears to be evenly distributed between the first and second dimensions. The corresponding score plot (Figure 2a) shows the samples separated into four groups along the two dimensions. The effect of short- and long-term treatments is separated into two contributions along the two dimensions: the short-term effect is accounted for by Dim.1, while the long-term effect is mainly accounted for by the second dimension.

In the case of skin, the contribution plot of the blocks of variables (Figure 2d) shows that class belonging is mainly correlated to metabolomics (Dim1) and proteomics (Dim2). The corresponding score plot (Figure 2c) shows the samples separated into the four groups along the two calculated dimensions: the short-term effect is mainly accounted for by the second dimension (controls at positive scores and treated samples at negative ones), while the long-term effect is explained by the first dimension (controls at negative scores and treated samples at positive ones). 

Regarding the gills, the contribution plot of the variable blocks (Figure 2f) shows that class belonging is correlated to both metabolomics and proteomics on both dimensions. The corresponding score plot (Figure 2e) shows the samples separated into four groups along the two calculated dimensions: as in the case of skin, the second dimension is responsible for the short-term effect, while the first dimension is responsible for the long-term effect.

Finally, in the case of the eye, the contribution plot of the block variables (Figure 2h) only shows the correlation between metabolomics and the grouping variable. Since only one variable block was present in this case, the corresponding score plot (Figure 2g) is almost identical to that obtained for the gills. 

Supplementary Material S2 reports the contribution plots of the original variables on each dimension and for each sampling site separately: the variables are reported on the *x*-axis in order of decreasing contribution, while the corresponding contribution is given on the *y*-axis; only the first 100 variables are represented. In the case of muscle, for both dimensions, the major contribution is given by some proteins, followed by various metabolites and some lipids (the first dimension). In the case of skin, instead, the first dimension shows the major contribution of some proteins, followed by various metabolites, while for the second dimension, proteins appear preponderant. For gills, instead, the major contribution is given by some proteins, followed by various metabolites, for both dimensions.

#### 2.2.2. BE-PLS-DA

The low-level data fusion was applied for each sampling site separately, merging together the different omics characterizations obtained and then performing a BE-PLS-DA on the resulting dataset. 

BE-PLS-DA was applied separately for short-term and long-term treatments, using venetian blind cross-validation with six deletion groups (samples from one day were eliminated simultaneously). As for MFA, data were first centered with respect to the day of measurement and then autoscaled. The results for the four sampling sites are reported in Table 3, comparing the results obtained applying the procedure on each variable block separately (single source results) or on all the blocks contemporarily.

For all the sampling sites, the largest number of discriminant variables came from metabolomics and lipidomics and, to a lesser extent, from proteomics. This is true if both data fusion and single source models are considered, even if data fusion models show in general a lower number of variables, thus proving a higher reliability and a lower risk of chance correlation, considering the number of paired samples included in the study. All the models contain only one latent variable and provide the perfect sample classification in both calibration and cross-validation. In the case of the eye, the same approach was applied, but taking into account only metabolomics results since this sampling site was characterized just by one technique.

**Figure 3:**
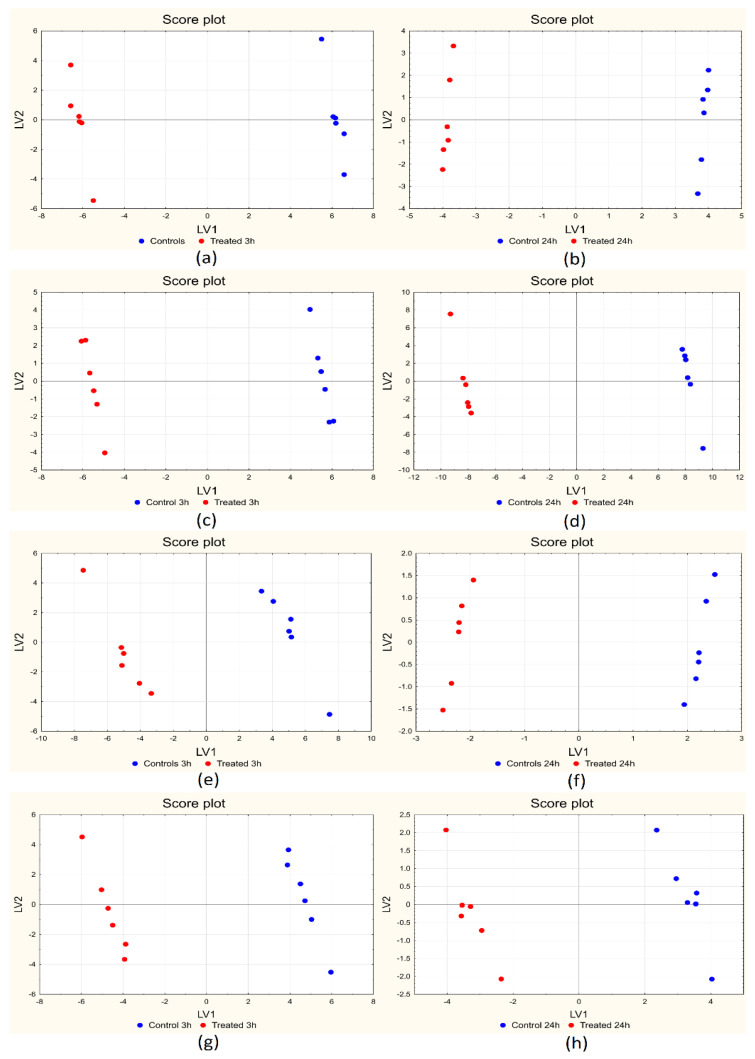
BE-PLS-DA score plots for muscle 3 h (**a**) and 24 h (**b**); skin 3 h (**c**) and 24 h (**d**); gills 3 h (**e**) and 24 h (**f**); eye 3 h (**g**) and 24 h (**h**).

**Figure 4:**
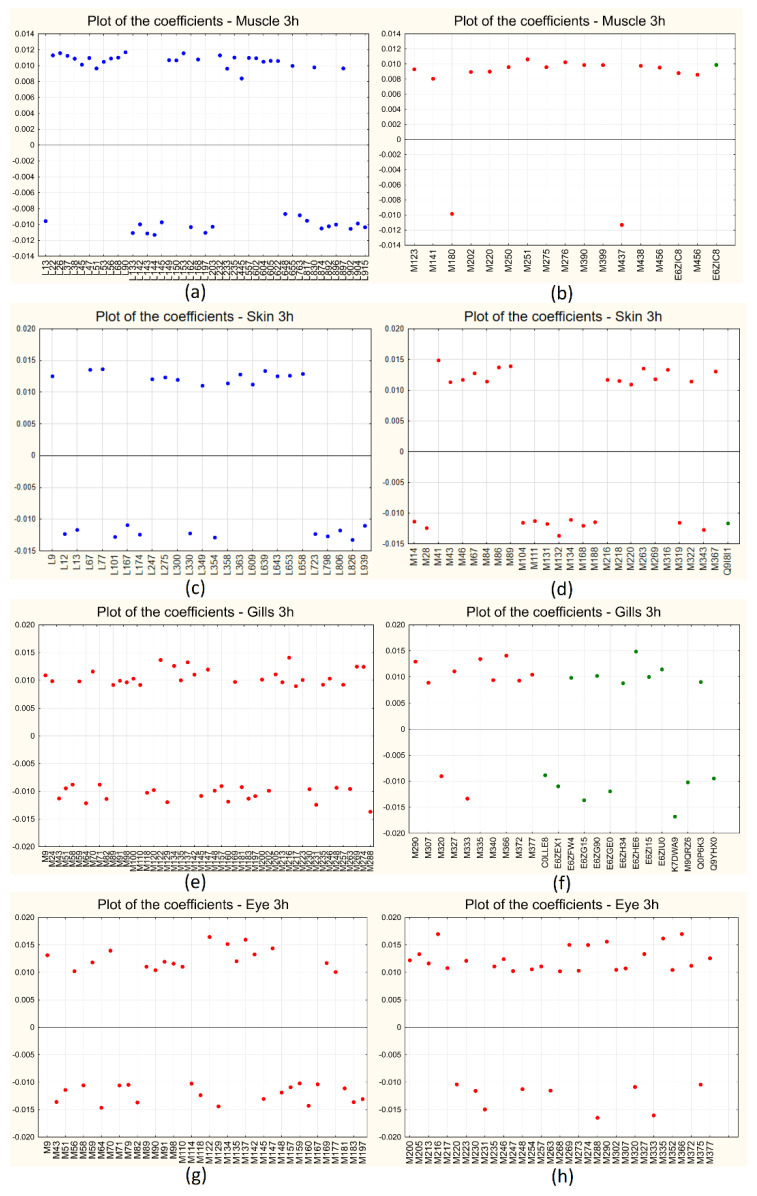
BE-PLS-DA plot of the coefficients for short-term (3 h) treatment: muscle (**a**,**b**), skin (**c**,**d**), gills (**e**,**f**), and eye (**g**,**h**). The variables are indicated on the *x*-axis and the coefficients on the *y*-axis; metabolites are indicated in red, lipids in blue, and proteins in green.

Figure 3a–h shows the score plot of the first two LVs for all the sampling sites at both short- and long-term treatments: for all the cases, the first LV (the only one to be included in the models) is able to clearly separate the two classes of samples, with controls at positive scores and treated samples at negative ones along LV_1_. Figure 4a–h shows the corresponding plots of the coefficients for all the sampling sites for the short-term (3 h) treatment: the variables contained in the models are reported on the *x*-axis (lipids in blue, metabolites in red, and proteins in green), while the coefficients are reported on the *y*-axis. Positive coefficients correspond to molecules that increase after treatment, while negative coefficients correspond to molecules that decrease with treatment. For each sampling site, the plot is split into two panels for a clearer visualization of the results.

**Figure 5:**
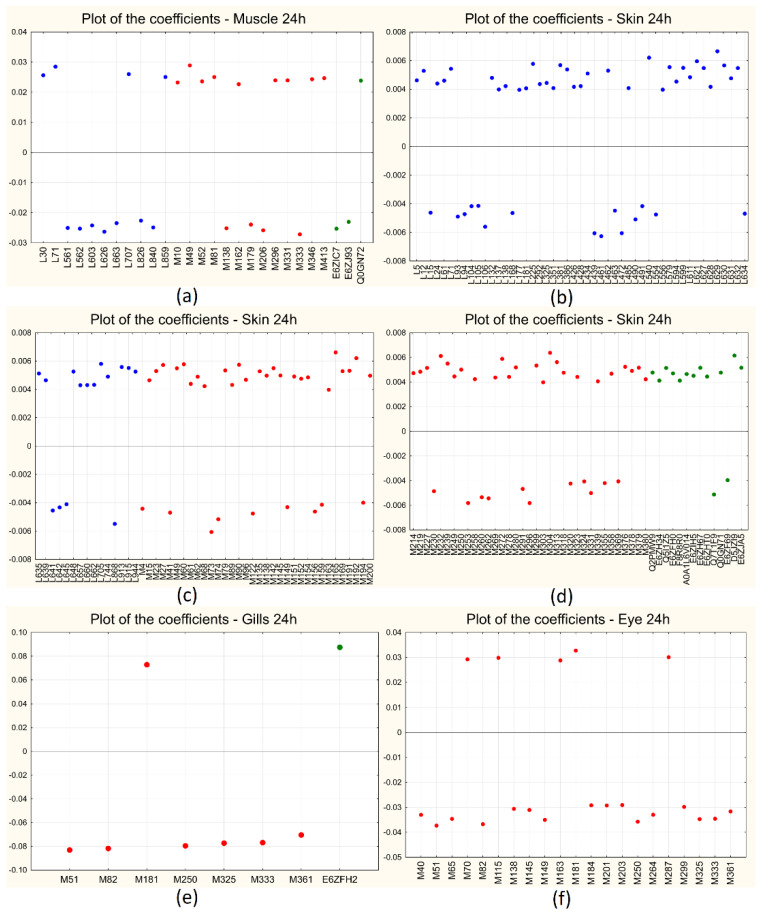
BE-PLS-DA plot of the coefficients for long-term (24 h) treatment: muscle (**a**), skin (**b**–**d**), gills (**e**), and eye (**f**). The variables are indicated on the *x*-axis and the coefficients on the *y*-axis; metabolites are indicated in red, lipids in blue, and proteins in green.

Figure 5a–f shows instead the plots of the coefficients for all the sampling sites for the long-term (24 h) treatment, similar to what has been reported for the short-term treatment: in this case, for each sampling site, the plot is split in three panels only for skin, for a clearer visualization of the results.

An exhaustive list of the discriminant molecules selected by the algorithm for all the comparisons considered is given in Supplementary Material S3, together with the respective coefficients separately for each sampling site and each time of treatment.

### 2.3. Bioinformatics and Data Interpretation

#### Proteomics Ontology Data Analysis

Considering the limited functional annotation of the sea bass genome and proteome, a conversion of the identifiers of the genes coding for the differentially expressed (DE) sea bass proteins into the respective orthologous genes of zebrafish was made, being a more studied and characterized animal model.

Following the conversion into the respective orthologs of *Danio rerio* of all the ENSEMBL identifiers of the genes coding for the DE proteins (gills + muscle + skin), obtained from the comparison between sea bass treated with Cafodos (both after 3 and 24 h) against untreated control sea bass (both at 3 and 24 h), a list of 197 genes was obtained.

**Figure 6:**
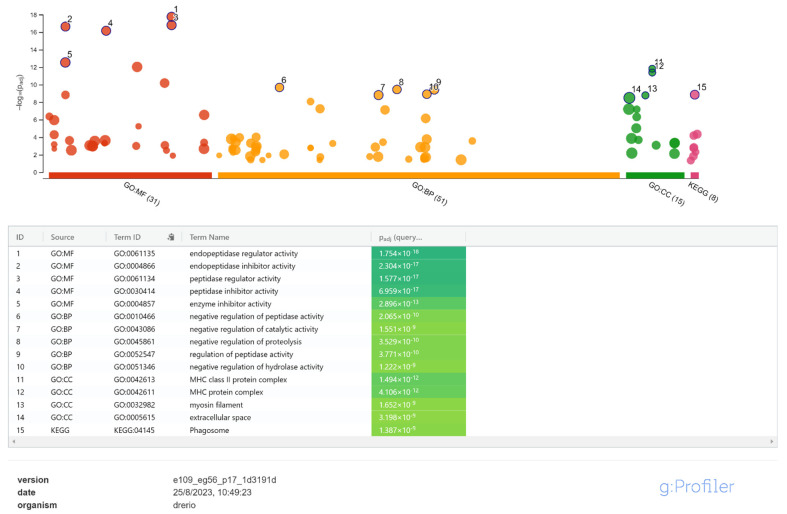
Proteomics results: the most significant GO/KEGG items identified.

The gene ontology enrichment analysis on the gene list resulted in 90 annotated terms for proteins with significant differences in their concentrations between treated and untreated control groups, including 28 MF (molecular function), 41 BP (biological process), 14 CC (cellular component) terms, and 7 KEGG pathways (Kyoto Encyclopedia of Genes and Genomes). The most significant GO/KEGG items are reported in Figure 6. 

These terms are mainly related to the inhibition of proteolytic processes by negative regulation of endopeptidases, which was coherent with the known effects of the Cafodos, able to mask and/or reduce endogenous-driven spoilage dynamics.

## 3. Discussion

### 3.1. Proteomics

From the analysis of the gene ontologies, an overall inhibition effect exerted by Cafodos on the common proteolytic/degradative phenomena that are spontaneously triggered post mortem in the tissues was recorded. The Cafodos effects at the proteomic level on collected sea bass specimens therefore confirm what was already known about the inhibitory effects on proteolysis exerted by peroxides, one of the main components of this prohibited additive [13,16,17,18].

Some of the most discriminant proteins identified by proteomics, their potential correlations with spoilage dynamics, and their role in fish metabolism are then discussed for each sampling site.

#### 3.1.1. Gills

Glutaredoxin (Grx) is a small protein containing an active cysteine pair site [19]. In general, loss of Grx1 leads to redox perturbation of cytosolic proteins and increases the concentration of glutathionylated proteins, disrupting normal redox signaling. Perturbations of redox potential involving both host and microbial oxidative stress pathways are described as strictly related to the spoilage of both meat and seafood products [20]. Recorded changes in Grx levels 3 h after Cafodos exposure (but not after 24 h) seem to confirm the temporary effects of this compound on the residual redox potential.

TANK-binding kinase 1 (TBK1) consists of the serine/threonine protein kinase’s catalytic (S_TKc) domain in the N-terminus, a ubiquitin-like domain (ULD), and two C-terminal coiled-coil domains (CCDs) [21]. In mammals, S_TKc plays an important role in the catalytic activity of TBK1, while ULD determines its kinase activation and CCD regulates its dimerization [22]. To date, TBK1 homologs have been identified in many fish species, such as zebrafish, different carp species (*Cyprinus carpio*, *Carassius auratus*, *Mylopharyngodon piceus*, *Ctenopharyngodon idella*), Atlantic cod, tilapia, Atlantic salmon, and dark sleeper [22]. In our study, a temporary increase in TBK1 levels was recorded in the group treated for 3 h with Cafodos when compared with the corresponding control group. These kinases have been described in other animal species as mainly involved in the regulation of autophagy, induced to overcome the cell damage in the early post mortem stages of muscle ripening, representing therefore one of the mechanisms attempted by cells to maintain homeostasis [23].

Ribosomes are essential for life, generating all proteins required for cell growth and maintenance. In addition, many ribosomal proteins (RPs) are believed to have important functions in various other cellular processes, the so-called extra ribosomal functions [24]. A recent study [25] has shown the presence of 40S and 60S RPs in sea bass and mentions the fact that the ribosomal pathway might be closely associated with protein degradation and changes in muscle quality [25]. In our proteomics dataset, a significant differential abundance of RPs was identified (decrease of both 40S and 60S RPs 3 h after Cafodos exposure and an increase in their mean concentrations 24 h after treatment); however, clear correlation trends were not identified.

Cystatins represent a large superfamily of proteins involved in the competitive reversible inhibition of C1 cysteine proteases. Cystatins are known to inhibit the activity of target proteases by indirectly obstructing the catalytic centers, to prevent the docking and cleavage of substrates [25]. In a study on Atlantic cod [26], the presence of cystatin B in the epidermis might serve to inhibit the cysteine proteases from invading pathogens. The higher levels of these proteins in fish treated with Cafodos, 3 h after exposure, could contribute to the alleged inhibition of spontaneous proteolysis.

Calreticulin (CRT) is a key molecular chaperone and regulator of Ca^2+^ homeostasis in the endoplasmic reticulum (ER), also implicated in a variety of physiological/pathological processes outside the ER [27]. For example, its overexpression has been described in the study of H_2_O_2_-induced apoptosis in melanocyte cell lines [28], but, conversely, a post mortem reduction of CRT levels 3 h after exposure to Cafodos (that contain high concentrations of H_2_O_2_) was reported in our animal model.

Osmoregulation in fish is mainly performed by sodium and chloride transport that occurs in gills, kidneys, and intestines through ionocytes, also known in gills as mitochondrion-rich cells (MRC) [29]. These cells harbor different ion transporters and channels whose localization and expression have been studied in the sea bass by many researchers [30,31,32]. In the sea bass, as in other teleosts, excretion of salt in hypertonic media is mediated by a co-transporter, NKCC1, located in the basolateral membrane of MRCs, coupled to an apical Cl^−^ channel (CFTR) that transports Cl^−^ outside the cell [29]. These regulations [29] are fundamental for the migrations of *D. labrax* between environments at different salinities. In the experimental conditions applied in our study, we identified a significant difference in NKCC1 levels between Cafodos-treated and untreated (control) groups, limited to the early exposure phase (3 h), suggesting potential modulation of these illicit additives on such ion transponders.

Inositol monophosphatase 1 (IMPA1.1) and myo-inositol phosphate synthase (MIPS) are the two enzymes comprising the myo-inositol biosynthesis (MIB) pathway that plays a key physiological role in teleost osmoregulation because they convert glucose-6-phosphate to the compatible organic osmolyte myo-inositol, which protects cells from salinity-induced damage [33]. In the packaging/conservation procedures of meat products under anoxic conditions, significant alterations in myo-inositol and glycerol levels have been described [34], influencing, therefore, microbial proliferation in fatty meat products like fish. Also in our context, the temporarily decreased levels of IMPA1.1 and MIPS in the Cafodos-treated group (3 h after exposure) could potentially have some implications for delayed microbial proliferation, often related to early tissue deterioration.

The Na^+^/K^+^-ATPase (NKA) is considered the main pump involved in active ion transport; it is the most important enzyme involved in osmoregulation in fish and provides the driving force for ion transport in the osmoregulatory organs in seawater and fresh water [29]. Higher levels of NKA were recorded in the Cafodos-exposed group after 24 h when compared to the respective control group, but its specific contribution towards or against tissue spoilage will need further studies. 

#### 3.1.2. Skin

The heat shock protein 90 (HSP90) plays an essential role in the modulation of phenotypic plasticity in vertebrate development; however, the roles of HSP90 in the modulation of cold tolerance capacity in fish are still unclear [35]. A study by Han et al. reports that the transient inhibition of the embryonic HSP90 function by a chemical inhibitor or by low conductivity stress promoted the variation in cold tolerance capacity in adult zebrafish [35]. The exposure to Cafodos induced a quick and temporary increase in HSP90 after 3 h, therefore confirming the main effects of the hydrogen peroxide contained in this illicit additive.

The skeletal myosin light chain 2 (MLC2) is a structural molecule of the muscle that exists in two main isoforms, which are known to mark hyperplasia and hypertrophy in gilthead sea bream [36]. Some studies on meat-producing animals identify MLC2, troponin T, and some heat shock proteins as the main targets of phosphorylation changes related to early meat aging caused by slaughter procedures [37], suggesting therefore potential implications also in seafood spoilage. Our findings (higher levels of MLC2 in fish after 24 h of Cafodos treatment vs. the respective control group) confirmed an overall stabilization effect of peroxides and citrate mixtures on proteins.

Apolipoproteins (Apos) are the protein components of plasma lipoproteins and play important roles in the transportation of lipids in vertebrates. It has been demonstrated that, in teleosts, several Apos display antimicrobial activity, playing crucial roles in innate immunity [38]. Instead of carbohydrates, fish always exploit lipids as the main energy source, suggesting that lipid metabolism and lipoprotein physiology may be more important for homeostasis in fish than in humans [38]. In our study, an early decrease in Apos concentrations was recorded in the 3 h Cafodos-treated seabass, showing in this case the unexpected and controversial effects of this prohibited additive.

#### 3.1.3. Muscle

The muscle-specific protein myomesin is a major candidate for the role of M-line bridges to regulate the packing of thick filaments and to uniformly distribute the tension over the myosin filament lattice in the activated sarcomere. Three myomesins (myomesin-1, -2, and -3) have been identified in the skeletal and cardiac muscles of vertebrates. Myomesin-1 is ubiquitously expressed in all types of striated muscles [39]. The myosin heavy chain is a commonly identified protein in studies on muscle changes during storage [40,41]. Another study revealed that the myosin heavy chain is broken down into proteins with smaller molecular weights (myosin heavy chain fragments) during storage [42]. The higher concentrations of myomesin and myosin heavy chain found in Cafodos-treated fish (for both 3 and 24 h sampling time points) suggest a potential delay in spontaneous degradation of main muscular proteins.

Annexins (also commonly called lipocortins) are a family of structurally related proteins whose common properties are the binding of both phospholipids and cellular membranes in a calcium-dependent manner [43]. Recent works on meat maturation revealed how annexins could contribute to limiting the degradation of myofibrillar proteins, hindering the activation of the calpain and caspase proteolytic systems [44], suggesting therefore potential implications also for the spoilage of fish muscle. Interestingly, after a preliminary and unexpected decrease in annexins in the 3 h Cafodos-treated fish, an increase in their concentrations was recorded 24 h after exposure, thus remaining more in line with the expected protective effects of this protein family towards other structural muscular proteins.

### 3.2. Lipidomics

The main role of lipids in these organisms is the storage and provision of energy in the form of adenosine triphosphate (ATP) through the β-oxidation of fatty acids [45]. Fish lipids are well known to be rich in long-chain n-3 polyunsaturated fatty acids (LC n-3 PUFA), especially eicosapentaenoic acid (EPA or 20:5n-3) and docosahexaenoic acid (DHA or 22:6n-3). These fatty acids play a vital role in human nutrition, disease prevention, and health promotion [46].

**Figure 7:**
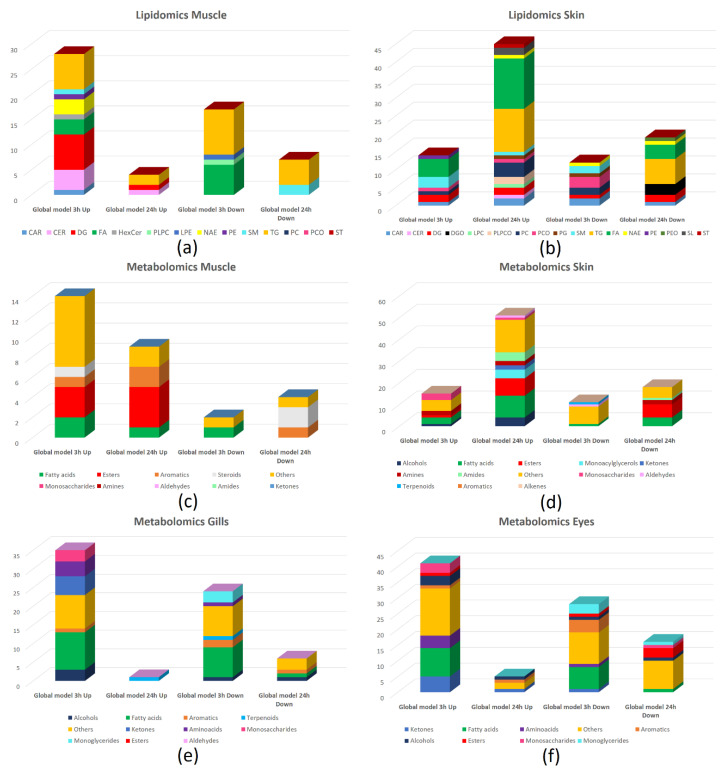
Lipidomics and metabolomics results. Discriminating lipids (**a**,**b**) and metabolites (**c**–**f**) identified by the global (data fusion) BE-PLS-DA models built: lipids for muscle (**a**) and skin (**b**) after 3 h and 24 h treatment; metabolites for muscle (**c**), skin (**d**), gills (**e**), and eye (**f**) after 3 h and 24 h treatment. Bar diagrams represent the discriminating lipids or metabolites separated into different classes; for each model, two bar diagrams are present showing the number of lipids or metabolites of each class decreased (down) and increased (up) after the Cafodos treatment, separately.

The results obtained from lipidomics after 3 h and 24 h of Cafodos treatment are reported in Figure 7a,b: for the data fusion BE-PLS-DA models built for muscle and skin after 3 h and 24 h treatment, bar diagrams represent the discriminating lipids separated in different classes; for each model, two bar diagrams are present showing the number of lipids of each class decreased (down) and increased (up) after the Cafodos treatment, separately. The results show a deep alteration of the lipid metabolism with both the increase or decrease in different classes of lipids and different effects for short- and long-term treatment. 

In the study by Alasalvar et al. [46] on cultured and wild sea bass, the major fatty acids identified in both fish types were 16:0 (palmitic), 18:0 (stearic), 18:1n-9 (oleic), 20:5n-3 (eicosapentaenoic acid, EPA), and 22:6n-3 (docosahexaenoic acid, DHA). In our study, EPA, DHA, palmitic, and oleic acids show a decrease after 3 h treatment (DHA decreases also after 24 h), while stearic acid shows an increase after 3 h treatment. These effects show the alteration of the fatty acid metabolism played by the Cafodos treatment. 

However, considering known variability in feed intake, diet composition, and other seasonal and geographical variances in sea bass farming [4,47,48], the specificity of perturbations induced by Cafodos on recorded fatty acid profiles will need to be checked on supplementary fish specimens.

### 3.3. Metabolomics

Figure 7c–f reports the results for metabolomics for muscle, skin, gills, and eye, as already described for lipidomics. In general, for muscle and skin, a higher number of molecules increasing, rather than decreasing, after treatment is recorded for both short- and long-term effects. Skin, gills, and eyes show the most relevant effects with a higher number of detected discriminating molecules, while muscle shows the lowest effect, in fact, it is not directly in contact with the Cafodos solution. 

The alteration of different classes of metabolites is probably deeply related to the differences observed for lipids. The high contents of unsaturated fatty acids in sea bass are indeed, together with its characteristic high protein levels, among the main causes of the short shelf-life: the high rates of oxidation and degradation of these compounds consequently bring higher levels of aldehydes, ketones, and amines, favored by the residual catalytic activities of endogenous enzymes and by the microbial proliferation. Alterations to one or more of these components can therefore bring a corresponding alteration of the others. From a future perspective, further studies will be needed for the structural characterization and the straightforward identification of small metabolites, to deepen the biological features of the most discriminant small biomarkers identified by the calculated multivariate models, and to understand their relationship with lipids and proteins selected by BE-PLS-DA. This approach has indeed been recently and successfully applied to metabolomics data for the differentiation of fresh and frozen/thawed sea bass fillets [10].

### 3.4. Metagenomics Analysis

The 16S rRNA sequencing analysis allowed us to assess the microbiome composition on the skin, eye, and gills of sea bass under normal storage conditions and after illicit treatment with Cafodos (3 h and 24 h treatment). Collected data showed that the sea bass microbiome compositions after 24 h of ice storage, in terms of prevalent OTUs identified, are in line with what has already been reported in other studies [49], also when considering both other commercially relevant fish species, and other allowed storage conditions, like modified atmosphere packaging [50]. Potential perturbations induced by Cafodos in the microbiome environment are present and reported (see Supplementary Material S1), but basal microbial heterogeneity of whole tested sampling sites did not allow the clear and significant identification of specific OTUs that could be related to Cafodos exposure (i.e., no relevant alterations recorded in terms of both Alpha and Beta diversity). Only in the eye and skin of fish after 24 h of Cafodos exposure, some significant differences in OTU abundances were recorded by DAA analysis of 16S rRNA metabarcoding data. In this case, an interesting shift in microbiome relative composition was recorded, characterized by a significant reduction (FDR *p*-value < 0.05) of Xanthobacteraceae, Comamonadaceae, Oxalobacteraceae, Corynebacteriaceae, Propionibacteriaceae, and Rhizobiaceae (see Table 2). In the context of food safety aspects related to microbial growth, consequent and potential modulation effects of Cafodos could therefore be assumed also on pathogens [51]; however, these findings need to be confirmed by further experiments, in order to clarify the reported results.

## 4. Materials and Methods

### 4.1. Study Design and Illicit Treatment Application

The samples were collected during an animal trial on farmed European sea bass (*Dicentrarchus labrax*) previously reported [16].

All animals belonged to a single farm and were delivered to the laboratory in less than 24 h from capture, through a local supplier chain, thus assuring their homogeneity for production cycle and commercial size. The individuals were arranged into four groups: (i) treated with a Cafodos solution and stored in ice at 4 °C for 3 h and (ii) 24 h; (iii) controls stored in ice at 4 °C for 3 h and (iv) 24 h. Six individuals of each group were considered, for a total of 24 fish individuals, and the experimentation was carried out over 12 days: one treated sample and the corresponding control were treated per day as paired comparisons. This procedure allowed the evaluation of the biological variability and, in the meantime, the timing evaluation of the applied effect, soon after treatment, while keeping the measurement number limited. 

The Cafodos solution contained hydrogen peroxide (8 g/L) and citric acid (2.5%, *w*/*v*); each treated sample was immersed in the solution for 60 s, then placed into a food bag and stored on ice at 4 °C for 3 h or 24 h. Controls were treated in a similar way substituting the solution with ultrapure water. A ratio equal to 1:1 (*w*/*v*) was used between the fish weight and the solution volume. The treatment was followed by washing for 2 min with deionized water and draining. 

### 4.2. Sample Collection and Pre-Treatment

**Figure 8:**
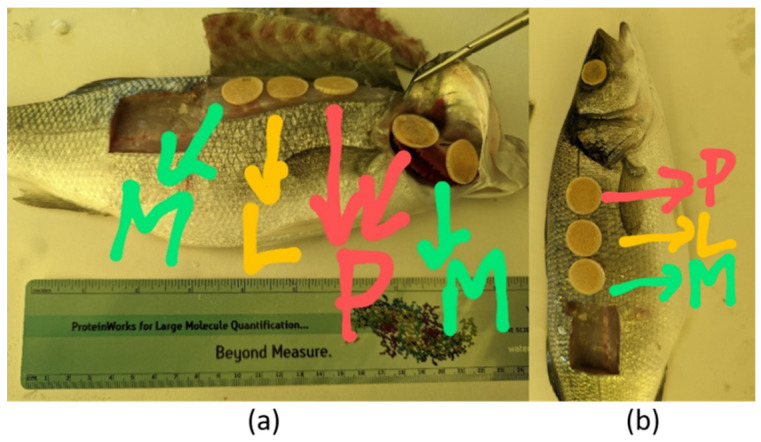
Applied sampling procedure: application of the strips for muscle and gills sampling (**a**) and for skin and eye sampling (**b**). M indicates metabolomics, P indicates proteomics, and L indicates lipidomics. In the case of the eye, no letter is indicated since only one strip is adopted and used for the metabolomic characterization.

Each of the 24 fish samples was characterized by different omics investigations (Figure 8): one side of the fish was exploited for the multi-omic characterizations, while the other side was used for the metagenomic sampling and for spectroscopic characterizations (previously reported [16]). Different sites of each fish were sampled: muscle and skin (complete multi-omic characterization), gills (metabolomics, proteomics, metagenomics), and eye (metabolomics, metagenomics).

To reduce destructive and/or invasive sampling procedures, in the case of proteomics, lipidomics, and metabolomics, ethylene vinyl acetate strips (EVA) were used [14]. The strips were prepared according to [14]. Briefly, a mixture (BIO-RAD MG AG^®^ 501-X8, 20–50 mesh) containing both anion (CASNr 60177-39-1) and cation (CASNr 69011-20-7) exchange resins was used. The resin mixture was ground in a mortar to 30–50 μm thickness per particle. An amount of 1.5 mL Eppendorf Safe-Lock tubes were filled up to 0.5 mL of the resin mixture and 1.0 mL was added of a mixture of EVA and cyclohexane, previously prepared to mix 4 g of polymer and 10 mL of solvent by heating. After shaking to evenly spread the resin particles, the mixture was poured into round molds of about 1 cm diameter. After drying under a fume hood, the strips were removed with tweezers and stored in plastic bags. 

The strips were applied to one side of each fish (Figure 8) and each strip was used for a single omic characterization: for muscle and skin, three strips were placed in a region between the dorsal fin and the lateral line, while for gills and eyes, 2 and 1 strips were used, respectively. To improve adhesion and capture of the analytes, the strips were wetted with ultrapure water for 10 s; once placed, they were held in place for 10 min before being removed and placed in Eppendorf tubes at −80 °C until analysis. 

For microbial DNA sequencing, all specimens were collected on the fish side and not subjected to EVA strip application by sterile eNat swabs (Copan Diagnostics, Murrieta, CA, USA). 

#### 4.2.1. Proteomics

The proteins were eluted from the EVA strips with 500 µL of 1.0 M ammonium acetate in a test tube for 30 min. Then, the strips were removed and the proteins in the solution were denatured with TFE at 60 °C, reduced with dithiothreitol (DTT) 200.0 mM, alkylated with iodoacetamide (IAM) 200.0 mM, and digested with trypsin overnight. Salts were removed from peptide digests using the 96-well plates functionalized with 25 mg/well Discovery^®^ DSC-18 solid-phase extraction (SPE) (Sigma-Aldrich Inc., St. Louis, MO, USA).

#### 4.2.2. Lipidomics

Adsorbed lipids were eluted using cold methanol (225 μL) and cold MTBE (750 μL). After vortexing, 100 μL of water were added and the samples were vortexed for 10 s, then centrifuged for 2 min at 14,000 rpm at 4 °C. Finally, the supernatant was collected and evaporated using a SpeedVac. The dried sample was reconstituted using 50 μL of a 9:1 (*v*/*v*) MeOH:toluene solution containing 12-[[(cyclohexylamino)carbonyl]amino]-dodecanoic acid (CUDA) at a concentration of 12.5 ng/mL as internal standard.

#### 4.2.3. Metabolomics

Small molecules captured by the EVA film were eluted using ethanol (1 mL) for 30 min under sonication. Then the strips were removed, and the metabolites were subjected to derivatization by adding 20 μL of methoxamine hydrochloride in pyridine (20 mg/mL) and 50 μL of N,O-Bis(trimethylsilyl) trifluoroacetamide (BSTFA). The samples were incubated at 80 °C for 20 min and then centrifuged for 15 min at 14,500× *g*. Standard solutions of tridecanoic acid (1 ppm) and hexadecane (0.1 ppm) were added as internal standards before derivatization and GCxGC-MS analysis, respectively.

#### 4.2.4. Metagenomics 

For microbial DNA sequencing analysis, swabs were placed in collection tubes loaded with guanidine-thiocyanate-based medium effective for the optimal stabilization of viral and bacterial nucleic acids. All procedures were carried out to guarantee sampling sterility. DNA extraction was then performed using the QIAamp PowerFecal Pro DNA Kit (Qiagen, Hilden, Germany) following the manufacturer’s instructions. 

### 4.3. Omics Determinations

#### 4.3.1. Proteomics

The extracted proteins were analyzed with a µLC-HRMS technique. The analyses were performed using an Eksigent Technologies micro-LC system (Dublin, CA, USA) interfaced with a 5600 + TripleTOF instrument (AB Sciex, Concord, ON, Canada). 

A reversed-phase C18 column (Halo C18, 0.5 × 100 mm, 2.7 µm), thermostated at 40 °C, was used for chromatographic separation. The elution was carried out with a mobile phase A consisting of 0.1% (*v*/*v*) formic acid in water and a mobile phase B consisting of 0.1% (*v*/*v*) formic acid in acetonitrile. The flow rate used was 15.0 µL/min with a mobile phase gradient from 2% B to 40% B in 30 min. The injection volume of each sample was 4.0 μL.

Samples used to generate the SWATH spectral library were subjected to data-dependent acquisition (DDA) and then cyclically to data-independent analysis (DIA), using a 25 Da window. MS data were acquired using Analyst TF v.1.7 software (AB Sciex). PeakView v.1.2.0.3 and Protein Pilot v.4.2 (AB Sciex) software were used to generate the peak list. A search for protein identification was performed in the MS files using Protein Pilot. Quantification was performed with PeakView and MarkerView v.1.2 (AB Sciex) by integrating the extracted ion chromatogram of all ions unique to a given peptide. The six peptides per protein with the highest MS1 intensity and six transitions per peptide were extracted from the SWATH files. Peptides with FDR less than 1% were exported for univariate and multivariate statistical analyses.

Enrichment analysis on the lists of proteins characterized by differential abundances was then performed to identify the main Gene Ontology (GO) biological process terms (BP), molecular function terms (MF), cellular component terms (CC), and KEGG pathways using the gProfiler web tool [52], with the reported settings: ordered query ranked by log2 fold change expression values, considering only annotated proteins, with gSCS threshold < 0.05. For the analysis, a preliminary conversion of sea bass protein IDs to relative zebrafish (*Danio rerio*) orthologues was made using the gProfiler Gene ID conversion tool.

#### 4.3.2. Lipidomics

A Vanquish UHPLC system (Thermo Scientific, Rodano, Italy) coupled with an Orbitrap Q-Exactive Plus (Thermo Scientific, Rodano, Italy) was used for lipid analysis. For lipid separation, a reversed-phase column (Hypersil Gold^TM^ 150 × 2.1 mm, particle size 1.9 μm) was used; the column was maintained at 45 °C at a flow rate of 0.260 mL/min. The injection volume was 3 μL.

For the positive ESI mode, mobile phase A was acetonitrile/water 60:40 (*v*/*v*) with ammonium formate (10 mmol), while mobile phase B was isopropanol/acetonitrile 90:10 (*v*/*v*). Both of them were modified with ammonium formate (10 mmol/L) and 0.1% formic acid. For the negative ESI mode, the organic solvents of both mobile phases were the same as those used for the positive mode, except for the addition of ammonium acetate (10 mmol/L) as an organic modifier. 

The applied gradient was the following: from 70%A and 30%B to 57%A and 43%B in 2 min, to 45%A and 55%B at 2.1 min; to 35%A and 65%B at 12 min; 15%A and 85%B at 18 min; up to 100%B at 20 min and maintain for 5 min; equilibration to 70%A and 30%B in 5 min.

Analysis by mass spectrometry was performed in both positive and negative ion modes. The source voltage was kept at 3.5 kV in the positive ion mode and 2.8 kV in the negative ion mode. All other interface settings were identical for the two modes: capillary temperature 320 °C; sheath gas flow 40 arb; auxiliary gas flow 3 arb; S-lens 50 rf. 

Data were collected in data-dependent mode (ddMS2) top 10. Full scan MS spectra (*m*/*z* mass range 80 to 1200) were acquired with resolution R = 70,000 and target AGC 1 × 106. MS/MS fragmentation was performed using high-energy C-trap dissociation (HCD) with resolution R = 17,500 and target AGC 1 × 105. The step-normalized collision energy (NCE) was set to 15, 30, and 45, respectively. 

For accurate mass-based analysis, the Lockmass system and regular calibrations between runs were used. An exclusion list for background ions was generated by analyzing the same procedural blank sample for both positive and negative ESI modes. Internal standards covering a range of analyte classes at appropriate levels (Avanti SPLASH Lipidomix) and an internal standard (CUDA) added prior to LC-MS analysis were used. Lipid species were identified and quantified using MS-DIAL and Lipid Search software 4.24 version (Yokohama City, Kanagawa, Japan) but also using custom libraries.

#### 4.3.3. Metabolomics

A LECO Pegasus 4D GCxGC/TOFMS instrument (Leco Corp., St. Josef, MI, USA) equipped with a LECO dual-stage, quad-jet thermal modulator was used to perform the analyses. The GCxGC was an Agilent 7890 gas chromatograph (Agilent Technologies, Palo Alto, CA, USA) equipped with a split/splitless injector. 

The first column was a 30 m Rxi-5Sil MS capillary column (Restek Corp., Bellefonte, PA, USA) with an inner diameter of 0.25 mm and a stationary phase film thickness of 0.25 μm. The second size chromatography column was a 2 m Rxi - 17Sil MS (Restek Corp., Bellefonte, PA, USA) with a diameter of 0.25 mm and a film thickness of 0.25 μm. 

Helium was used as the carrier gas at a flow rate of 1.4 mL/min. An amount of 1 μL of sample was injected in splitless mode with the following schedule: initial temperature 40 °C, 5 min isothermal, 8 °C/min up to 300 °C, 20 min isothermal. The secondary column was kept at +5 °C relative to the GC oven temperature of the first column. 

Mass parameters were electron impact ionization source temperature (EI, 70 eV) at 250 °C; scan range from 40 to 630 *m*/*z*, with extraction frequency of 32 kHz. The acquisition rate was 200 spectra/s, and the modulation period was kept at 4 s for the entire analysis. The modulator temperature offset was set at +15 °C relative to the secondary oven temperature, and the transfer line was set at 280 °C.

Chromatograms were acquired in TIC (total ion current) mode. Mass spectrum assignment was performed by comparing the mass spectra obtained with the NIST MS Search 2.3 library, implemented with the MoNa Fiehns libraries. For data processing, the calculation of library search similarity score was set >700 (identification reliability index) in order to select only library results with higher reliability.

#### 4.3.4. Metagenomics

Nucleic acids quantity and quality were determined by spectrophotometric (VivaSpec LS, Sartorius Göttingen, Germany) and fluorimetric (Qubit dsDNA HS, Thermo Fisher, Carlsbad, CA, USA) measurements.

The 16 S Metagenomic Sequencing Library Preparation protocol (Illumina, San Diego, CA, USA) was chosen for the metagenomic analysis, using primers 341FB and 806RB [53,54] targeted to hypervariable V3–V4 regions of the 16S rRNA.

The PCR was performed in a final volume of 25 μL using 12.5 μL PCRBIO HS VeriFITM Mix master mix (PCRBiosystems, Wayne, PA, USA), 1.25 μL of each primer 10 μM, 7.5 μL H_2_O, 2.5 μL DNA (5 ng/μL), with the following thermal profile: 95 °C for 1 min; 35 cycles at 95 °C for 15 s; 55 °C for 15 s; 72 °C for 30 s; 72 °C for 2 min. Molecular biology grade water was included as a negative control.

PCRs were visualized on 2% (*w*/*v*) agarose gel to check expected amplicon sizes. The amplified DNA was then purified by Agencourt AMPure XP (Beckman Coulter, Brea, CA, USA) and submitted to index PCR. Each 50 μL reaction was prepared by adding 5 μL of DNA to 5 μL of each index primer mix (IDT^®^ for Illumina^®^ DNA/RNA UD Indexes set A, Illumina, San Diego, CA, USA), 25 μL PCRBIO HS VeriFITM Mix (PCRBiosystems, USA), and 10 μL of ultrapure water, with the following thermal profile: 95 °C for 1 min; 12 cycles at 95 °C for 15 s; 55 °C for 15 s; 72 °C for 30 s; 72 °C for 2 min.

The PCR products were purified again by magnetic beads clean-up (Beckman Coulter, USA) and tested on a Bioanalyzer 2100 (Agilent, Santa Clara, CA, USA) by High-sensitivity DNA kit chip to verify the library size. The amplified fragments were also quantified with the Qubit DNA HS kit on a Qubit 2.0 fluoroimeter (ThermoFisher, Waltham, MA, USA) for library normalization (final concentration of 4 nM). Library concentration was further checked by qPCR using the Invitrogen Collibri Library Quantification Kit (ThermoFisher). The pooled library was then sequenced on an Illumina MiSeq platform (MiSeq Reagent Kit v3-600-cycle) by paired-end 2 × 300 bp sequencing.

After demultiplexing, raw fastq data were analyzed with the “Data QC and OTU Clustering” and “Estimate Alpha and Beta Diversity” workflows of the Microbial Genomics Module in the CLC Genomic Workbench v. 23 (Qiagen). The paired-end reads were joined and trimmed for low-quality score (Qscore < 0.05), nucleotide ambiguity (max 2 nucleotides allowed), adapter sequences, and length. Duplicate sequences were merged and aligned against the SILVA database v. 138 (https://www.arb-silva.de/ (accessed on 10 May 2023)) at a 97% identity threshold. Chimeric artifacts were removed and then OTU tables for taxonomy data collection were created. The samples were compared at a sequencing depth of 10,000 reads. The evaluation of diversity in microbial taxa within the groups (alpha diversity) was assessed by different available approaches: Chao-1 bias-corrected, Simpson’s index, and Shannon entropy [55,56]. Then, Principal coordinates analysis (PCoA) and Bray-Curtis method were used for diversity estimation between groups (beta diversity), in order to identify potential dissimilarities of OTU relative abundances among different groups [57]. Kruskal-Wallis test for alpha diversity and the PERMANOVA test for beta diversity (*p*-value ≤ 0.05) were applied. The OTU table (see results), showing the abundance at the taxonomic level of family in the analyzed samples, was then used to perform a generalized linear model test of differential abundance to compare microbial abundance between groups. The “Differential Abundance Analysis” tool embedded in CLC Genomic Workbench performs a Trimmed Mean by M-Values (TMM) normalization to make samples comparable, adjusting library sizes. The Wald test was used to determine significance between group pairs [58]. The main comparisons, for each sampling site (eye, gill, and skin) made by DAA were:

- Controls vs. Cafodos-treated after 3 h, separately for each sampling site;
- Controls vs. Cafodos-treated after 24 h, separately for each sampling site;
- Controls at 3 h vs. controls at 24 h, separately for each sampling site;
- Cafodos-treated at 3 h vs. Cafodos-treated after 24 h, separately for each sampling site;
- All controls at 3 h vs. all Cafodos-treated samples at 3 h (all sampling sites together);
- All controls at 24 h vs. all Cafodos-treated samples at 24 h (all sampling sites together).

The last two comparisons, grouping microbiomes of all the sampling sites together (gills + eye + skin), were carried out to check for common global changes in OTU abundances due to Cafodos exposure and/or the endogenous progression of spoilage in untreated control fish.

All sequencing data and related metadata are available in the Sequence Read Archive (SRA) data repository under the accession code PRJNA1064876.

### 4.4. Multivariate Data Analysis 

Multivariate statistics were applied to identify the effects played by the Cafodos treatment at both 3 h and 24 h treatment and separately for the four sampling sites taken into account (muscle, skin, gills, and eye). Data fusion methods were implemented to take advantage simultaneously of all the characterizations performed by the omics platforms.

Data fusion techniques [15] were applied using a low-level approach, i.e., combining the results of the different platforms together: a first analysis was performed by multiple factor analysis (MFA) [59,60], followed by partial least squares discriminant analysis (PLS-DA) coupled to backward elimination variable selection (BE-PLS-DA) on the dataset consisting of all platforms together. In the case of muscle and skin, metabolomic, lipidomic, and proteomic characterizations were combined, while, for gills, metabolomic and proteomic characterizations were used, and eye was characterized only by metabolomics. 

MFA is similar to PCA [61], an unsupervised method that allows the identification of a new reference system, given by new variables called principal components (PC), linear combinations of the original variables. Each PC is aligned along the direction of maximum residual variance (maximum information) of the data; therefore, they are calculated hierarchically (they contain a decreasing amount of information), and they are orthogonal to each other. MFA [59,60] is also an unsupervised pattern recognition method, similar to PCA, where the variables are divided into groups (blocks): it is, therefore, a method allowing to tackle “data fusion” problems, i.e., merging together sample characterizations from different sources. Here, MFA was used to carry out a global multi-omics characterization of each sampling site investigated. In MFA, each source (variable block) is weighted by the inverse of the eigenvalue of the first PC calculated by applying a PCA on the data coming only from that source: in this way, each source brings the same amount of information to the global description. In MFA it is also possible to include both quantitative and qualitative variables: the former are treated through PCA, while the latter through a correspondence analysis. The results can be evaluated in a similar way to what is completed for PCA, i.e., through score and loading plots: the score plot represents the projections of the samples in the space given by the PCs (or the MFA dimensions) and can be used to identify groups of samples with similar or opposite behaviors, and also possible outliers; the loading plot represents the original variables in the space given by the PCs and can be used to identify correlations among the variables and the reasons of the grouping observed in the corresponding score plot. In the case of MFA, contribution plots can also be evaluated, reporting each block variable in the space given by the most relevant dimensions calculated.

PLS-DA [61] exploits the PLS regression for classification problems. PLS searches for pairs of latent variables (LVs, similar to the principal components) calculated on the descriptors (X variables) and on the responses (Y variables, here class belonging) that best correlate with each other. The method can be modified for application to classification studies using Y as a variable that describes the belonging of the samples to different classes. PLS-DA was applied here, coupled with an iterative variable selection method, in backward elimination (BE-PLS-DA), which allowed a low-level data fusion, when necessary (i.e., for muscle, skin, and gills): the different omics sources were in fact “fused” together and BE-PLS-DA was applied to obtain the best discriminant models capable of separating the samples into the classes present with the best prediction performance and taking into account all the characterizations simultaneously. BE-PLS-DA was applied iteratively eliminating at each cycle the variables characterized by the minimum VIP score [62]; the calculations were performed in venetian blind cross-validation with 6 deletion groups (the control and the treated sample related to the same sampling day were eliminated at each cycle); the best model was chosen based on the best prediction error.

### 4.5. Software 

Multivariate analysis was carried out by homemade routines developed in Matlab (R2014, The Mathworks, Natick, MA, USA). PLS-DA models were calculated by the Classification Toolbox [63] (Milano Chemometrics Group, Milan, Italy), developed in Matlab. All graphical representations were carried out by Statistica v.7 (Statsoft Inc., Tulsa, OK, USA) and Excel 2016 (Microsoft Corporation, Redmond, WA, USA).

## 5. Conclusions

The paper deals with the identification of short- and long-term illicit conservation treatments by a Cafodos solution on sea bass. The approach takes advantage of a multi-omic characterization of the collected samples, coupled to data fusion chemometric methods for the identification of the main molecules involved in the studied effect. The adopted approach proved to be effective in the identification of the molecules responsible for the effect played by Cafodos at both short- (3 h) and long-term (24 h). The application of data fusion methods allowed the identification of panels of molecules able to discriminate control from treated samples. Both the models built from single sources and from fused data highlighted a different effect played by Cafodos in the short- and long-term. The fact that the effect is in general different in the short- and long-term can be also due to the study design, where each treated group (3 h or 24 h) was compared to the corresponding controls: in the short-term, this corresponds to the comparison between treated and fresh samples since the storage on ice is very short, while at 24 h the same comparison corresponds to the evaluation of the effect played by Cafodos vs. a normal storage on ice. 

For proteomics, an overall inhibition effect of the proteolytic/degradative phenomena was recorded, in agreement with the inhibitory effects on proteolysis exerted by peroxides accounted for in the literature. In particular, some interesting effects were pointed out when investigating the proteins identified as deregulated after treatment at the different sampling sites. In the case of gills, effects (3 h) were identified acting on: the residual redox potential (Grx), the regulation of autophagy (temporary increase in TANK-binding kinase 1), the inhibition of spontaneous proteolysis (higher levels of cystatins), ion transponders (NKCC1) and the delay of microbial proliferation (decrease in IMPA1.1 and MIPS). 

In the case of skin instead, effects on heat shock proteins (HSP90) were identified (3 h) and a general stabilization of proteins (higher MLC2 level at 24 h), while in muscle, a delay in the degradation of muscular proteins was identified (higher myomesin and myosin heavy chain levels and an increase in annexins after 24 h).

The validation of the identified proteins as biomarkers related to Cafodos exposure could therefore be extended in the future considering multiple fish species and/or large field sample collection programs.

In the case of lipidomics, complex changes have been identified at both 3 h and 24 h treatments for all the sampling sites investigated. The results show a deep alteration of the lipid metabolism with both the increase or decrease in different classes of lipids and different effects for short- and long-term treatment. Among the different analytes, EPA, DHA, palmitic, and oleic acid showed a decrease after the 3 h treatment (DHA also at 24 h), while stearic acid showed an increase at 3 h. 

The alteration of different classes of metabolites could be correlated with the alterations observed for lipids: the rates of oxidation and degradation of lipids can indeed bring higher levels of aldehydes, ketones, and amines, favored by residual catalytic activities of endogenous enzymes and by microbial proliferation.

Further validation of metabolomics profiles collected on Cafodos treated and untreated sea bass specimens will be needed to refine and further filter the redundancies in biological information that are often a consequence of the application of untargeted omics techniques.

The procedure here applied, involving a complex multi-omic sample characterization coupled with multivariate analysis and data fusion with variable selection, allows the identification of the best set of candidate biomarkers of the applied illicit treatment, thus obtaining the best compromise between exhaustivity of the biomarker search and biological redundancy avoidance. In light of the recorded results by extensive application of untargeted lipidomics and metabolomics, future development of simpler targeted methods to directly estimate ratios and abundances of selected biomarkers (e.g., EPA, DHA, palmitic, oleic, and stearic acid) in sea bass, coupled with a dedicated multivariate model, would provide to both the competent authorities and the fish industries, quicker evaluation tools when the illicit use of Cafodos is suspected. 

The effect played by Cafodos involved not only proteins, lipids, and small molecules, but also microbial perturbations among the different sampling sites considered in the study. Also, if the basal microbial heterogeneity did not provide a clear identification of specific OTUs that can be related to Cafodos exposure, it however allowed a detailed description of the complex microbiological environment related to spoilage. On the other hand, as already stated, the proteins, lipids, and metabolic biomarkers identified by multi-omics have potential applications for the identification of Cafodos and other similar illicit treatments: in this perspective, the use of a non-invasive sampling strategy also improves the procedure, making it easily applicable for on-site controls directly on the market. 

## Figures and Tables

**Table 1:** Abundances of the most represented families in the analyzed samples divided by sampling site (gill, eye, and skin).

Gill		Eye		Skin	
Family	Abundance (%)	Family	Abundance (%)	Family	Abundance (%)
Flavobacteriaceae	39.00	Pseudoalteromonadaceae	49.00	Moraxellaceae	38.00
Vibrionaceae	30.00	Comamonadaceae	19.00	Shewanellaceae	21.00
Pseudoalteromonadaceae	20.00	Shewanellaceae	13.00	Pseudoalteromonadaceae	19.00
Shewanellaceae	7.00	Moraxellaceae	12.00	Comamonadaceae	13.00
Moraxellaceae	2.00	Flavobacteriaceae	2.00	Flavobacteriaceae	5.00
Comamonadaceae	2.00	Xanthobacteraceae	1.00	Pseudomonadaceae	1.00
Moritellaceae	0.23	Propionibacteriaceae	0.82	Propionibacteriaceae	1.00
Rhodobacteraceae	0.18	Corynebacteriaceae	0.69	Xanthobacteraceae	0.70
Hydrogenophilaceae	0.12	Moritellaceae	0.63	Vibrionaceae	0.22
Rickettsiaceae	0.04	Pseudomonadaceae	0.39	Micrococcaceae	0.15

**Table 2:** Differential abundance analysis (DAA) of Cafodos-treated groups (eye and skin specimens) after 24 h of exposure.

Tissue	Family	Max Group Mean	Log_2_ Fold Change	Fold Change	*p*-Value	FDR *p*-Value
Eye	Xanthobacteraceae	8.1 × 10^2^	6.26	77	0.0000	0.0005
	Comamonadaceae	1.2 × 10^3^	6.05	66	0.0001	0.0013
	Oxalobacteraceae	1.0 × 10^2^	5.19	37	0.0017	0.0092
	Corynebacteriaceae	5.3 × 10^2^	9.20	5.9 × 10^2^	0.0041	0.0147
	Propionibacteriaceae	4.4 × 10^2^	3.96	16	0.0042	0.0147
	Rhizobiaceae	1.4 × 10^2^	10.43	1.4 × 10^3^	0.0089	0.0234
Skin	Flavobacteriaceae	3.5 × 10^2^	6.53	92	0.0040	0.0228
	Pseudoalteromonadaceae	4.2 × 10^2^	3.22	9.3	0.0042	0.0228

**Table 3:** Results for BE-PLS-DA: for each sampling site and each treatment time (3 h or 24 h) the global model (containing the data from all the omics sources) is compared to the model obtained for single sources. For each model, the number of LVs included in the final model (N° LVs) and the accuracy of classification both in fitting (Acc% Cal) and in cross-validation (Acc% cv) are provided. Numbers in parentheses in the second and third column represent the number of original and selected variables, respectively.

Sampling Site	Omics Sources and (Original Variables)	Omics Sources and (Selected Variables)	N° LVs	Acc% Cal	Acc% cv
Muscle 3 h	Lipid (921) + Metab (464) + Prot (66)	Lipid (45) + Metab (16) + Prot (1)	1	100	100
	Lipid (921)	Lipid (18)	1	100	100
	Metab (464)	Metab (14)	1	100	100
	Prot (66)	Prot (6)	1	100	100
Muscle 24 h	Lipid (921) + Metab (464) + Prot (66)	Lipid (11) + Metab (13) + Prot (3)	1	100	100
	Lipid (921)	Lipid (27)	1	100	100
	Metab (464)	Metab (15)	1	100	100
	Prot (66)	Prot (6)	1	100	100
Skin 3 h	Lipid (944) + Metab (384) + Prot (99)	Lipid (26) + Metab (26) + Prot (1)	1	100	100
	Lipid (944)	Lipid (18)	1	100	100
	Metab (384)	Metab (24)	1	100	100
	Prot (99)	Prot (12)	1	100	100
Skin 24 h	Lipid (944) + Metab (384) + Prot (99)	Lipid (64) + Metab (69) + Prot (14)	1	100	100
	Lipid (944)	Lipid (12)	1	100	100
	Metab (384)	Metab (34)	1	100	100
	Prot (99)	Prot (4)	1	100	100
Gills 3 h	Metab (380) + Prot (108)	Metab (59) + Prot (14)	1	100	100
	Metab (380)	Metab (69)	1	100	100
	Prot (108)	Prot (12)	1	100	100
Gills 24 h	Metab (380) + Prot (108)	Metab (7) + Prot (1)	1	100	100
	Metab (380)	Metab (14)	1	100	100
	Prot (108)	Prot (14)	1	100	100
Eye 3 h	Metab (340)	Metab (69)	1	100	100
Eye 24 h	Metab (340)	Metab (21)	1	100	100

## Data Availability

Metagenomic data are available in the SRA data repository under the accession code PRJNA1064876. Other data are available on request from the authors.

## Supplementary Materials

The following supporting information can be downloaded at: https://www.mdpi.com/article/10.3390/ijms25031509/s1.

## References

[1] Purcell S.W., Crona B.I., Lalavanua W., Eriksson H. (2017). Distribution of economic returns in small-scale fisheries for international markets: A value-chain analysis. Mar. Policy.

[2] Cooney R., de Sousa D.B., Fernández-Ríos A., Mellett S., Rowan N., Morse A.P., Hayes M., Laso J., Regueiro L., Wan A.H. (2023). A circular economy framework for seafood waste valorisation to meet challenges and opportunities for intensive production and sustainability. J. Clean. Prod..

[3] Ye B., Chen J., Fu L., Wang Y. (2022). Application of nondestructive evaluation (NDE) technologies throughout cold chain logistics of seafood: Classification, innovations and research trends. Lebensm.-Wiss. Technol..

[4] Esposito G., Sciuto S., Guglielmetti C., Pastorino P., Ingravalle F., Ru G., Bozzetta E.M., Acutis P.L. (2022). Discrimination between Wild and Farmed Sea Bass by Using New Spectrometry and Spectroscopy Methods. Foods.

[5] Pennisi F., Giraudo A., Cavallini N., Esposito G., Merlo G., Geobaldo F., Acutis P.L., Pezzolato M., Savorani F., Bozzetta E. (2021). Differentiation between fresh and thawed cephalopods using nir spectroscopy and multivariate data analysis. Foods.

[6] Currò S., Balzan S., Novelli E., Fasolato L. (2023). Cuttlefish Species Authentication: Advancing Label Control through Near-Infrared Spectroscopy as Rapid, Eco-Friendly, and Robust Approach. Foods.

[7] Zhou J., Wu X., Chen Z., You J., Xiong S. (2019). Evaluation of freshness in freshwater fish based on near infrared reflectance spectroscopy and chemometrics. Lebensm.-Wiss. Technol..

[8] Xiang Y., Sun C., Zhao Y., Li L., Yang X., Wu Y., Chen S., Wei Y., Li C., Wang Y. (2022). Label-Free Proteomic Analysis Reveals Freshness-Related Proteins in Sea Bass (*Lateolabrax japonicus*) Fillets Stored on Ice. Lebensm.-Wiss. Technol..

[9] Massaro A., Stella R., Negro A., Bragolusi M., Miano B., Arcangeli G., Biancotto G., Piro R., Tata A. (2021). New Strategies for the Differentiation of Fresh and Frozen/Thawed Fish: A Rapid and Accurate Non-Targeted Method by Ambient Mass Spectrometry and Data Fusion (Part A). Food Control.

[10] Stella R., Mastrorilli E., Pretto T., Tata A., Piro R., Arcangeli G., Biancotto G. (2022). New Strategies for the Differentiation of Fresh and Frozen/Thawed Fish: Non-Targeted Metabolomics by LC-HRMS (Part B). Food Control.

[11] Moser B., Jandrić Z., Troyer C., Priemetzhofer L., Domig K.J., Jäger H., Sabrina, Mayer H., Hann S., Zitek A. (2023). Evaluation of Spectral Handheld Devices for Freshness Assessment of Carp and Trout Fillets in Relation to Standard Methods Including Non-Targeted Metabolomics. Food Control.

[12] Manimaran U., Shakila R.J., Shalini R., Sivaraman B., Sumathi G., Selvaganapathi R., Jeyasekaran G. (2015). Effect of Additives in the Shelflife Extension of Chilled and Frozen Stored Indian Octopus (*Cistopus indicus*). J. Food Sci. Technol..

[13] Muscarella M., Lo Magro S., Campaniello M., Armentano A., Stacchini P. (2013). Survey of Histamine Levels in Fresh Fish and Fish Products Collected in Puglia (Italy) by ELISA and HPLC with Fluorimetric Detection. Food Control.

[14] Manfredi M., Barberis E., Gosetti F., Conte E., Gatti G., Mattu C., Robotti E., Zilberstein G., Koman I., Zilberstein S. (2017). Method for Noninvasive Analysis of Proteins and Small Molecules from Ancient Objects. Anal. Chem..

[15] Ballabio D., Robotti E., Grisoni F., Quasso F., Bobba M., Vercelli S., Gosetti F., Calabrese G., Sangiorgi E., Orlandi M. (2018). Chemical profiling and multivariate data fusion methods for the identification of the botanical origin of honey. Food Chem..

[16] Robotti E., Belay M.H., Calà E., Benedetto A., Cerruti S., Pezzolato M., Pennisi F., Abete M.C., Marengo E., Brizio P. (2023). Identification of Illicit Conservation Treatments in Fresh Fish by Micro-Raman Spectroscopy and Chemometric Methods. Foods.

[17] Dai S., Lin Z., Xu B., Wang Y., Shi X., Qiao Y., Zhang J. (2018). Metabolomics Data Fusion between near Infrared Spectroscopy and High-Resolution Mass Spectrometry: A Synergetic Approach to Boost Performance or Induce Confusion. Talanta.

[18] Benedetto A., Pezzolato M., Biasibetti E., Bozzetta E. (2021). Omics Applications in the Fight against Abuse of Anabolic Substances in Cattle: Challenges, Perspectives and Opportunities. Curr. Opin. Food Sci..

[19] Ogata F.T., Branco V., Vale F.F., Coppo L. (2021). Glutaredoxin: Discovery, redox defense and much more. Redox Biol..

[20] Illikoud N., Gohier R., Werner D., Barrachina C., Roche D., Jaffrès E., Zagorec M. (2019). Transcriptome and Volatilome Analysis during Growth of Brochothrix Thermosphacta in Food: Role of Food Substrate and Strain Specificity for the Expression of Spoilage Functions. Front. Microbiol..

[21] Yu T., Yi Y.S., Yang Y., Oh J., Jeong D., Cho J.Y. (2012). The pivotal role of TBK1 in inflammatory responses mediated by macrophages. Mediat. Inflamm..

[22] Chen J., Zhou X.-Y., Li P., Li Z.-C., Zhang C., Sun Y.-H., Wang G.-Y., Chen D.-D., Lu L.-F., Li S. (2021). Molecular characterization of a cyprinid fish (*Ancherythroculter nigrocauda*) TBK1 and its kinase activity in IFN regulation. Dev. Comp. Immunol..

[23] González-Blanco L., Sierra V., Diñeiro Y., Coto-Montes A., Oliván M. (2023). Role of the Endoplasmic Reticulum in the Search for Early Biomarkers of Meat Quality. Meat Sci..

[24] Kuang G., Tao W., Zheng S., Wang X., Wang D. (2020). Genome-wide identification, evolution and expression of the complete set of cytoplasmic ribosomal protein genes in nile tilapia. Int. J. Mol. Sci..

[25] Wickramasinghe P.D.S.U., Kwon H., Elvitigala D.A.S., Wan Q., Lee J. (2020). Identification and characterization of cystatin B from black rockfish, Sebastes schlegelii, indicating its potent immunological importance. Fish Shellfish. Immunol..

[26] Rajan B., Fernandes J.M., Caipang C.M., Kiron V., Rombout J.H., Brinchmann M.F. (2011). Proteome Reference Map of the Skin Mucus of Atlantic Cod (*Gadus morhua*) Revealing Immune Competent Molecules. Fish Shellfish. Immunol..

[27] Pinto R.D., Moreira A.R., Pereira P.J.B., dos Santos N.M.S. (2013). Molecular cloning and characterization of sea bass (*Dicentrarchus labrax* L.) calreticulin. Fish Shellfish. Immunol..

[28] Zhang Y., Liu L., Jin L., Yi X., Dang E., Yang Y., Li C., Gao T. (2014). Oxidative Stress–Induced Calreticulin Expression and Translocation: New Insights into the Destruction of Melanocytes. J. Investig. Dermatol..

[29] Bossus M., Charmantier G., Blondeau-Bidet E., Valletta B., Boulo V., Lorin-Nebel C. (2013). The ClC-3 chloride channel and osmoregulation in the European Sea Bass, Dicentrarchus labrax. J. Comp. Physiol. B.

[30] Bodinier C., Boulo V., Lorin-Nebel C., Charmantier G. (2009). Influence of salinity on the localization and expression of the CFTR chloride channel in the ionocytes of Dicentrarchus labrax during ontogeny. J. Anat..

[31] Giffard-Mena I., Lorin-Nebel C., Charmantier G., Castille R., Boulo V. (2008). Adaptation of the sea-bass (Dicentrarchus labrax) to fresh water: Role of aquaporins and Na+/K+-ATPases. Comp. Biochem. Physiol.-A.

[32] Boutet I., Long Ky C.L., Bonhomme F. (2006). A transcriptomic approach of salinity response in the euryhaline teleost, Dicentrarchus labrax. Gene.

[33] Wang X., Kültz D. (2017). Osmolality/salinity-responsive enhancers (OSREs) control induction of osmoprotective genes in euryhaline fish. Proc. Natl. Acad. Sci. USA.

[34] Kolbeck S., Ludwig C., Meng C., Hilgarth M., Vogel R.F. (2020). Comparative Proteomics of Meat Spoilage Bacteria Predicts Drivers for Their Coexistence on Modified Atmosphere Packaged Meat. Front. Microbiol..

[35] Han B., Luo J., Jiang P., Li Y., Wang Q., Bai Y., Chen J., Wang J., Zhang J. (2020). Inhibition of Embryonic HSP 90 Function Promotes Variation of Cold Tolerance in Zebrafish. Front. Genet..

[36] Georgiou S., Makridis P., Dimopoulos D., Power D.M., Mamuris Z., Moutou K.A. (2014). Myosin light chain 2 isoforms in gilthead sea bream (*Sparus aurata* L.): Molecular growth markers at early stages. Aquaculture.

[37] Mato A., Rodríguez-Vázquez R., López-Pedrouso M., Bravo S., Franco D., Zapata C. (2019). The First Evidence of Global Meat Phosphoproteome Changes in Response to Pre-Slaughter Stress. BMC Genom..

[38] Tian Y., Wen H., Qi X., Mao X., Shi Z., Li J., He F., Yang W., Zhang X., Li Y. (2019). Analysis of apolipoprotein multigene family in spotted sea bass (*Lateolabrax maculatus*) and their expression profiles in response to Vibrio harveyi infection. Fish Shellfish. Immunol..

[39] Xu J., Gao J., Li J., Xue L., Clark K.J., Ekker S.C., Du S.J. (2012). Functional Analysis of Slow Myosin Heavy Chain 1 and Myomesin-3 in Sarcomere Organization in Zebrafish Embryonic Slow Muscles. J. Genet. Genom..

[40] He Y., Huang H., Li L., Yang X., Hao S., Chen S., Deng J. (2018). The effects of modified atmosphere packaging and enzyme inhibitors on protein oxidation of tilapia muscle during iced storage. Lebensm.-Wiss.-Technol..

[41] Kjaersgård I., Nørrelykke M., Jessen F. (2006). Changes in cod muscle proteins during frozen storage revealed by proteome analysis and multivariate data analysis. Proteomics..

[42] Sun X., Guo X., Ji M., Wu J., Zhu W., Wang J., Cheng C., Chen L., Zhang Q. (2019). Preservative effects of fish gelatin coating enriched with CUR/βCD emulsion on grass carp (*Ctenopharyngodon idellus*) fillets during storage at 4 °C. Food Chem..

[43] Hwang H.J., Moon C.H., Kim H.G., Kim J.Y., Lee J.M., Park J.W., Chung D.K. (2007). Identification and Functional Analysis of Salmon Annexin 1 Induced by a Virus Infection in a Fish Cell Line. J. Virol..

[44] Dang D., Zhai C., Nair M.N., Thornton K.J., Sawalhah M.N., Matarneh S.K. (2022). Tandem Mass Tag Labeling to Assess Proteome Differences between Intermediate and Very Tender Beef Steaks. J. Anim. Sci..

[45] Olivares-Rubio H.F., Vega-López A. (2016). Fatty acid metabolism in fish species as a biomarker for environmental monitoring. Environ. Pollut..

[46] Alasalvar C., Taylor K.D.A., Zubcov E., Shahidi F., Alexis M. (2002). Differentiation of cultured and wild sea bass (*Dicentrarchus labrax*): Total lipid content, fatty acid and trace mineral composition. Food Chem..

[47] Reyes M., Rodríguez M., Montes J., Barroso F.G., Fabrikov D., Morote E., Sánchez-Muros M.J. (2020). Nutritional and Growth Effect of Insect Meal Inclusion on Seabass (*Dicentrarchus labrax*) Feeds. Fishes.

[48] Montero D., Carvalho M., Terova G., Fontanillas R., Serradell A., Ginés R., Tuset V.M., Acosta F., Rimoldi S., Bajek A. (2023). Nutritional Innovations in Superior European Sea Bass (*Dicentrarchus labrax*) Genotypes: Implications on Fish Performance and Feed Utilization. Aquaculture.

[49] Syropoulou F., Anagnostopoulos D.A., Parlapani F.F., Karamani E., Stamatiou A., Tzokas K., Nychas G.E., Boziaris I.S. (2022). Microbiota Succession of Whole and Filleted European Sea Bass (*Dicentrarchus labrax*) during Storage under Aerobic and MAP Conditions via 16S RRNA Gene High-Throughput Sequencing Approach. Microorganisms.

[50] Thomas A., Konteles S.J., Ouzounis S., Papatheodorou S., Tsakni A., Houhoula D., Tsironi T. (2023). Bacterial Community in Response to Packaging Conditions in Farmed Gilthead Seabream. Aquac. Fish..

[51] Rosado D., Pérez-Losada M., Severino R., Cable J., Xavier R. (2019). Characterization of the Skin and Gill Microbiomes of the Farmed Seabass (*Dicentrarchus labrax*) and Seabream (*Sparus aurata*). Aquaculture.

[52] Raudvere U., Kolberg L., Kuzmin I., Arak T., Adler P., Peterson H., Vilo J. (2019). G:Profiler: A Web Server for Functional Enrichment Analysis and Conversions of Gene Lists (2019 Update). Nucleic Acids Res..

[53] Caporaso J.G., Lauber C.L., Walters W.A., Berg-Lyons D., Huntley J., Fierer N., Owens S.M., Betley J., Fraser L., Bauer M. (2012). Ultra-High-Throughput Microbial Community Analysis on the Illumina HiSeq and MiSeq Platforms. ISME J..

[54] Klindworth A., Pruesse E., Schweer T., Peplies J., Quast C., Horn M., Glöckner F.O. (2013). Evaluation of General 16S Ribosomal RNA Gene PCR Primers for Classical and Next-Generation Sequencing-Based Diversity Studies. Nucleic Acids Res..

[55] Gauthier J., Derome N. (2021). Evenness-Richness Scatter Plots: A Visual and Insightful Representation of Shannon Entropy Measurements for Ecological Community Analysis. mSphere.

[56] Kim B.R., Shin J., Guevarra R.B., Lee J.H., Kim D.W., Seol K.H., Lee J.H., Kim H.B., Isaacson R.E. (2017). Deciphering Diversity Indices for a Better Understanding of Microbial Communities. J. Microbiol. Biotechnol..

[57] Gail M.H., Wan Y., Shi J. (2021). Power of Microbiome Beta-Diversity Analyses Based on Standard Reference Samples. Am. J. Epidemiol..

[58] Andreani A., Beltramo C., Ponzetta M.P., Belcari A., Sacchetti P., Acutis P.L., Peletto S. (2023). Analysis of the Bacterial Communities Associated with Pupae and Winged or Wingless Adults of *Lipoptena Fortisetosa* Collected from Cervids in Italy. Med. Vet. Entomol..

[59] Abascal E., de Rada V.D., Lautre I.G., Landaluce M.I. (2013). Extending dual multiple factor analysis to categorical tables. J. Appl. Stat..

[60] Abdi H., Williams L.J., Valentin D. (2013). Multiple factor analysis: Principal component analysis for multitable and multiblock data sets. Wiley Interdiscip. Rev. Comput. Stat..

[61] Benedetto A., Pezzolato M., Robotti E., Biasibetti E., Poirier A., Dervilly G., Le Bizec B., Marengo E., Bozzetta E. (2021). Profiling of transcriptional biomarkers in FFPE liver samples: PLS-DA applications for detection of illicit administration of sex steroids and clenbuterol in veal calves. Food Control.

[62] Oussama A., Elabadi F., Platikanov S., Kzaiber F., Tauler R. (2012). Detection of olive oil adulteration using FT-IR spectroscopy nd PLS with variable importance of projection (VIP) scores. J. Am. Oil Chem. Soc..

[63] Ballabio D., Consonni V. (2013). Classification tools in chemistry. Part 1: Linear models. PLS-DA. Anal. Methods.

