# Supplementary material for: Multi-Omics Approaches for Freshness Estimation and Detection of Illicit Conservation Treatments in Sea Bass (Dicentrarchus Labrax): Data Fusion Applications
Source: Int J Mol Sci. 2024 Jan 26;25(3):1509. doi: 10.3390/ijms25031509 (PMC10855268; doi:10.3390/ijms25031509)
Supplement: Supplementary file 1 [file ijms-25-01509-s001.zip › Supplementary Materials S3.pdf]

Multi-Omics Approaches for Freshness Estimation and Detection of Illicit Conservation Treatments in Sea Bass (*Dicentrarchus Labrax*): Data Fusion Applications

Alessandro Benedetto, Elisa Robotti, Masho Hilawie Belay, Arianna Ghignone, Alessia Fabbris, Eleonora Goggi, Simone Cerruti, Marcello Manfredi, Elettra Barberis, Simone Peletto, Alessandra Arillo, Nunzia Giaccio, Maria Angela Masini, Jessica Brandi, Daniela Cecconi, Emilio Marengo and Paola Brizio

Supplementary Material S3.

Here are reported with more details all the discriminant molecules identified by the BE-PLS-DA models built for each matrix and for each time of treatment for global models and for single source models. For each model the name of each molecule included in the model, the corresponding label adopted in the corresponding figures (Figure 4 and 5) and the value of the regression coefficient is given. Positive coefficients correspond to molecules more present after treatment, while negative coefficients correspond to molecules decreasing after treatment.

GLOBAL MODELS

Table S2.1: Muscle 3h

Source	Compound Name (Lipids)	Label	Coefficient
Lipidomics	CAR5:0	L13	-0,0096
	Cer38:1;4O|Cer14:0;3O/24:1;(2OH)	L22	0,011
	Cer39:2;2O|Cer21:0;2O/18:2	L26	0,012
	Cer45:6;2O|Cer21:1;2O/24:5	L37	0,011
	Cer47:6;2O|Cer21:1;2O/26:5	L38	0,011
	DG28:2	L45	0,010
	DG29:3	L47	0,011
	DG31:0	L51	0,0097
	DG31:3	L53	0,010
	DG32:3	L56	0,011
	DG35:5	L68	0,011
	DG38:2|DG19:1_19:1	L90	0,012
	FA22:2;4O	L133	-0,011
	FA28:7	L142	-0,010
	FA30:5	L143	-0,011
	FA30:7	L144	-0,011
	FA32:5	L145	-0,0097

	FA34:7	L149	0,011
	FA36:10	L150	0,011
	FA36:7	L153	0,012
	FA44:10	L162	-0,010
	HexCer42:2;2O|HexCer18:1;2O/24:1	L168	0,011
	LPCO-18:2	L197	-0,011
	LPE20:1	L203	-0,010
	NAE22:3	L232	0,011
	NAE22:4	L233	0,0096
	NAE24:4	L235	0,011
	PE39:7;O|PE22:6_17:1;O	L445	0,0084
	SM32:0;3O	L557	0,011
	TG38:3|TG9:0_9:0_20:3	L602	0,011
	TG40:2|TG9:0_9:0_22:2	L604	0,010
	TG40:5|TG9:0_9:0_22:5	L605	0,011
	TG46:2|TG12:0_16:0_18:2	L622	0,011
	TG48:5|TG14:0_16:1_18:4	L648	-0,0087
	TG48:9|TG16:1_16:4_16:4	L655	0,010
	TG54:4|TG16:0_18:1_20:3	L763	-0,0088
	TG57:10|TG17:1_18:3_22:6	L817	-0,0095
	TG58:0|TG16:0_17:0_25:0	L830	0,0098
	TG60:10|TG20:1_18:3_22:6	L874	-0,010
	TG61:1|TG16:0_27:0_18:1	L892	-0,010
	TG62:0|TG16:0_22:0_24:0	L896	-0,010
	TG62:1|TG16:0_28:0_18:1	L897	0,0097
	TG62:13|TG18:1_22:6_22:6	L902	-0,011
	TG62:14|TG18:2_22:6_22:6	L904	-0,0099
	TG64:13|TG20:1_22:6_22:6	L915	-0,010
Metabolomics	4,7,10,13,16,19-Docosahexaenoic acid, methyl ester, (all-Z)- SUM 79+106	M123	-0,0098
	6,9,12,15-Docosatetraenoic acid, methyl ester	M141	0,0089
	Androstane, (5á)-	M180	0,0090
	Benzene, (1-hexyloctyl)-	M202	0,0096
	benzoic acid, 3,5-dimethyl-, trimethylsilyl ester	M220	0,011
	cis-7-Hexadecenoic acid SUM 292+280+236	M250	0,0096
	cis-9-Hexadecenoic acid, heptyl ester	M251	0,010
	Desmethylverapamil	M275	0,0099
	Desmethylverapamil SUM	M276	0,0099
	Nonanal	M390	-0,011
	Octadecanoic acid, ethyl ester	M399	0,0098

	s-Indacen-1(2H)-one, 3,5,6,7-tetrahydro-3,3,4,5,5,8-hexamethyl-	M437	0,0095
	Squalene SUM 123+217	M438	0,0088
	Trimethylolpropane, 3TMS derivative	M456	0,0086
Proteomics	Slow myosin heavy chain 2	E6ZIC8	0,0099

Table S2.2: Muscle 24h

Source	Compound Name (Lipids)	Label	Coefficient
Lipidomics	Cer41:0;4O I Cer21:0;3O/20:0;(2OH)	L30	0,026
	DG36:0	L71	0,029
	SM34:0;2O I SM21:0;2O/13:0	L561	-0,025
	SM34:0;3O	L562	-0,025
	TG40:0 I TG10:0_14:0_16:0	L603	-0,024
	TG46:4 I TG14:0_16:0_16:4	L626	-0,026
	TG49:5 I TG14:0_15:0_20:5	L663	-0,023
	TG52:0 I TG16:0_18:0_18:0	L707	0,026
	TG57:6 I TG17:0_18:1_22:5	L826	-0,023
	TG58:12 I TG18:2_18:4_22:6	L840	-0,025
	TG59:0 I TG16:0_17:0_26:0	L859	0,025
Metabolomics	1-(3-Methylbutyl)-2,3,6-trimethylbenzene	M10	0,023
	1-Octene, 3,7-dimethyl-	M49	0,029
	1-Propene-1,2,3-tricarboxylic acid, tributyl ester	M52	0,024
	2-Allyl-1,4-dimethoxybenzene	M81	0,025
	5alpha-Androstan-3alpha-ol, O-ethyl-	M138	-0,025
	9-Octadecenoic acid (Z)-, 2,3-dihydroxypropyl ester	M162	0,023
	Androstane, (5á)-	M179	-0,024
	Benzene, (1-methylundecyl)- SUM 246+105	M206	-0,026
	Eicosanoic acid, ethyl ester	M296	0,024
	Heptanoic acid, TMS derivative SUM 187+60	M331	0,024
	Hexadecanenitrile	M333	-0,027
	Hydroquinone, 2TMS derivative	M346	0,024
	Pentadecanoic acid, butyl ester	M413	0,025
Proteomics	Slow myosin heavy chain 2	E6ZIC7	-0,025
	Inositol-1-monophosphatase	E6ZJ93	-0,023
	Apolipoprotein E (Fragment)	Q0GN72	0,024

Table S2.3: Skin 3h

Source	Compound Name (Lipids)	Label	Coefficient
Lipidomics	CAR16/0	L9	0,013
	CAR19/0	L12	-0,012
	CAR19/3	L13	-0,012
	DG34/7	L67	0,014
	DG37/1	L77	0,014
	DG42/11|DG20/5_22/6	L101	-0,013
	NAE22/4	L167	-0,011
	PC28/1	L174	-0,012
	PC40/7	L247	0,012
	PCO-36/3	L275	0,012
	PE38/5|PE16/0_22/5	L300	0,012
	PG47/5	L330	-0,012
	SM35/1/2O|SM19/1/2O/16/0	L349	0,011
	SM39/2/2O|SM24/2/2O/15/0	L354	-0,013
	SM40/2/2O|SM18/1/2O/22/1	L358	0,011
	SM42/2/2O|SM18/1/2O/24/1	L363	0,013
	FA16/0	L609	0,011
	FA34/9	L639	0,013
	FA36/7	L643	0,013
	FA42/5	L653	0,013
	FA44/5	L658	0,013
	PC34/2/3O|PC16/0_18/2/3O	L723	-0,012
	PCO-34/2|PCO-16/0_18/2	L798	-0,013
	PCO-36/2|PCO-18/0_18/2	L806	-0,012
	PCO-40/6|PCO-18/0_22/6	L826	-0,013
	SM42/3/2O|SM17/2/2O/25/1	L939	-0,011
Metabolomics	1,3,6,10-Cyclotetradecatetraene, 3,7,11-trimethyl-14-(1-methylethyl)-, [S-(E,Z,E,E)]- SUM 229+190	M14	-0,011
	11-Tricosene	M28	-0,012
	1-Dodecanamine, N,N-dimethyl-	M41	0,015
	1-Dodecanol, TMS derivative	M43	0,011
	1-Methyl-N,N-bis(trimethylsilyl)-4-[(trimethylsilyl)oxy]-1H-imidazol-2-amine	M46	0,012
	2,3,4-Trihydroxybutyric acid tetrakis(trimethylsilyl) deriv., (, (R*,R*)-) SUM 292	M67	0,013
	2-Aminomalonic acid, N,O,O,-TMS	M84	0,011
	2-Butenedioic acid, (E)-, 2TMS derivative	M86	0,014
	2-Heptanamine, N-(phenylmethylene)-	M89	0,014

	3,5-di-tert-Butyl-4-hydroxycinnamic acid	M104	-0,012
	3-Phenyl-2-trimethylsiloxypropene	M111	-0,011
	6H-Dibenzo[b,d]-pyran	M131	-0,012
	7,9-Di-tert-butyl-1-oxaspiro(4,5)deca-6,9-diene-2,8-dione	M132	-0,014
	7-Methoxy-3-(2-methoxyphenyl)-2-methylchromen-4-one	M134	-0,011
	Benzaldehyde, 2,4-dimethyl- SUM 63+134	M168	-0,012
	Benzoic Acid, TMS derivative	M188	-0,011
	D-(-)-Tagatofuranose, pentakis(trimethylsilyl) ether (isomer 2) SUM 217	M216	0,012
	D-Arabinopyranose, 4TMS derivative (isomer 2)	M218	0,012
	D-Fructose, 5TMS derivative	M220	0,011
	Glycol salicylate, TBDMS derivative	M263	0,014
	Heptanoic acid, TMS derivative SUM 187+60	M269	0,012
	Nonanoic acid, TMS derivative	M316	0,013
	Octadecanoic acid, ethenyl ester	M319	-0,012
	Octanoic acid, TMS derivative SUM 201+116	M322	0,011
	Pregna-1,4,6-triene-3,20-dione, 17-(acetyloxy)-6,16-dimethyl-, (16α)-	M343	-0,013
	Triacetin	M367	0,013
Proteomics	Apolipoprotein B-100	Q9I8I1	-0,012

Table S2.4: Skin 24h

Source	Compound Name (Lipids)	Label	Coefficient
Lipidomics	CAR12/2	L5	0,0046
	CAR19/0	L12	0,0053
	CAR21/0	L15	-0,0046
	Cer32/1/2O|Cer18/1/2O/14/0	L24	0,0044
	DG32/7	L61	0,0046
	DG36/1	L71	0,0054
	DG40/6	L93	-0,0049
	DG40/6|DG16/0_24/6	L94	-0,0047
	DGO-30/1|DGO-16/0_14/1	L104	-0,0042
	DGO-32/1|DGO-16/0_16/1	L105	-0,0041
	DGO-34/1|DGO-18/0_16/1	L106	-0,0056
	LPC20/5	L132	0,0048
	LPCO-14/0	L137	0,0040
	LPCO-14/1	L138	0,0042
	NAE22/5	L168	-0,0046

	PC30/1	L177	0,0040
	PC32/0	L181	0,0041
	PC37/7	L225	0,0058
	PCO-43/8	L292	0,0044
	PG41/5	L325	0,0044
	SM36/2/2O|SM23/2/2O/13/0	L351	0,0041
	TG43/1|TG13/0_14/0_16/1	L381	0,0057
	TG44/3|TG14/0_14/0_16/3	L386	0,0054
	TG50/7|TG14/0_16/2_20/5	L426	0,0042
	TG50/9|TG14/0_16/4_20/5	L428	0,0042
	TG51/4|TG16/1_17/1_18/2	L434	0,0051
	TG52/0|TG14/0_18/0_20/0	L439	-0,0061
	TG53/0|TG14/0_15/0_24/0	L461	-0,0063
	TG53/1|TG16/0_19/0_18/1	L462	0,0053
	TG53/1|TG17/0_17/0_19/1	L463	-0,0045
	TG54/0|TG16/0_18/0_20/0	L472	-0,0061
	TG54/8|TG14/0_18/2_22/6	L485	0,0041
	TG55/0|TG15/0_16/0_24/0	L490	-0,0051
	TG55/1|TG15/0_24/0_16/1	L491	-0,0042
	TG58/12|TG16/1_20/5_22/6	L540	0,0062
	TG59/1|TG16/0_25/0_18/1	L554	-0,0047
	TG59/12|TG17/1_20/5_22/6	L556	0,0040
	TG61/1|TG16/0_27/0_18/1	L579	0,0055
	TG62/2|TG28/0_16/1_18/1	L594	0,0045
	TG62/9|TG22/1_18/2_22/6	L599	0,0055
	FA16/4	L611	0,0048
	FA22/5	L621	0,0060
	FA24/5	L627	0,0055
	FA26/6	L628	0,0042
	FA26/7	L629	0,0067
	FA28/7	L630	0,0057
	FA30/5	L631	0,0048
	FA30/7	L632	0,0055
	FA32/6	L634	-0,0047
	FA32/7	L635	0,0051
	FA34/9	L639	0,0046
	FA36/5	L641	-0,0045
	FA36/6	L642	-0,0043
	FA38/5	L645	-0,0041

	FA40/11	L648	0,0053
	FA44/11	L657	0,0043
	FA44/7	L660	0,0043
	FAHFA40/5/O|FAHFA20/3/20/2/O	L662	0,0043
	NAE21/2	L705	0,0058
	PC36/5|PC16/1_20/4	L744	0,0049
	PEO-34/2|PEO-18/1_16/1	L868	-0,0055
	SL38/0/O	L913	0,0056
	SL40/0/2O	L915	0,0055
	ST27/1/O/S	L944	0,0053
Metabolomics	(E)-9-Octadecenoic acid ethyl ester	M4	-0,0044
	1,3-Propanediol, TMS derivative	M15	0,0046
	10-Heptadecenoic acid, (Z)-, TMS derivative SUM 325+327	M23	0,0053
	11-Octadecenoic acid, (E)-, TMS derivative	M27	0,0057
	1-Dodecanamine, N,N-dimethyl-	M41	-0,0047
	1-Monopalmitin, 2TMS derivative	M49	0,0055
	2(3H)-Furanone, 5-dodecyldihydro-	M60	0,0058
	2(3H)-Furanone, dihydro-5-tetradecyl-	M61	0,0044
	2-(Decanoyloxy)propane-1,3-diyl dioctanoate	M62	0,0049
	2,3-Dihydroxypropyl icosanoate, 2TMS derivative	M68	0,0042
	2,5-Cyclohexadiene-1,4-dione, 2,6-bis(1,1-dimethylethyl)-	M73	-0,0061
	2,5-Dihydroxyacetophenone, 2TBDMS derivative	M74	-0,0052
	2,5-Di-tert-butyl-4-((trimethylsilyl)oxy)phenol	M79	0,0053
	2-Heptanamine, N-(phenylmethylene)-	M89	0,0043
	2H-Pyran-2-one, tetrahydro-6-undecyl-	M90	0,0057
	2-Oleoylglycerol, 2TMS derivative	M96	0,0047
	4-Pyrimidinamine, N-(trimethylsilyl)-2-[(trimethylsilyl)oxy]-	M122	-0,0048
	7-Methyl-Z,Z-8,10-hexadecadien-1-ol acetate	M135	0,0053
	9,12-Octadecadien-1-ol, (Z,Z)-	M138	0,0050
	9,12-Octadecadienoic acid, methyl ester, (E,E)-	M142	0,0055
	9-Hexadecenoic acid	M145	0,0050
	9-Nonadecene SUM 266+97	M149	-0,0043
	9-Octadecenamide, (Z)- SUM 72+59	M151	0,0049
	9-Octadecenenitrile, (Z)-	M152	0,0048
	9-Octadecynenitrile	M154	0,0048
	9-Tricosene, (Z)-	M156	-0,0046
	Acetamide, N-ethyl-	M159	-0,0041
	Adrenaline, 4TMS derivative	M163	0,0040
	Arachidic acid, TMS derivative	M165	0,0066

	Benzaldehyde, 2,5-dimethoxy- SUM 166	M169	0,0053
	Benzyl alcohol, TMS derivative SUM 165+180	M191	0,0053
	Bicyclo[2.2.2]oct-5-en-2-yldimethylamine	M192	0,0062
	Bisphenol A monomethyl ether, TMS derivativeSUM 299+314	M194	-0,0040
	Cholest-3,5-diene	M200	0,0050
	D-(-)-Ribofuranose, tetrakis(trimethylsilyl) ether (isomer 2) SUM 337+217	M214	0,0047
	Decanoic acid, TMS derivative	M219	0,0048
	Diphenyl ether	M227	0,0051
	Docosanoic acid, ethyl ester	M230	-0,0049
	Dodecanamide	M232	0,0061
	E,E,Z-1,3,12-Nonadecatriene-5,14-diol	M239	0,0055
	Ethyl 9-hexadecenoate	M249	0,0045
	Ethyl iso-allocholate	M250	0,0050
	Gallacetophenone-4'-methylether, bis(trimethylsilyl) ether	M253	-0,0058
	Glycerol monostearate, 2TMS derivative	M258	0,0042
	Glycidyl (Z)-9-Heptadecenoate	M260	-0,0053
	Glycidyl palmitoleate SUM 116	M262	-0,0054
	Heptanoic acid, TMS derivative SUM 187+60	M269	0,0044
	Hexadecanoic acid, methyl ester	M272	0,0059
	Hexanoic acid, TMS derivative	M278	0,0044
	Hydracrylic acid, 2TMS derivative	M280	0,0052
	Lignoceric acid, TMS derivative	M291	-0,0047
	Methyl (3,4-dimethoxyphenyl)(hydroxy)acetate, TMS derivative SUM 239+298	M296	-0,0058
	Methyl stearate	M299	0,0053
	Monocaprin, 2TMS derivative	M303	0,0040
	Monolaurin, 2TMS derivative	M304	0,0064
	Niacin, TMS derivative	M313	0,0056
	Octadecanamide	M318	0,0048
	Octadecanoic acid, ethyl ester	M320	-0,0042
	Oleic Acid SUM 280+264	M323	0,0044
	Oleic Acid, (Z)-, TMS derivative SUM 339+117	M324	-0,0041
	Palmitic Acid, TMS derivative SUM 286+72+311+313+145+129+327+185+287	M331	-0,0050
	Phloroglucinol, O,O'-bis(trimethylsilyl)-	M339	0,0041
	Silicic acid, diethyl bis(trimethylsilyl) ester	M355	-0,0042
	ß-Eudesmol, TMS derivative	M358	0,0047
	Tridecanoic acid SUM 70+98+84	M369	-0,0041
	Tyrosine, 2TMS derivative	M376	0,0052

	Undecanoic acid, TMS derivative	M378	0,0049
	Urea, 2TMS derivative	M379	0,0052
	Z-14-Octadecen-1-ol acetate	M380	0,0042
Proteomics	Myosin heavy chain	Q2PMW9	0,0048
	Fructose-bisphosphate aldolase	E6ZH34	0,0041
	Glyceraldehyde-3-phosphate dehydrogenase	Q5I1Z5	0,0051
	Keratin, type I cytoskeletal 18	E6ZFH0	0,0047
	Complement component c3-1	F8R8R0	0,0041
	MN-Alpha1	A0A1L6V014	0,0046
	Lipocalin	E6ZIH5	0,0045
	Myosin light chain	E6ZH67	0,0052
	Serine/arginine-rich splicing factor 2	E6ZHT0	0,0044
	Tubulin alpha chain (Fragment)	Q7T1F9	-0,0051
	Ribosomal protein L5	Q0GN71	0,0048
	Tubulin beta chain	E6ZF69	-0,0040
	Cardiac muscle myosin heavy chain 6 alpha (Fragment)	D5JZI9	0,0061
	Carbonic anhydrase	E6ZJA5	0,0052

Table S2.5: Gills 3h

Source	Compound Name (Metabolites)	Label	Coefficient
Metabolomics	1,3-Dihydroxyacetone dimer, 4TMS derivative	M9	0,011
	13-Octadecenoic acid, (Z)-, TMS derivative	M24	0,0098
	1-Monopalmitin, 2TMS derivative	M43	-0,011
	1-Tetradecanol, TMS derivative	M51	-0,0095
	2,3-Dihydroxypropyl icosanoate, 2TMS derivative	M58	-0,0088
	2,4,7,9-Tetramethyl-5-decyne-4,7-diol, 2TMS derivative	M59	0,010
	2,5-Dimethoxybenzhydrazide	M64	-0,012
	2,6-Dihydroxyacetophenone, 2TMS derivative	M70	0,012
	2,6-Di-tert-butyl-4-hydroxy-4-methylcyclohexa-2,5-dien-1-one	M71	-0,0088
	2-Monolinolenoylglycerol 1,3-bis(trimethylsilyl) ether SUM 103	M82	-0,011
	3-(2-Hydroxyethyl)imidazole-2-thione	M89	0,0092
	3,5-di-tert-Butyl-4-hydroxyacetophenone	M91	0,0099
	3-Methylcyclopentanol, trimethylsilyl ether	M98	0,0096
	3-Octenoic acid, TMS derivative	M100	0,010
	4-tert-Butylphenol, TMS derivative	M110	0,0091
	9(E),11(E)-Conjugated linoleic acid SUM 280	M118	-0,010

	9,12-Octadecadienoic acid (Z,Z)-, TMS derivative SUM 337+309+338	M120	-0,0098
	9-Hexadecenoic acid, (Z)-, TBDMS derivative	M122	0,014
	9-Octadecenenitrile, (Z)-	M129	-0,012
	9-Tetradecenal, (Z)-	M134	0,013
	9-Tetradecenoic acid, (E)-, TMS derivative	M135	0,010
	Acetophenone	M137	0,013
	Anthranilic acid, 2TMS derivative	M142	0,011
	Arabinofuranose, 1,2,3,5-tetrakis-O-(trimethylsilyl)-	M145	-0,011
	Arachidic acid, TMS derivative	M147	0,012
	Arachidonic acid, TMS derivative SUM 188+286	M148	-0,0099
	Benzaldehyde, 2,5-dimethyl-	M157	-0,0091
	Benzene, (1-ethylnonyl)-	M160	-0,012
	Benzene, 1,3-bis(1,1-dimethylethyl)-	M169	0,0097
	Calealactone E	M181	-0,0092
	Carbonic acid, allyl pentadecyl ester	M183	-0,011
	cis-Vaccenic acid SUM 264+94	M197	-0,011
	Cyclohexanemethanol, TMS derivative	M200	0,010
	D-(-)-Fructofuranose, pentakis(trimethylsilyl) ether (isomer 2) SUM 217+292	M202	-0,0099
	D-(-)-Ribofuranose, tetrakis(trimethylsilyl) ether (isomer 1) SUM 204+333	M205	0,011
	D-Fructose, 5TMS derivative SUM 306	M213	0,0096
	Diethylene glycol, 2TMS derivative SUM 73+117	M216	0,014
	Diethylene glycol, isobutyl ether, trimethylsilyl ether	M217	0,0089
	Doconexent, TMS derivative SUM 103+129+299+108+319+331+106	M223	0,010
	Eicosapentaenoic Acid, TMS derivative SUM 291+374+217+129+305	M230	-0,0096
	Erucic acid SUM 292+181	M231	-0,012
	Ethanamine, 2TMS derivative	M235	0,0092
	Glucose, 5TMS derivative	M246	0,010
	Glycerol monostearate, 2TMS derivative SUM 331+402	M248	-0,0093
	Heptanoic acid, 4-octyl ester	M257	0,0092
	Hexadecanoic acid, hexadecyl ester	M263	-0,0096
	Hexanoic acid, 2-ethyl-, oxybis(2,1-ethanediyloxy-2,1-ethanediyl) ester	M269	0,012
	Isonicotinic Acid, TBDMS derivative	M274	0,012
	L-Leucine, TMS derivative	M288	-0,014

	L-Serine, 2TMS derivative SUM 116+149	M290	0,013
	N-Acetyl-dl-alanine methylamide	M307	0,0089
	Oleic Acid SUM 284+264+222	M320	-0,0090
	Palmitelaidic acid, TMS derivative SUM 311	M327	0,011
	p-Dicyclohexylbenzene	M333	-0,013
	Pentadecanoic acid, TMS derivative SUM 299	M335	0,013
	phenoxyethanol, TMS derivative	M340	0,0094
	Triacontane, 1-iodo- SUM 113+69	M366	0,014
	Triethylene glycol, 2TMS derivative	M372	0,0093
	Tyrosine, 2TMS derivative	M377	0,010
Proteomics	40S ribosomal protein	E6ZEX1	-0,011
	Inter-alpha-trypsin inhibitor heavy chain H3	E6ZFW4	0,0098
	Glutaredoxin-1	E6ZG15	-0,014
	Small nuclear ribonucleoprotein E	E6ZG90	0,010
	40S ribosomal protein S23	E6ZGE0	-0,012
	Fructose-bisphosphate aldolase	E6ZH34	0,0088
	Ribosomal protein L19	E6ZHE6	0,015
	TANK-binding kinase 1-binding protein	E6ZI15	0,010
	Cystatin-B	E6ZIU0	0,011
	40S ribosomal protein SA	K7DWA9	-0,017
	Calreticulin	M9QRZ6	-0,010
	Na-K-2Cl cotransporter	Q0P6K3	0,0090
	60S ribosomal protein L23	Q9YHX0	-0,0095

Table S2.6: Gills 24h

Source	Compound Name (Metabolites)	Label	Coefficient
Metabolomics	1-Tetradecanol, TMS derivative	M51	-0,083
	2-Monolinolenoylglycerol 1,3-bis(trimethylsilyl) ether SUM 103	M82	-0,082
	Calealactone E	M181	0,073
	Glycerol, 3TMS derivative SUM 71+205	M250	-0,080
	Oxazole, 2-(3Z,6Z,9Z,12Z,15Z,18Z)-3,6,9,12,15,18-heneicosahexaen-1-yl-4,5-dihydro-	M325	-0,077
	p-Dicyclohexylbenzene	M333	-0,077
	Syringaldehyde, TMS derivative	M361	-0,070
Proteomics	Type II keratin E3	E6ZFH2	0,087

Single source models

Table S2.7: Muscle 3h Lipidomics

Compound Name	Label	Coefficient
Cer 38:1;4O|Cer 14:0;3O/24:1;(2OH)	L22	0,037
Cer 39:2;2O|Cer 21:0;2O/18:2	L26	0,038
Cer 45:6;2O|Cer 21:1;2O/24:5	L37	0,036
DG28:2	L45	0,032
DG29:3	L47	0,035
DG32:3	L56	0,035
DG 38:2|DG 19:1_19:1	L90	0,038
FA22:2;4O	L133	-0,036
FA30:7	L144	-0,037
FA36:7	L153	0,038
NAE22:3	L232	0,037
NAE24:4	L235	0,036
TG38:3|TG9:0_9:0_20:3	L602	0,035
TG46:2|TG12:0_16:0_18:2	L622	0,034
TG54:4|TG16:0_18:1_20:3	L763	-0,028
TG57:10|TG17:0_20:5_20:5	L816	-0,030
TG58:0|TG16:0_17:0_25:0	L830	0,031
TG62:1|TG16:0_28:0_18:1	L897	0,031

Table S2.8: Muscle 24h Lipidomics

Compound Name	Label	Coefficient
Cer41:0;4O|Cer21:0;3O/20:0;(2OH)	L30	0,029
DG36:0	L71	0,032
NAE20:1	L230	-0,024
PC40:2|PC16:1_24:1	L327	0,020
PCO-32:1	L371	0,025
SM 34:0;2O|SM 21:0;2O/13:0	L561	-0,028
SM 34:0;3O	L562	-0,029
SM36:1;2O|SM18:1;2O/18:0	L565	0,022
ST24:2;O4	L598	0,023
TG40:0|TG10:0_14:0_16:0	L603	-0,027
TG42:1|TG12:0_14:0_16:1	L609	-0,023
TG44:3|TG14:0_14:0_16:3	L616	-0,027

TG46:4 l TG14:0_16:0_16:4	L628	-0,030
TG50:1 l TG16:0_18:0_16:1	L670	0,020
TG50:7 l TG14:0_16:2_20:5	L684	-0,027
TG51:0 l TG16:0_17:0_18:0	L691	0,025
TG52:0 l TG16:0_18:0_18:0	L707	0,029
TG52:11 l TG16:2_16:4_20:5	L712	-0,027
TG53:0 l TG14:0_15:0_24:0	L733	0,026
TG55:1 l TG15:0_24:0_16:1	L775	0,030
TG56:13 l TG16:3_18:4_22:6	L800	-0,029
TG57:6 l TG17:0_18:1_22:5	L826	-0,026
TG58:12 l TG18:2_18:4_22:6	L840	-0,028
TG58:13 l TG16:2_20:5_22:6	L841	-0,028
TG59:0 l TG16:0_17:0_26:0	L859	0,028
TG61:11 l TG17:0_22:5_22:6	L893	-0,027
TG62:0 l TG16:0_22:0_24:0	L896	0,022

Table S2.9: Skin 3h Lipidomics

Compound Name	Label	Coefficient
CAR16:0	L9	0,035
CAR19:3	L13	-0,032
DG34:7	L67	0,038
DG37:1	L77	0,038
DG42:11 l DG20:5_22:6	L101	-0,035
PC28:1	L174	-0,034
PG47:5	L330	-0,034
SM39:2;2O l SM24:2;2O/15:0	L354	-0,036
TG59:12 l TG17:1_20:5_22:6	L556	-0,034
FA34:9	L639	0,037
FA36:7	L643	0,035
FA42:5	L653	0,035
FA44:5	L658	0,036
PC34:2;3O l PC16:0_18:2;3O	L723	-0,034
PCO-34:2 l PCO-16:0_18:2	L798	-0,035
PCO-36:2 l PCO-18:0_18:2	L806	-0,032
PCO-36:3 l PCO-18:1_18:2	L808	-0,035
PCO-40:6 l PCO-18:0_22:6	L826	-0,037

Table S2.10: Skin 24h Lipidomics

Compound Name	Label	Coefficient
CAR19:0	L12	0,047
DG36:1	L71	0,049
DGO-34:1|DGO-18:0_16:1	L106	-0,050
PC37:7	L225	0,052
TG52:0|TG14:0_18:0_20:0	L439	-0,054
TG53:0|TG14:0_15:0_24:0	L461	-0,056
TG53:1|TG16:0_19:0_18:1	L462	0,047
TG54:0|TG16:0_18:0_20:0	L472	-0,054
TG58:12|TG16:1_20:5_22:6	L540	0,055
TG61:1|TG16:0_27:0_18:1	L579	0,050
FA26:7	L629	0,059
PEO-34:2|PEO-18:1_16:1	L868	-0,049

Table S2.11: Muscle 3h Metabolomics

Compound Name	Label	Coefficient
1-Deoxypentitol, 4TMS derivative SUM 307	M35	0,046
1-Methyl-N,N-bis(trimethylsilyl)-4-[(trimethylsilyl)oxy]-1H-imidazol-2-amine	M41	0,046
4,7,10,13,16,19-Docosahexaenoic acid, methyl ester, (all-Z)- SUM 79+106	M123	-0,049
4-Methylcinnamic acid, trimethylsilyl ester	M132	0,048
Benzene, 1,3,5-triethenyl-2,4,6-triethyl-	M213	0,044
Benzoic acid, 3,5-dimethyl-, trimethylsilyl ester	M220	0,053
Caprolactam	M233	0,045
Cis-9-Hexadecenoic acid, heptyl ester	M251	0,051
Desmethylverapamil	M275	0,049
Desmethylverapamil SUM	M276	0,049
Glycidyl palmitate SUM 116	M321	-0,043
Nonanal	M390	-0,056
Octadecanoic acid, ethyl ester	M399	0,049
Urea, 2TMS derivative	M462	0,043

Table S2.12: Muscle 24h Metabolomics

Compound Name	Label	Coefficient
1-(3-Methylbutyl)-2,3,6-trimethylbenzene	M10	0,043
11,14-Eicosadienoic acid, (Z)-, TMS derivative SUM 365	M25	0,041
1-Octene, 3,7-dimethyl-	M49	0,054

1-Propene-1,2,3-tricarboxylic acid, tributyl ester	M52	0,044
2,2,4-Trimethyl-1,3-pentanediol diisobutyrate	M60	0,047
2-Allyl-1,4-dimethoxybenzene	M81	0,047
4,7,10,13,16,19-Docosahexaenoic acid, methyl ester, (all-Z)- SUM 79+106	M123	-0,046
9-Octadecenoic acid (Z)-, 2,3-dihydroxypropyl ester	M162	0,042
Benzene, (1-methylundecyl)- SUM 246+105	M206	-0,048
Benzophenone	M222	-0,041
D-Fructose, 5TMS derivative	M278	0,045
Eicosanoic acid, ethyl ester	M296	0,045
Hexadecanenitrile	M333	-0,051
Nonanoic acid, TMS derivative	M391	0,043
Sulfurous acid, pentyl tridecyl ester	M441	-0,046

Table S2.13: Skin 3h Metabolomics

Compound Name	Label	Coefficient
11-Tricosene	M28	-0,028
1-Dodecanamine, N,N-dimethyl-	M41	0,034
1-Methyl-N,N-bis(trimethylsilyl)-4-[(trimethylsilyl)oxy]-1H-imidazol-2-amine	M46	0,027
2,3,4-Trihydroxybutyric acid tetrakis(trimethylsilyl) deriv., (, (R*,R*)-) SUM 292	M67	0,029
2-Butenedioic acid, (E)-, 2TMS derivative	M86	0,031
2-Heptanamine, N-(phenylmethylene)-	M89	0,032
6H-Dibenzo[b,d]-pyran	M131	-0,027
7,9-Di-tert-butyl-1-oxaspiro(4,5)deca-6,9-diene-2,8-dione	M132	-0,031
7-Methoxy-3-(2-methoxyphenyl)-2-methylchromen-4-one	M134	-0,025
9-Tricosene, (Z)-	M156	-0,024
Benzaldehyde, 2,4-dimethyl- SUM 63+134	M168	-0,027
Benzoic Acid, TMS derivative	M188	-0,026
D-(-)-Tagatofuranose, pentakis(trimethylsilyl) ether (isomer 2) SUM 217	M216	0,027
D-Arabinopyranose, 4TMS derivative (isomer 2)	M218	0,026
D-Glucose, 5TMS derivative	M221	0,024
Glycol salicylate, TBDMS derivative	M263	0,031
Heptanoic acid, TMS derivative SUM 187+60	M269	0,027
N,N-Diethyl(trimethylsilyl)carbamate	M307	0,021
Nonanoic acid, TMS derivative	M316	0,030
Octanoic acid, TMS derivative SUM 201+116	M322	0,026

Pregna-1,4,6-triene-3,20-dione, 17-(acetyloxy)-6,16-dimethyl-, (16α)-	M343	-0,029
Sorbic acid, TBDMS derivative	M357	0,024
Triacetin	M367	0,030
Z-5-Nonadecene	M381	-0,022

Table S2.14: Skin 24h Metabolomics

Compound Name	Label	Coefficient
1,3-Propanediol, TMS derivative	M15	0,017
10-Heptadecenoic acid, (Z)-, TMS derivative SUM 325+327	M23	0,020
11-Octadecenoic acid, (E)-, TMS derivative	M27	0,021
1-Dodecanamine, N,N-dimethyl-	M41	-0,017
2-(Decanoyloxy)propane-1,3-diyl dioctanoate	M62	0,018
2,5-Cyclohexadiene-1,4-dione, 2,6-bis(1,1-dimethylethyl)-	M73	-0,022
2,5-Dihydroxyacetophenone, 2TBDMS derivative	M74	-0,019
2,5-Di-tert-butyl-4-((trimethylsilyl)oxy)phenol	M79	0,020
2H-Pyran-2-one, tetrahydro-6-undecyl-	M90	0,021
2-Oleoylglycerol, 2TMS derivative	M96	0,017
7-Methyl-Z,Z-8,10-hexadecadien-1-ol acetate	M135	0,020
9,12-Octadecadien-1-ol, (Z,Z)-	M138	0,018
9-Hexadecenoic acid	M145	0,018
Arachidic acid, TMS derivative	M165	0,024
Benzaldehyde, 2,5-dimethoxy- SUM 166	M169	0,020
Benzyl alcohol, TMS derivative SUM 165+180	M191	0,020
Bicyclo[2.2.2]oct-5-en-2-yldimethylamine	M192	0,023
Cholest-3,5-diene	M200	0,018
Decanoic acid, TMS derivative	M219	0,018
Diphenyl ether	M227	0,019
Docosanoic acid, ethyl ester	M230	-0,018
Dodecanamide	M232	0,023
E,E,Z-1,3,12-Nonadecatriene-5,14-diol	M239	0,020
Ethyl iso-allocholate	M250	0,019
Gallacetophenone-4'-methylether, bis(trimethylsilyl) ether	M253	-0,022
Glycidyl (Z)-9-Heptadecenoate	M260	-0,020
Glycidyl palmitoleate SUM 116	M262	-0,020
Hexadecanoic acid, methyl ester	M272	0,022
Lignoceric acid, TMS derivative	M291	-0,017

Monolaurin, 2TMS derivative	M304	0,024
Niacin, TMS derivative	M313	0,021
Palmitic Acid, TMS derivative SUM 286+72+311+313+145+129+327+185+287	M331	-0,019
Undecanoic acid, TMS derivative	M378	0,018
Urea, 2TMS derivative	M379	0,019

Table S2.15: Gills 3h Metabolomics

Compound Name	Label	Coefficient
1,3-Dihydroxyacetone dimer, 4TMS derivative	M9	0,013
1-Monopalmitin, 2TMS derivative	M43	-0,014
1-Tetradecanol, TMS derivative	M51	-0,011
2,3,3-Trimethyl-5-amino-3H-indole	M56	0,010
2,3-Dihydroxypropyl icosanoate, 2TMS derivative	M58	-0,011
2,4,7,9-Tetramethyl-5-decyne-4,7-diol, 2TMS derivative	M59	0,012
2,5-Dimethoxybenzhydrazide	M64	-0,015
2,6-Dihydroxyacetophenone, 2TMS derivative	M70	0,014
2,6-Di-tert-butyl-4-hydroxy-4-methylcyclohexa-2,5-dien-1-one	M71	-0,011
2'-Hydroxy-4'-methoxyacetophenone, TMS derivative	M79	-0,010
2-Monolinolenoylglycerol 1,3-bis(trimethylsilyl) ether SUM 103	M82	-0,014
3-(2-Hydroxyethyl)imidazole-2-thione	M89	0,011
3,4-Dimethylbenzoic acid, TMS derivative	M90	0,010
3,5-di-tert-Butyl-4-hydroxyacetophenone	M91	0,012
3-Methylcyclopentanol, trimethylsilyl ether	M98	0,012
4-tert-Butylphenol, TMS derivative	M110	0,011
5-hydroxy-7-methoxyflavanone, tert.-butyldimethylsilyl ether	M114	-0,010
9(E),11(E)-Conjugated linoleic acid SUM 280	M118	-0,012
9-Hexadecenoic acid, (Z)-, TBDMS derivative	M122	0,016
9-Octadecenenitrile, (Z)-	M129	-0,014
9-Tetradecenal, (Z)-	M134	0,015
9-Tetradecenoic acid, (E)-, TMS derivative	M135	0,012
Acetophenone	M137	0,016
Anthranilic acid, 2TMS derivative	M142	0,013
Arabinofuranose, 1,2,3,5-tetrakis-O-(trimethylsilyl)-	M145	-0,013
Arachidic acid, TMS derivative	M147	0,014
Arachidonic acid, TMS derivative SUM 188+286	M148	-0,012

Benzaldehyde, 2,5-dimethyl-	M157	-0,011
Benzene, (1-ethyldecyl)-	M159	-0,010
Benzene, (1-ethylnonyl)-	M160	-0,014
Benzene, (1-propylnonyl)-	M167	-0,010
Benzene, 1,3-bis(1,1-dimethylethyl)-	M169	0,012
Bis(2-ethylhexyl) methylenesuccinate	M177	0,010
Calealactone E	M181	-0,011
Carbonic acid, allyl pentadecyl ester	M183	-0,014
cis-Vaccenic acid SUM 264+94	M197	-0,013
Cyclohexanemethanol, TMS derivative	M200	0,012
D-(-)-Ribofuranose, tetrakis(trimethylsilyl) ether (isomer 1) SUM 204+333	M205	0,013
D-Fructose, 5TMS derivative SUM 306	M213	0,012
Diethylene glycol, 2TMS derivative SUM 73+117	M216	0,017
Diethylene glycol, isobutyl ether, trimethylsilyl ether	M217	0,011
Di-trimethylsilyl peroxide	M220	-0,010
Doconexent, TMS derivative SUM 103+129+299+108+319+331+106	M223	0,012
Eicosapentaenoic Acid, TMS derivative SUM 291+374+217+129+305	M230	-0,012
Erucic acid SUM 292+181	M231	-0,015
Ethanamine, 2TMS derivative	M235	0,011
Glucose, 5TMS derivative	M246	0,012
Glutaric acid, di(2-ethylhexyl) ester	M247	0,010
Glycerol monostearate, 2TMS derivative SUM 331+402	M248	-0,011
Heptacosan-9-ol	M254	0,011
Heptanoic acid, 4-octyl ester	M257	0,011
Hexadecanoic acid, hexadecyl ester	M263	-0,012
Hexanoic acid	M268	0,010
Hexanoic acid, 2-ethyl-, oxybis(2,1-ethanediyloxy-2,1-ethanediyl) ester	M269	0,015
Isoimperatorin SUM 91+225	M273	0,010
Isonicotinic Acid, TBDMS derivative	M274	0,015
L-Leucine, TMS derivative	M288	-0,016
L-Serine, 2TMS derivative SUM 116+149	M290	0,016
Methylsuccinic acid, 2TMS derivative	M302	0,010
N-Acetyl-dl-alanine methylamide	M307	0,011
Oleic Acid SUM 284+264+222	M320	-0,011
Palmitelaidic acid, TMS derivative SUM 311	M327	0,013
p-Dicyclohexylbenzene	M333	-0,016

Pentadecanoic acid, TMS derivative SUM 299	M335	0,016
Silanamine, N,N'-methanetetraylbis[1,1,1-trimethyl-	M352	0,010
Triacontane, 1-iodo- SUM 113+69	M366	0,017
Triethylene glycol, 2TMS derivative	M372	0,011
Tripropylene glycol monomethyl ether, TMS derivative	M375	-0,010
Tyrosine, 2TMS derivative	M377	0,013

Table S2.16: Gills 24h Metabolomics

Compound Name	Label	Coefficient
1-Tetradecanol, TMS derivative	M51	-0,056
2,5-Dimethoxybenzonitrile	M65	-0,052
2-Monolinolenoylglycerol 1,3-bis(trimethylsilyl) ether SUM 103	M82	-0,055
5-Nonanol, TMS derivative	M115	0,045
Arachidonoyl dimethylaminoethanol	M149	-0,053
Benzene, (1-methyldodecyl)-	M163	0,044
Calealactone E	M181	0,049
Carbonic acid, heptadecyl isobutyl ester	M184	-0,044
D-(-)-Fructofuranose, pentakis(trimethylsilyl) ether (isomer 1) SUM 217	M201	-0,044
D-(-)-Fructopyranose, 5TMS derivative (isomer 2) SUM 204	M203	-0,044
L-Lactic acid	M287	0,045
Oxazole, 2-(3Z,6Z,9Z,12Z,15Z,18Z)-3,6,9,12,15,18-heneicosahexaen-1-yl-4,5-dihydro-	M325	-0,052
p-Dicyclohexylbenzene	M333	-0,052
Syringaldehyde, TMS derivative	M361	-0,048

Table S2.17: Eye 3h Metabolomics

Compound Name	Label	Coefficient
1,3-Dihydroxyacetone dimer, 4TMS derivative	M9	0,013
1-Monopalmitin, 2TMS derivative	M43	-0,014
1-Tetradecanol, TMS derivative	M51	-0,011
2,3,3-Trimethyl-5-amino-3H-indole	M56	0,010
2,3-Dihydroxypropyl icosanoate, 2TMS derivative	M58	-0,011
2,4,7,9-Tetramethyl-5-decyne-4,7-diol, 2TMS derivative	M59	0,012

2,5-Dimethoxybenzhydrazide	M64	-0,015
2,6-Dihydroxyacetophenone, 2TMS derivative	M70	0,014
2,6-Di-tert-butyl-4-hydroxy-4-methylcyclohexa-2,5-dien-1-one	M71	-0,011
2'-Hydroxy-4'-methoxyacetophenone, TMS derivative	M79	-0,010
2-Monolinolenoylglycerol 1,3-bis(trimethylsilyl) ether SUM 103	M82	-0,014
3-(2-Hydroxyethyl)imidazole-2-thione	M89	0,011
3,4-Dimethylbenzoic acid, TMS derivative	M90	0,010
3,5-di-tert-Butyl-4-hydroxyacetophenone	M91	0,012
3-Methylcyclopentanol, trimethylsilyl ether	M98	0,012
4-tert-Butylphenol, TMS derivative	M110	0,011
5-hydroxy-7-methoxyflavanone, tert.-butyldimethylsilyl ether	M114	-0,010
9(E),11(E)-Conjugated linoleic acid SUM 280	M118	-0,012
9-Hexadecenoic acid, (Z)-, TBDMS derivative	M122	0,016
9-Octadecenenitrile, (Z)-	M129	-0,014
9-Tetradecenal, (Z)-	M134	0,015
9-Tetradecenoic acid, (E)-, TMS derivative	M135	0,012
Acetophenone	M137	0,016
Anthranilic acid, 2TMS derivative	M142	0,013
Arabinofuranose, 1,2,3,5-tetrakis-O-(trimethylsilyl)-	M145	-0,013
Arachidic acid, TMS derivative	M147	0,014
Arachidonic acid, TMS derivative SUM 188+286	M148	-0,012
Benzaldehyde, 2,5-dimethyl-	M157	-0,011
Benzene, (1-ethyldecyl)-	M159	-0,010
Benzene, (1-ethylnonyl)-	M160	-0,014
Benzene, (1-propylnonyl)-	M167	-0,010
Benzene, 1,3-bis(1,1-dimethylethyl)-	M169	0,012
Bis(2-ethylhexyl) methylenesuccinate	M177	0,010
Calealactone E	M181	-0,011
Carbonic acid, allyl pentadecyl ester	M183	-0,014
cis-Vaccenic acid SUM 264+94	M197	-0,013
Cyclohexanemethanol, TMS derivative	M200	0,012
D-(-)-Ribofuranose, tetrakis(trimethylsilyl) ether (isomer 1) SUM 204+333	M205	0,013
D-Fructose, 5TMS derivative SUM 306	M213	0,012
Diethylene glycol, 2TMS derivative SUM 73+117	M216	0,017
Diethylene glycol, isobutyl ether, trimethylsilyl ether	M217	0,011

Di-trimethylsilyl peroxide	M220	-0,010
Doconexent, TMS derivative SUM 103+129+299+108+319+331+106	M223	0,012
Eicosapentaenoic Acid, TMS derivative SUM 291+374+217+129+305	M230	-0,012
Erucic acid SUM 292+181	M231	-0,015
Ethanamine, 2TMS derivative	M235	0,011
Glucose, 5TMS derivative	M246	0,012
Glutaric acid, di(2-ethylhexyl) ester	M247	0,010
Glycerol monostearate, 2TMS derivative SUM 331+402	M248	-0,011
Heptacosan-9-ol	M254	0,011
Heptanoic acid, 4-octyl ester	M257	0,011
Hexadecanoic acid, hexadecyl ester	M263	-0,012
Hexanoic acid	M268	0,010
Hexanoic acid, 2-ethyl-, oxybis(2,1-ethanediyloxy-2,1-ethanediyl) ester	M269	0,015
Isoimperatorin SUM 91+225	M273	0,010
Isonicotinic Acid, TBDMS derivative	M274	0,015
L-Leucine, TMS derivative	M288	-0,016
L-Serine, 2TMS derivative SUM 116+149	M290	0,016
Methylsuccinic acid, 2TMS derivative	M302	0,010
N-Acetyl-dl-alanine methylamide	M307	0,011
Oleic Acid SUM 284+264+222	M320	-0,011
Palmitelaidic acid, TMS derivative SUM 311	M327	0,013
p-Dicyclohexylbenzene	M333	-0,016
Pentadecanoic acid, TMS derivative SUM 299	M335	0,016
Silanamine, N,N'-methanetetraylbis[1,1,1-trimethyl-	M352	0,010
Triacontane, 1-iodo- SUM 113+69	M366	0,017
Triethylene glycol, 2TMS derivative	M372	0,011
Tripropylene glycol monomethyl ether, TMS derivative	M375	-0,010
Tyrosine, 2TMS derivative	M377	0,013

Table S2.18: Eye 24h Metabolomics

Compound Name	Label	Coefficient
1-Monolinolein, 2TMS derivative	M40	-0,033
1-Tetradecanol, TMS derivative	M51	-0,037
2,5-Dimethoxybenzonitrile	M65	-0,035
2,6-Dihydroxyacetophenone, 2TMS derivative	M70	0,029

2-Monolinolenoylglycerol 1,3-bis(trimethylsilyl) ether SUM 103	M82	-0,037
5-Nonanol, TMS derivative	M115	0,030
Acrylic acid, 2,3-bis[(trimethylsilyl)oxy]-, trimethylsilyl ester	M138	-0,031
Arabinofuranose, 1,2,3,5-tetrakis-O-(trimethylsilyl)-	M145	-0,031
Arachidonoyl dimethylaminoethanol	M149	-0,035
Benzene, (1-methyldodecyl)-	M163	0,029
Calealactone E	M181	0,033
Carbonic acid, heptadecyl isobutyl ester	M184	-0,029
D-(-)-Fructofuranose, pentakis(trimethylsilyl) ether (isomer 1) SUM 217	M201	-0,029
D-(-)-Fructopyranose, 5TMS derivative (isomer 2) SUM 204	M203	-0,029
Glycerol, 3TMS derivative SUM 71+205	M250	-0,036
Hexadecanoic acid, methyl ester	M264	-0,033
L-Lactic acid	M287	0,030
Methyl stearate	M299	-0,030
Oxazole, 2-(3Z,6Z,9Z,12Z,15Z,18Z)-3,6,9,12,15,18-heneicosahexaen-1-yl-4,5-dihydro-	M325	-0,035
p-Dicyclohexylbenzene	M333	-0,035
Syringaldehyde, TMS derivative	M361	-0,032

Table S2.19: Muscle 3h Proteomics

Compound Name	Label	Coefficient
Myosin heavy chain	Q2PMW9	0,12
Glyceraldehyde-3-phosphate dehydrogenase	Q5I1Z5	-0,12
Slow myosin heavy chain 2	E6ZIC8	0,12
Myomesin-1	E6ZJ46	0,097
Annexin	E6ZHH6	-0,099
Proteasome subunit beta	A0A455ZB90	0,12

Table S2.20: Muscle 24h Proteomics

Compound Name	Label	Coefficient
Slow myosin heavy chain 2	E6ZIC7	-0,15
Inositol-1-monophosphatase	E6ZJ93	-0,13
Myosin heavy chain 1	E6ZIC5	0,11
Annexin	E6ZHH6	0,13
Attractin-like protein 1	E6ZHA4	-0,11

40S ribosomal protein S10	E6ZEX1	-0,12

Table S2.21: Skin 3h Proteomics

Compound Name	Label	Coefficient
Type II keratin E3	E6ZFH2	-0,070
HSP-90	Q6TL18	0,060
Histone H2B	G5CUP6	0,072
Lipocalin	E6ZIH5	-0,081
Hemoglobin-beta	G5CUP5	0,073
Proteasome activator complex	E6ZJF1	0,065
40S ribosomal protein S10	E6ZEX1	0,072
Myosin regulatory light chain 2, smooth muscle isoform	E6ZJ48	-0,059
Keratin, type II cytoskeletal 8	E6ZFH1	-0,073
Apolipoprotein B-100	Q9I8I1	-0,098
Complement component c3-2	F8R8R1	-0,083
Protein disulfide-isomerase A4	E6ZII6	0,086

Table S2.22: Skin 24h Proteomics

Compound Name	Label	Coefficient
Myosin light chain 2	E6ZH67	0,17
Tubulin alpha chain	Q7T1F9	-0,17
Cardiac muscle myosin heavy chain 6 alpha	D5JZI9	0,21
BHMT	A0A6G9HCT5	0,12

Table S2.23: Gills 3h Proteomics

Compound Name	Label	Coefficient
40S ribosomal protein	E6ZEX1	-0,057
Inter-alpha-trypsin inhibitor heavy chain H3	E6ZFW4	0,051
Glutaredoxin-1	E6ZG15	-0,071
Small nuclear ribonucleoprotein E	E6ZG90	0,053
40S ribosomal protein S23	E6ZGE0	-0,062
Ribosomal protein L19	E6ZHE6	0,077
TANK-binding kinase 1-binding protein	E6ZI15	0,052
Cystatin-B	E6ZIU0	0,059
40S ribosomal protein SA	K7DWA9	-0,087
Calreticulin	M9QRZ6	-0,053
Na-K-2Cl cotransporter	Q0P6K3	0,047

60S ribosomal protein L23	Q9YHX0	-0,049

Table S2.24: Gills 24h Proteomics

Compound Name	Label	Coefficient
Interleukin 4/13-2	A0A076YF48	0,046
Phosphotransferase	A0A0A0N292	-0,053
Sodium/potassium-transporting ATPase subunit alpha	A0A0H4U807	0,060
Eukaryotic translation initiation factor 4B	E6ZFG9	-0,046
Keratin, type I cytoskeletal 18	E6ZFH0	-0,058
Type II keratin E3	E6ZFH2	0,072
WD repeat-containing protein 1	E6ZG03	-0,046
Ribonucloprotein	E6ZGP5	-0,054
Histone H1.0	E6ZGX2	-0,048
Serine/arginine-rich splicing factor 2	E6ZHT0	-0,046
40S ribosomal protein S20	E6ZI93	0,050
Inositol-1-monophosphatase	E6ZJ93	-0,050
40S ribosomal protein SA	K7DWA9	0,054
60S ribosomal protein L23	Q9YHX0	0,063
