# Supplementary material for: Multi-Omics Approaches for Freshness Estimation and Detection of Illicit Conservation Treatments in Sea Bass (Dicentrarchus Labrax): Data Fusion Applications
Source: Int J Mol Sci. 2024 Jan 26;25(3):1509. doi: 10.3390/ijms25031509 (PMC10855268; doi:10.3390/ijms25031509)
Supplement: Supplementary file 1 [file ijms-25-01509-s001.zip › Supplementary Materials S1.pdf]

Multi-Omics Approaches for Freshness Estimation and Detection of Illicit Conservation Treatments in Sea Bass (*Dicentrarchus Labrax*): Data Fusion Applications

Alessandro Benedetto, Elisa Robotti, Masho Hilawie Belay, Arianna Ghignone, Alessia Fabbris, Eleonora Goggi, Simone Cerruti, Marcello Manfredi, Elettra Barberis, Simone Peletto, Alessandra Arillo, Nunzia Giaccio, Maria Angela Masini, Jessica Brandi, Daniela Cecconi, Emilio Marengo and Paola Brizio

Supplementary Material S1.
Here are reported with more details all the results of analysis made on 16S rRNA sequencing data.
For material and Methods please refer to section "4.3.4 Metagenomics" of the manuscript.

Sample list for metagenomic analysis

sample ID	Tissue	Group	time point	Number of Reads
1	Gills	Treated	t24	325112
2	Eye	Untreated	t24	78444
3	Skin	Treated	t24	499066
4	Skin	Untreated	t0	359096
5	Skin	Treated	t24	567028
6	Eye	Treated	t24	728582
7	Skin	Untreated	t24	163252
8	Control mock sample	Positive Control	/	482584
9	Gills	Untreated	t24	175294
10	Gills	Treated	t0	84200
11	Skin	Treated	t0	395243
12	Eye	Untreated	t0	232086
13	Gills	Untreated	t0	157692
14	Skin	Untreated	t0	<10000
15	Eye	Untreated	t0	310886
16	Gills	Untreated	t0	<10000
17	Eye	Treated	t0	459918
18	Skin	Treated	t0	98822
19	Eye	Treated	t0	2344202
20	Gills	Treated	t24	146310
21	Gills	Treated	t24	114082
22	Eye	Untreated	t24	88978
23	Skin	Untreated	t24	175536
24	Gills	Untreated	t24	158080
25	Control mock sample	Positive Control	/	274506
26	Gills	Treated	t0	158092
27	Skin	Treated	t0	89732
28	Skin	Untreated	t0	50270
29	Gills	Untreated	t0	243996
30	Eye	Untreated	t0	<10000
31	Eye	Treated	t0	50062
32	Skin	Treated	t0	82332
33	Gills	Treated	t0	<10000

34	Gills	Untreated	t0	67272
35	Eye	Untreated	t0	49882
36	Skin	Untreated	t0	130138
37	Gills	Treated	t0	193148
38	Eye	Treated	t0	246606
39	Control mock sample	Positive Control	/	201608
40	Control mock sample	Positive Control	/	58050
41	Eye	Untreated	t24	247488
42	Gills	Untreated	t24	53262
43	Skin	Untreated	t24	916836
44	Eye	Treated	t24	76604
45	Eye	Treated	t0	<10000
46	Skin	Treated	t0	115034
47	Gills	Treated	t0	102370
48	Eye	Untreated	t0	49764
49	Gills	Untreated	t0	189860
50	Skin	Untreated	t0	178474
51	Control mock sample	Positive Control	/	149254
52	Control negative sample	Negative Control	/	<10000
53	Gills	Treated	t24	134350
54	Skin	Treated	t24	<10000
55	Eye	Treated	t24	50378
56	Eye	Untreated	t24	84910
57	Skin	Untreated	t24	<10000
58	Gills	Untreated	t24	99280
59	Eye	Treated	t24	<10000
60	Skin	Treated	t24	138584
61	Eye	Treated	t24	196670
62	Eye	Untreated	t24	<10000
63	Gills	Untreated	t0	1105720
64	Skin	Untreated	t0	<10000
65	Gills	Treated	t24	<10000
66	Gills	Untreated	t24	41010
67	Eye	Untreated	t0	5305538
68	Skin	Treated	t0	91992
70	Skin	Untreated	t24	396890
71	Eye	Treated	t0	<10000
72	Gills	Treated	t0	84112
73	Gills	Treated	t24	167012
74	Eye	Treated	t24	<10000
75	Skin	Treated	t24	<10000
76	Skin	Untreated	t24	51212
77	Eye	Untreated	t24	62224
78	Gills	Untreated	t24	94124

Shifting from class to family level the abundances of the different taxonomic groups were reported as follows.

Supplementary Table S1: Abundances of the most represented taxonomic groups (families) considering all the analyzed sea bass samples.

Family	Combined Abundance	Min	Max	Mean	Median
Pseudoalteromonadaceae	181311	0	146715	2924.37	81.5
Moraxellaceae	74089	0	19384	1194.98	83
Comamonadaceae	73769	0	45743	1189.82	45
Shewanellaceae	65167	0	33069	1051.08	35.5
Flavobacteriaceae	28631	0	6119	461.79	25.5
Vibrionaceae	13857	0	5361	223.5	1
Xanthobacteraceae	4692	0	3118	75.68	1
Propionibacteriaceae	3604	0	1747	58.13	1
Pseudomonadaceae	2463	0	1027	39.72	1
Corynebacteriaceae	2210	0	2108	35.64	0
Moritellaceae	2125	0	1965	34.27	0
Oxalobacteraceae	604	0	412	9.74	0
Rhizobiaceae	575	0	571	9.27	0
Micrococcaceae	328	0	142	5.29	0
Carnobacteriaceae	267	0	117	4.31	0
Psychromonadaceae	201	0	185	3.24	0
Rhodobacteraceae	161	0	48	2.59	0
Burkholderiaceae	136	0	92	2.19	0
Weeksellaceae	110	0	52	1.77	0

Supplementary Figure S1: Graphical representation of family distribution among sampling times (3 hours and 24 hours after starting of experiment), tissues (gills, skin and eye respectively) and treatment applied (Cafodos or untreated controls).

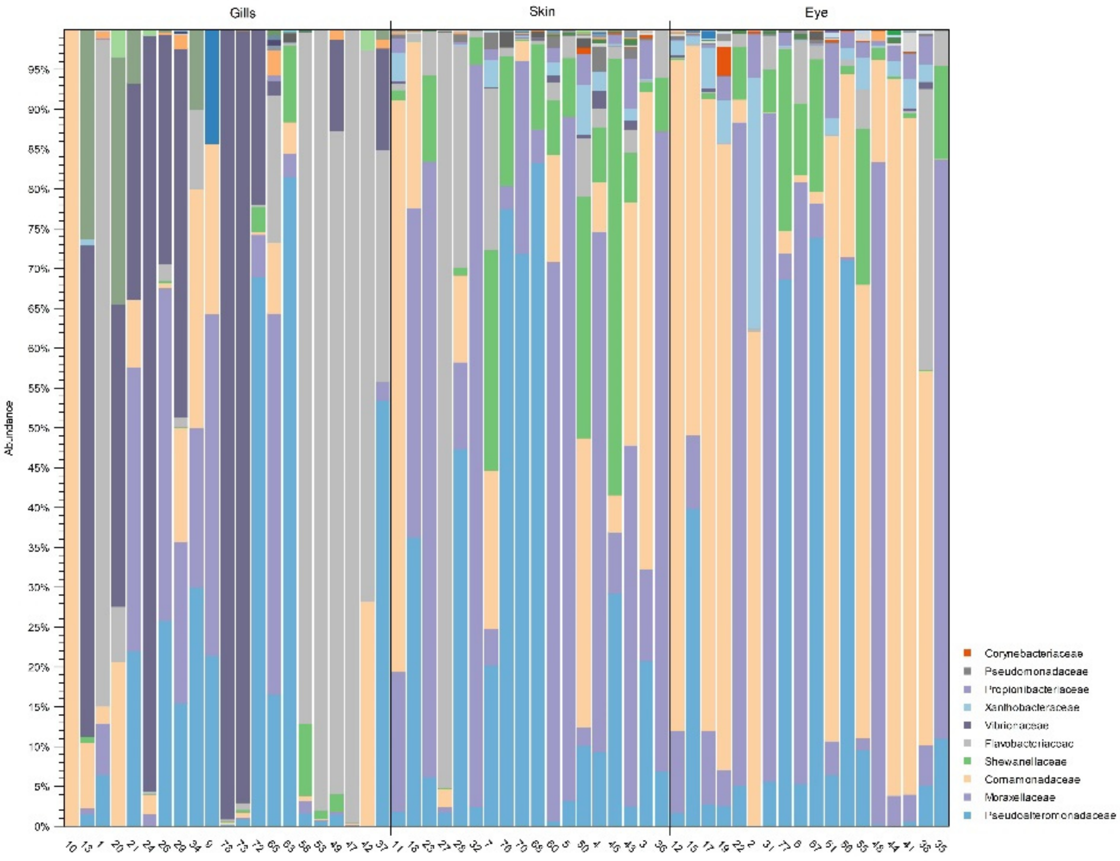
Gills
Skin
Eye
Abundance
Corynebacteriaceae
Pseudomonadaceae
Propionibacteriaceae
Xanthobacteraceae
Vibrionaceae
Flavobacteriaceae
Shewanellaceae
Comamonadaceae
Moraxellaceae
Pseudoalteromonadaceae

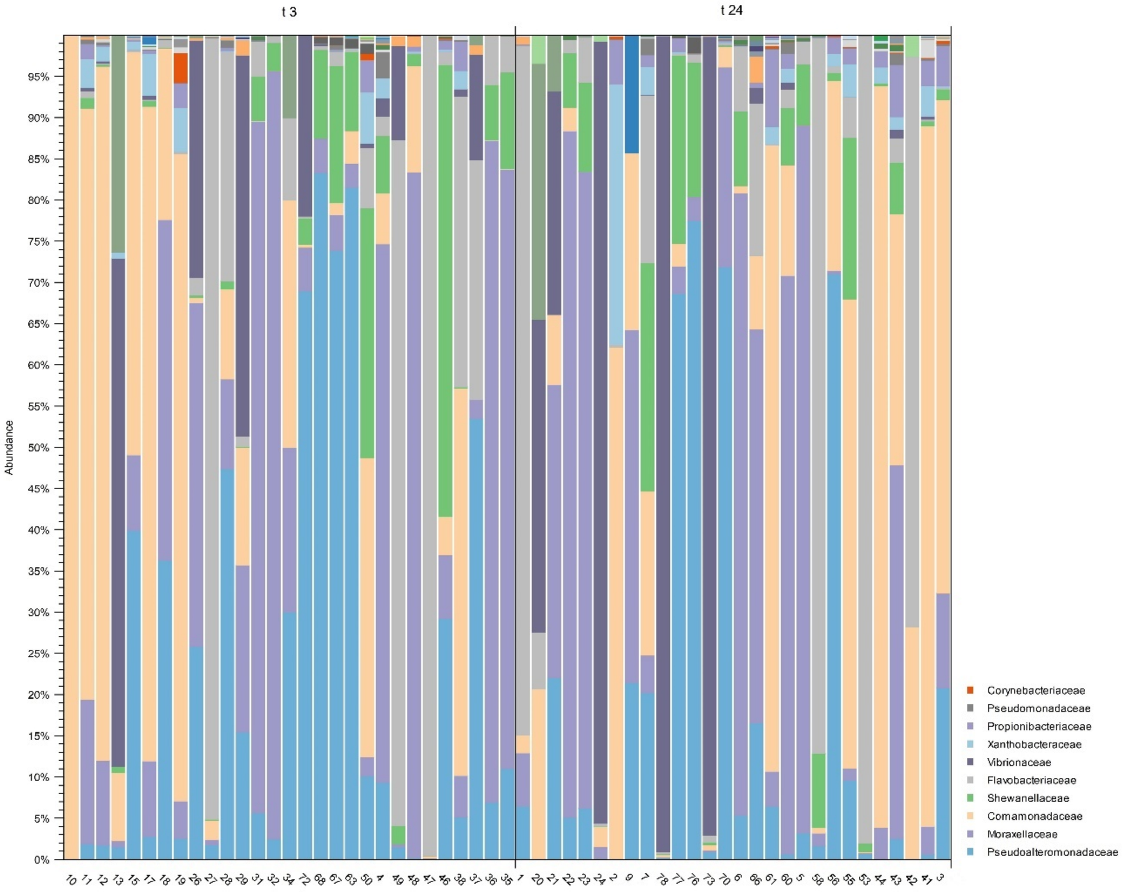
t 3
t 24
Abundance
95%
90%
85%
80%
75%
70%
65%
60%
55%
50%
45%
40%
35%
30%
25%
20%
15%
10%
5%
0%
Corynebacteriaceae
Pseudomonadaceae
Propionibacteriaceae
Xanthobacteraceae
Vibrionaceae
Flavobacteriaceae
Shewanellaceae
Comamonadaceae
Moraxellaceae
Pseudoalteromonadaceae

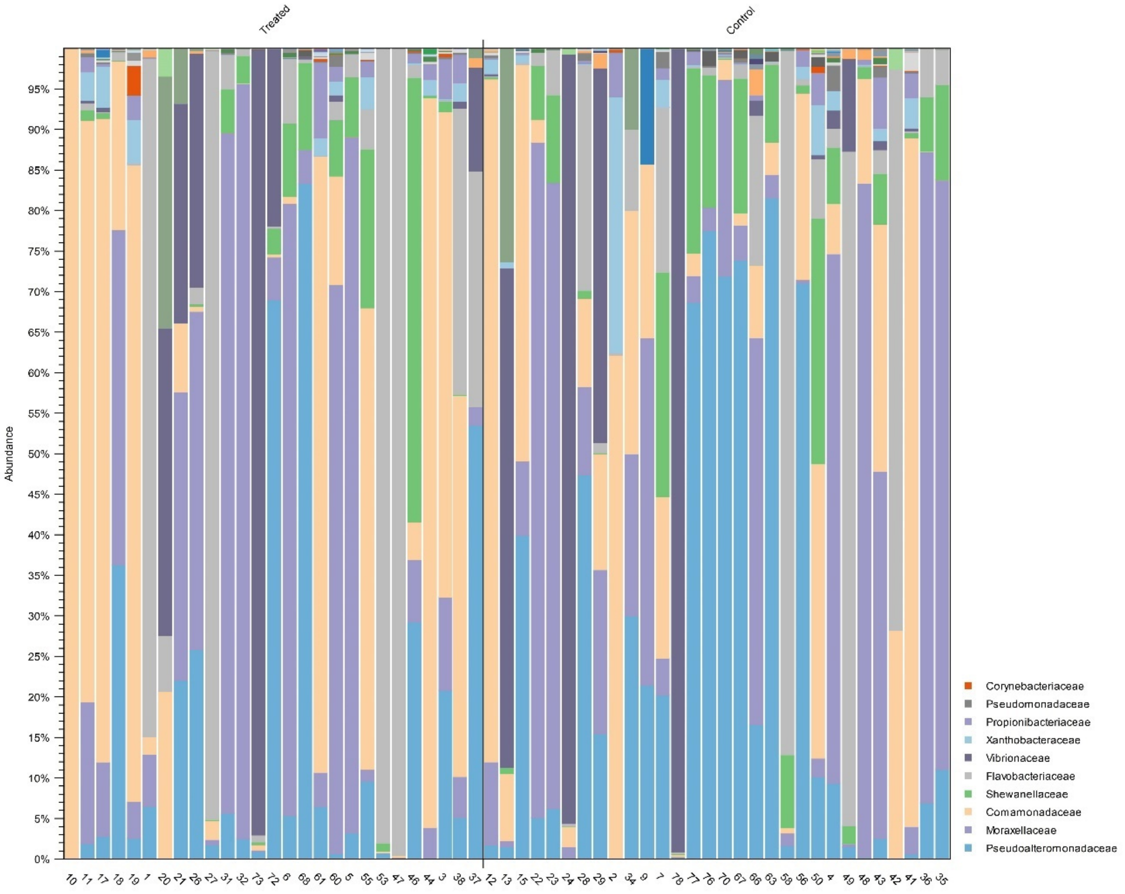
Treated
Control
Abundance
95%
90%
85%
80%
75%
70%
65%
60%
55%
50%
45%
40%
35%
30%
25%
20%
15%
10%
5%
0%
Corynebacteriaceae
Pseudomonadaceae
Propionibacteriaceae
Xanthobacteraceae
Vibrionaceae
Flavobacteriaceae
Shewanellaceae
Comamonadaceae
Moraxellaceae
Pseudoalteromonadaceae

In the following figures and tables are reported all the comparisons made for Differential Abundance Analysis (DAA). For each comparisons is reported on the left an histogram on microbiome composition in the two compared groups, on the right alpha diversity analysis and related Krustal Wallis test). If according to DAA significant difference in comparisons made were recorded, a supplementary table with fold changes and FDR p values is reported.

- *Controls specimens (3 hours stored on ice) VS Cafodos treated specimens (3 hours after exposure)*

- *Gills*

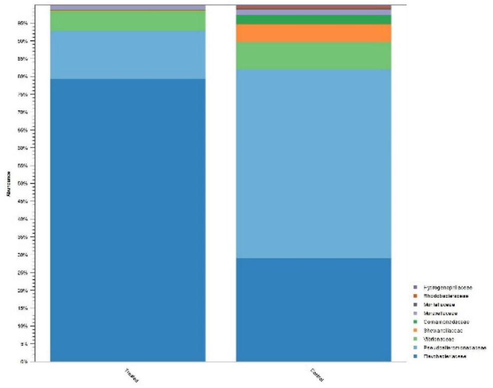

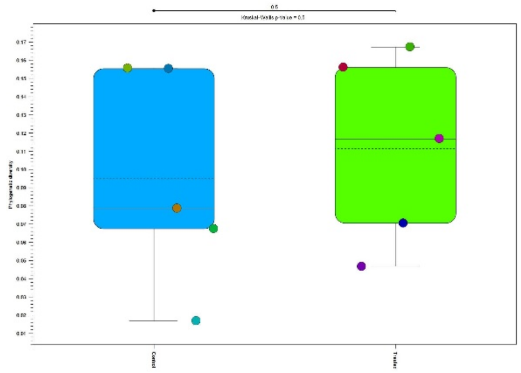

- *Eye*

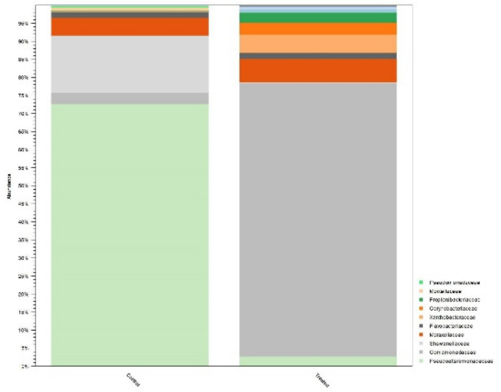

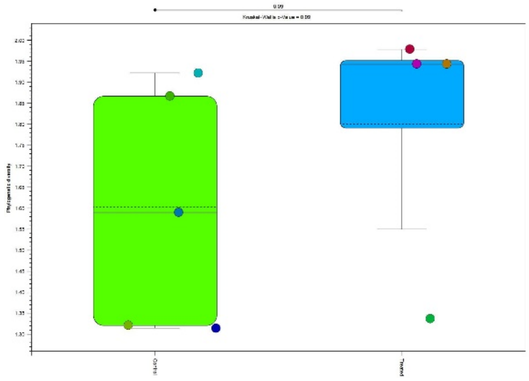

- *Skin*

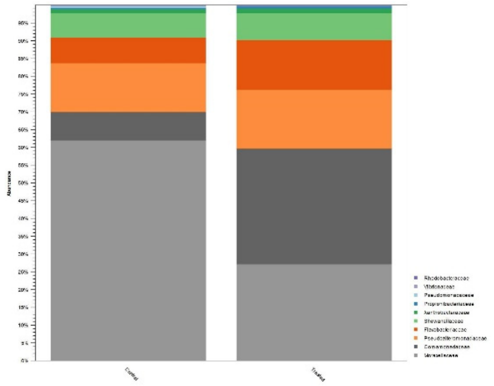

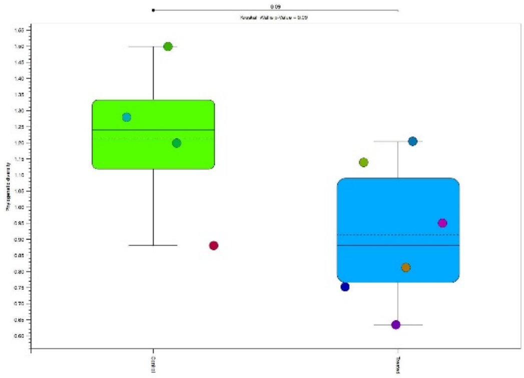

- Controls specimens (24 hours stored on ice) VS Cafodos treated specimens (24 hours after exposure)

- *Gills*

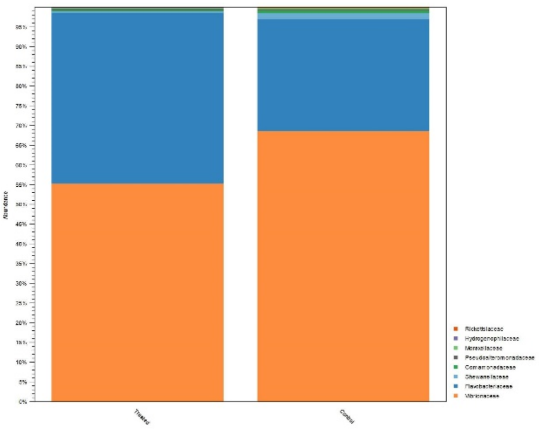

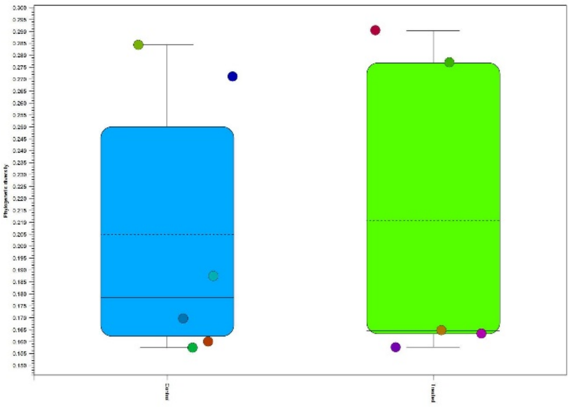

Control VS Treated (24h)	Max group mean	Log_2 fold change	Fold change	P-value	FDR p-value
Hydrogenophilaceae	2.8	-7.89268937	-237.6491399	0.004420086	0.03536069
Flavobacteriaceae	839.8	-0.417666481	-1.335765243	0.704051484	0.911303247
Rickettsiaceae	2	1.620091522	3.073945363	0.455728527	0.877692061
Comamonadaceae	11.16666667	0.736531005	1.666164686	0.547097254	0.877692061
Pseudoalteromonadaceae	7	-1.084595517	-2.120780805	0.510489774	0.877692061
Shewanellaceae	25.33333333	-1.260348653	-2.395536263	0.548557538	0.877692061
Vibrionaceae	1118.333333	0.802455031	1.744066477	0.797390341	0.911303247
Moraxellaceae	5.333333333	0.018012269	1.012563418	0.992635843	0.992635843

- *Eye*

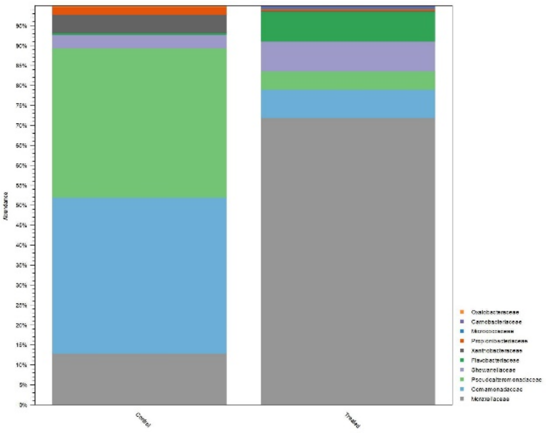

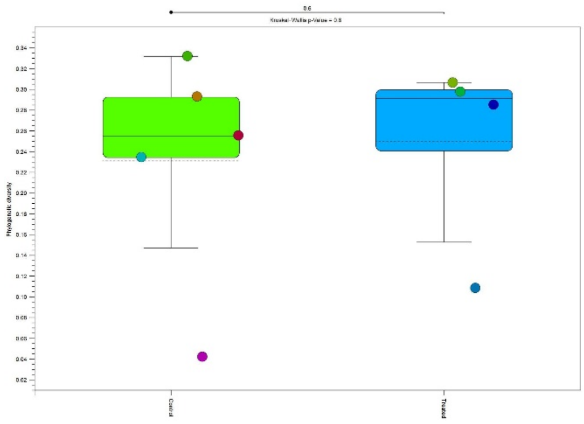

- *Skin*

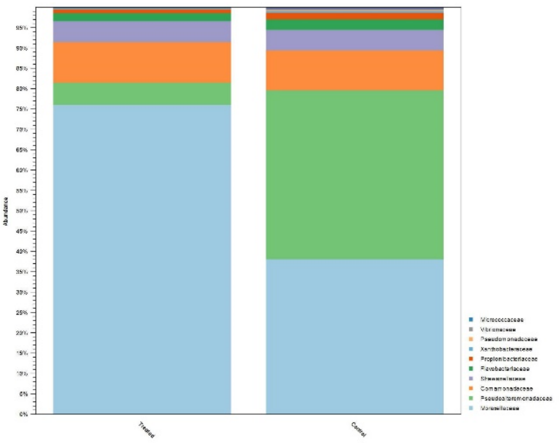

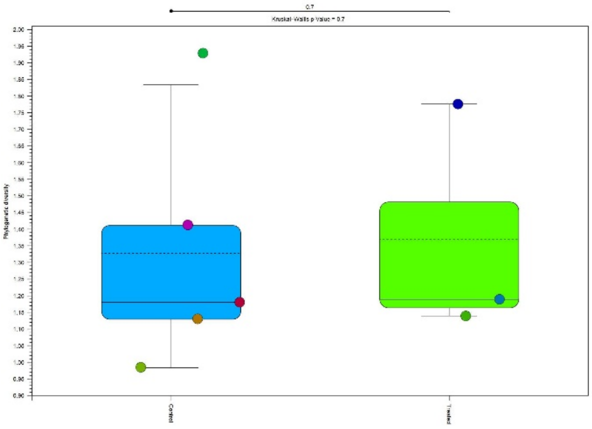

- Controls specimens (3 hours stored on ice) VS Cafodos treated specimens (3 hours after exposure). All specimens from each fish were aggregate (skin+gills+eye)

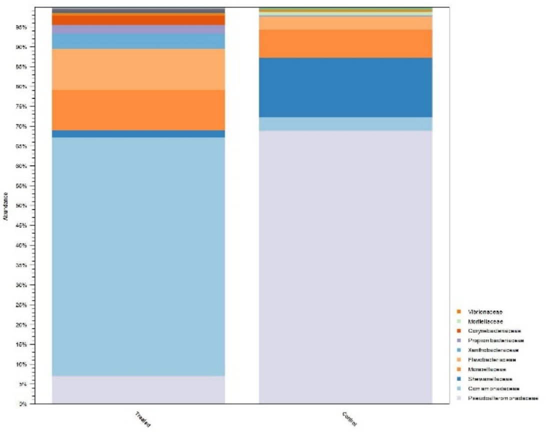

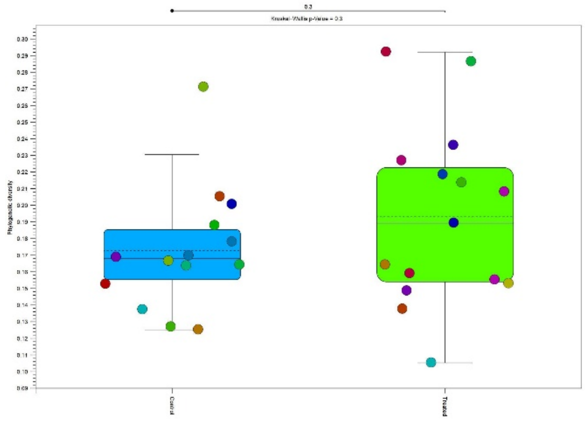

Control VS Treated (3h)	Max group mean	Log2 fold change	Fold change	P-value	FDR p-value
Rhizobiaceae	38.066667	-8.431409	-345.228787	0.000833	0.011323
Flavobacteriaceae	624.000000	-4.416358	-21.352862	0.000871	0.011323
Listeriaceae	1.357143	-5.074122	-33.687054	0.003944	0.034179
Hydrogenophilaceae	2.642857	4.803519	27.925652	0.006260	0.040693
Burkholderiaceae	6.133333	-3.185048	-9.094837	0.009956	0.047598
Sporolactobacillaceae	4.266667	-5.603728	-48.628425	0.010984	0.047598
Rhodobacteraceae	5.428571	2.941826	7.683833	0.016759	0.056142
Exiguobacteraceae	2.666667	-5.063048	-33.429450	0.017275	0.056142
Beijerinckiaceae	1.266667	-4.263308	-19.203638	0.032302	0.093318
Chitinophagaceae	1.000000	-4.024547	-16.274568	0.038424	0.095276
Actinomycetaceae	0.733333	-3.722579	-13.201029	0.047240	0.102353
Corynebacteriaceae	140.600000	-4.052705	-16.595324	0.040309	0.095276
Micrococcaceae	0.714286	0.631453	1.549124	0.562802	0.665130
Propionibacteriaceae	130.066667	-2.418898	-5.347623	0.094849	0.164404
Acetobacteraceae	1.142857	2.164990	4.484633	0.145368	0.222327
Xanthobacteraceae	234.866667	-1.849098	-3.602749	0.192466	0.263375
Comamonadaceae	3631.666667	-1.614465	-3.061981	0.157339	0.227267
Oxalobacteraceae	27.933333	-2.153734	-4.449780	0.104359	0.169583

Aeromonadaceae	1.642857	3.170839	9.005704	0.085502	0.158790
Moritellaceae	148.071429	2.762276	6.784655	0.082115	0.158790
Pseudoalteromonadaceae	11102.428571	0.298766	1.230092	0.675969	0.732300
Psychromonadaceae	13.571429	0.933937	1.910482	0.462719	0.574396
Shewanellaceae	2408.500000	0.334923	1.261310	0.741053	0.741053
Vibrionaceae	64.357143	1.006676	2.009277	0.463935	0.574396
Moraxellaceae	1137.642857	-0.222317	-1.166606	0.722984	0.741053
Pseudomonadaceae	68.071429	0.589323	1.504540	0.606634	0.685760

- Controls specimens (24 hours stored on ice) VS Cafodos treated specimens (24 hours after exposure). All specimens from each fish were aggregate (skin+gills+eye)

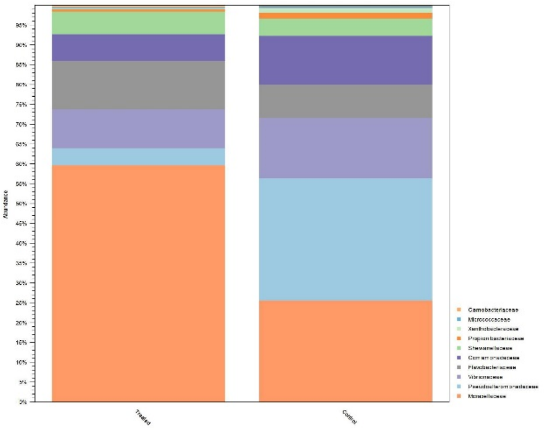

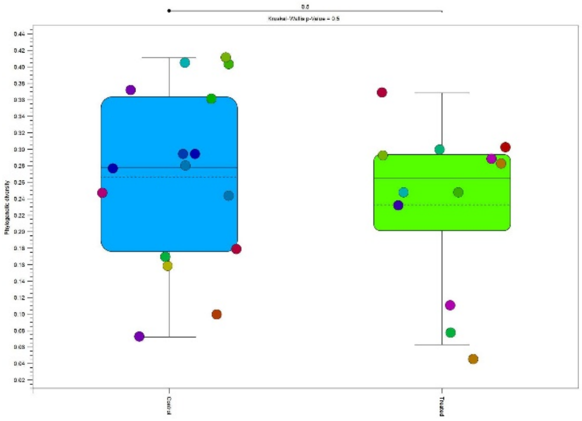

Control VS Treated (24h)	Max group mean	Log_2 fold change	Fold change	P-value	FDR p-value
Hydrogenophilaceae	1.25	-5.827594	-56.791147	0.000211	0.003369
Oxalobacteraceae	1.1875	4.502508	22.666787	0.005762	0.0461
Rickettsiaceae	0.75	4.022073	16.246675	0.012169	0.064901
Flavobacteriaceae	555.666667	-2.9783	-7.880571	0.018659	0.074637
{Unknown Family} Peptostreptococcales-Tissierellales	2	4.023363	16.26121	0.02648	0.084736
Carnobacteriaceae	10.75	-2.224166	-4.672407	0.058551	0.156137
Micrococcaceae	15.25	-0.72608	-1.654139	0.569966	0.839146
Propionibacteriaceae	39.4375	-0.754705	-1.687286	0.584451	0.839146
Weeksellaceae	3.0625	2.240837	4.726713	0.132326	0.264652
Xanthobacteraceae	30.625	2.032886	4.092228	0.11946	0.264652
Comamonadaceae	340.125	0.149266	1.109005	0.896784	0.907903
Pseudoalteromonadaceae	853.875	0.513703	1.42771	0.64498	0.839146
Shewanellaceae	259.75	-0.128076	-1.092835	0.907903	0.907903
Vibrionaceae	447.916667	1.160259	2.234975	0.556153	0.839146
Moraxellaceae	2727.58333	-0.447502	-1.363677	0.6842	0.839146
Pseudomonadaceae	3.75	-0.40763	-1.326505	0.734253	0.839146

- *Controls specimens (3 hours stored on ice) VS Control specimens (24 hours after storage on ice).*

- *Gills*

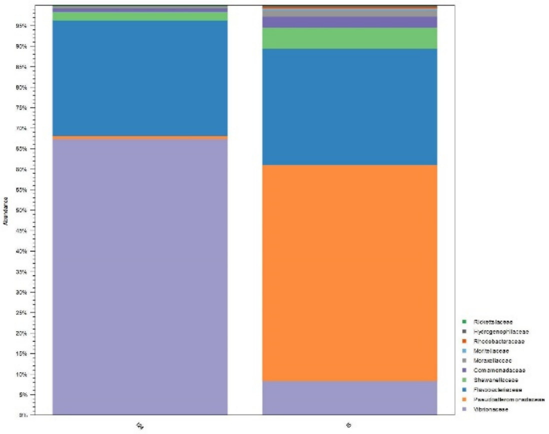

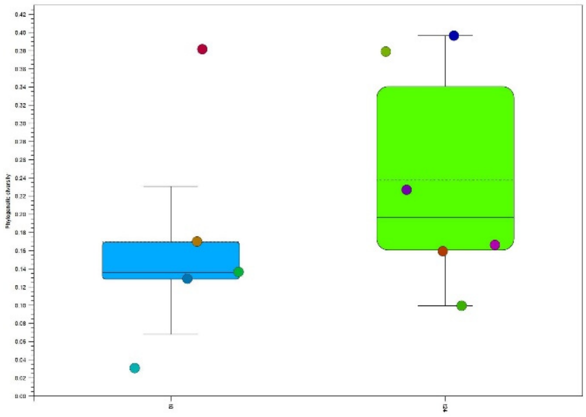

- *Eye*

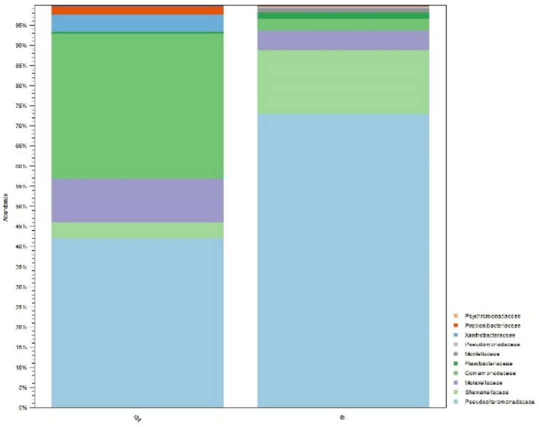

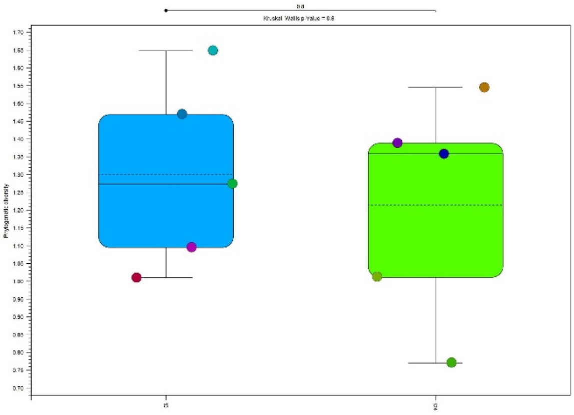

- *Skin*

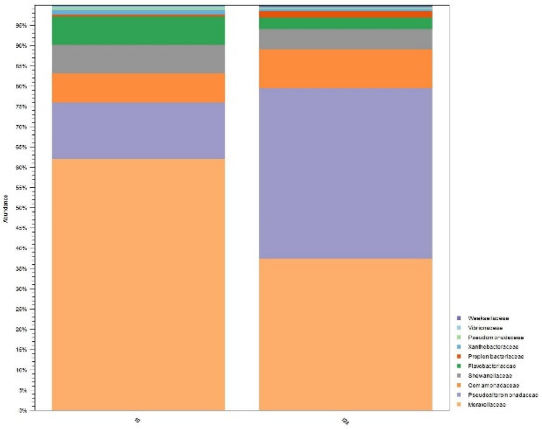

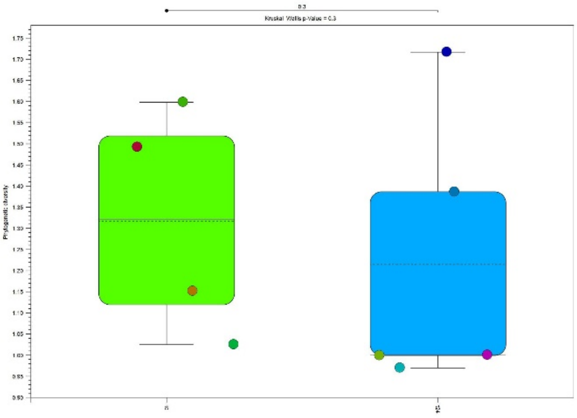

- Cafodos treated specimens (3 hours after exposure) VS Cafodos treated specimens (24 hours after exposure)

- *Gills*

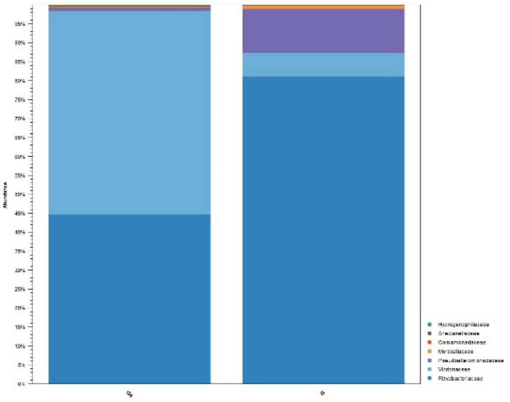

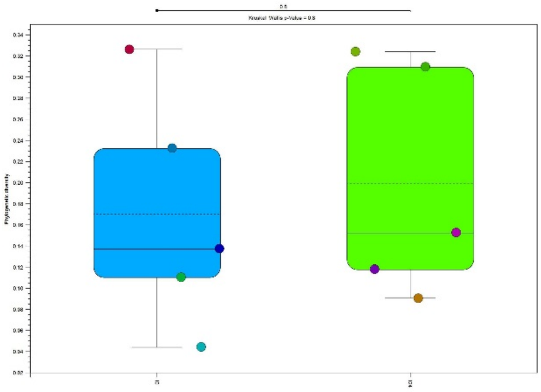

- *Eye*

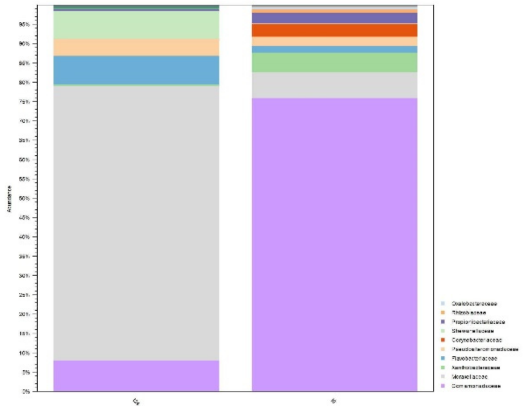

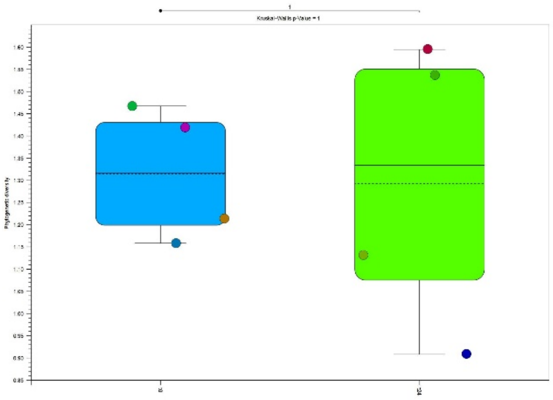

Treated (3h) VS Treated (24h)	Max group mean	Log$_2$ fold change	Fold change	P-value	FDR p-value
Xanthobacteraceae	810.2500	6.2640	76.8511	0.0000	0.0004
Comamonadaceae	12065.7500	6.0533	66.4100	0.0001	0.0013
Oxalobacteraceae	103.0000	5.1912	36.5359	0.0016	0.0092
Burkholderiaceae	21.0000	4.1037	17.1924	0.0017	0.0092
Corynebacteriaceae	526.0000	9.2004	588.2765	0.0041	0.0147
Propionibacteriaceae	437.2500	3.9641	15.6073	0.0042	0.0147
Listeriaceae	2.5000	11.0559	2128.9845	0.0054	0.0161
Rhizobiaceae	142.7500	10.4288	1378.4422	0.0089	0.0234
Vibrionaceae	4.2500	8.2503	304.5008	0.0101	0.0235
Sporolactobacillaceae	16.0000	7.7093	209.2814	0.0291	0.0612
Exiguobacteraceae	10.0000	7.2045	147.4944	0.0363	0.0694
Actinomycetaceae	2.7500	5.9915	63.6236	0.0600	0.0901
Micrococcaceae	27.7500	0.5619	1.4762	0.8789	0.9228
Chitinophagaceae	3.7500	6.2375	75.4552	0.0551	0.0891
Flavobacteriaceae	462.2500	1.1037	2.1491	0.6588	0.7344
Carnobacteriaceae	20.5000	-2.2987	-4.9200	0.3633	0.4768
Beijerinckiaceae	4.7500	6.4554	87.7538	0.0504	0.0882

Pseudoalteromonadaceae	381.7500	2.4748	5.5589	0.1399	0.1959
Shewanellaceae	454.5000	0.0472	1.0333	0.9862	0.9862
Moraxellaceae	4445.2500	1.4483	2.7288	0.4925	0.6084
Pseudomonadaceae	3.0000	1.1804	2.2664	0.6645	0.7344

- *Skin*

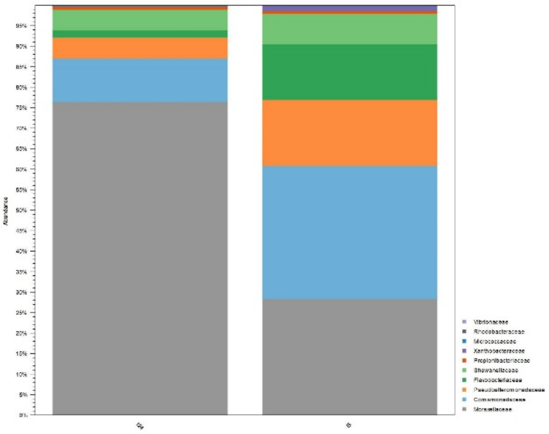

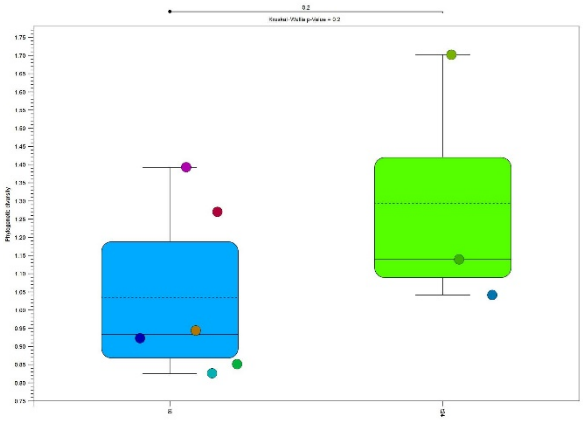

Treated (3h) VS Treated (24h)	Max group mean	Log₂ fold change	Fold change	P-value	FDR p-value
Flavobacteriaceae	350.0000	6.5285	92.3130	0.0039	0.0228
Pseudoalteromonadaceae	416.0000	3.2172	9.3001	0.0042	0.0228
Micrococcaceae	6.6667	-2.2465	-4.7452	0.2057	0.4526
Propionibacteriaceae	40.3333	0.6018	1.5176	0.8063	0.8063
Xanthobacteraceae	31.6667	2.2226	4.6674	0.4315	0.6780
Rhodobacteraceae	2.1667	3.4187	10.6938	0.1347	0.4086
Comamonadaceae	840.1667	2.3375	5.0544	0.2916	0.5345
Shewanellaceae	279.6667	2.8992	7.4600	0.1486	0.4086
Vibrionaceae	1.6667	1.6296	3.0942	0.5064	0.6964
Moraxellaceae	4230.3333	-0.5983	-1.5139	0.6286	0.7586
Pseudomonadaceae	2.6667	-0.7311	-1.6599	0.6897	0.7586
