# Supplementary material for: Multi-Omics Approaches for Freshness Estimation and Detection of Illicit Conservation Treatments in Sea Bass (Dicentrarchus Labrax): Data Fusion Applications
Source: Int J Mol Sci. 2024 Jan 26;25(3):1509. doi: 10.3390/ijms25031509 (PMC10855268; doi:10.3390/ijms25031509)
Supplement: Supplementary file 1 [file ijms-25-01509-s001.zip › Supplementary Materials S2.pdf]

Multi-Omics Approaches for Freshness Estimation and Detection of Illicit Conservation Treatments in Sea Bass (*Dicentrarchus Labrax*): Data Fusion Applications

Alessandro Benedetto, Elisa Robotti, Masho Hilawie Belay, Arianna Ghignone, Alessia Fabbris, Eleonora Goggi, Simone Cerruti, Marcello Manfredi, Elettra Barberis, Simone Peletto, Alessandra Arillo, Nunzia Giaccio, Maria Angela Masini, Jessica Brandi, Daniela Cecconi, Emilio Marengo and Paola Brizio

Supplementary Material S2.

Here are reported the contribution plots of the first two Dimensions calculated for MFA applied to the single sampling sites datasets. Just the first 100 molecules are reported for each graphical representation. In each representation, the variables are on the x-axis, while the contribution is on the y-axis.

MUSCLE

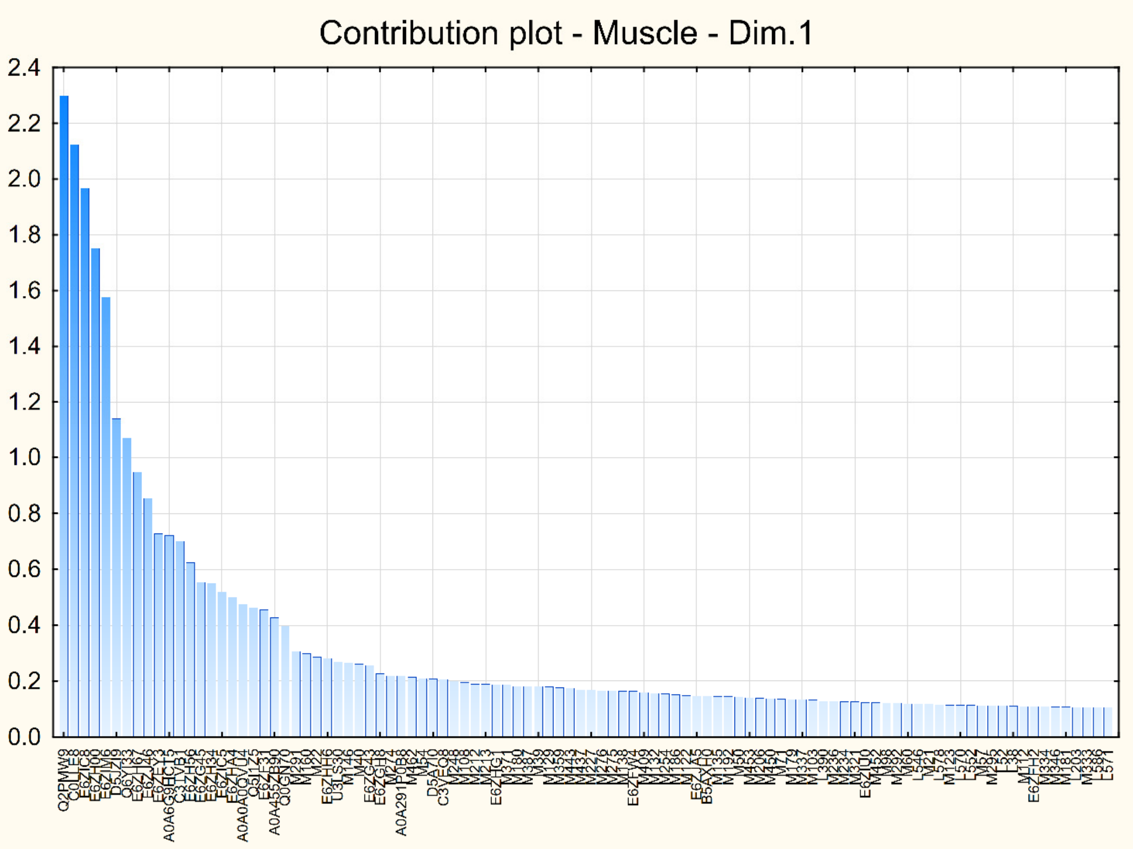

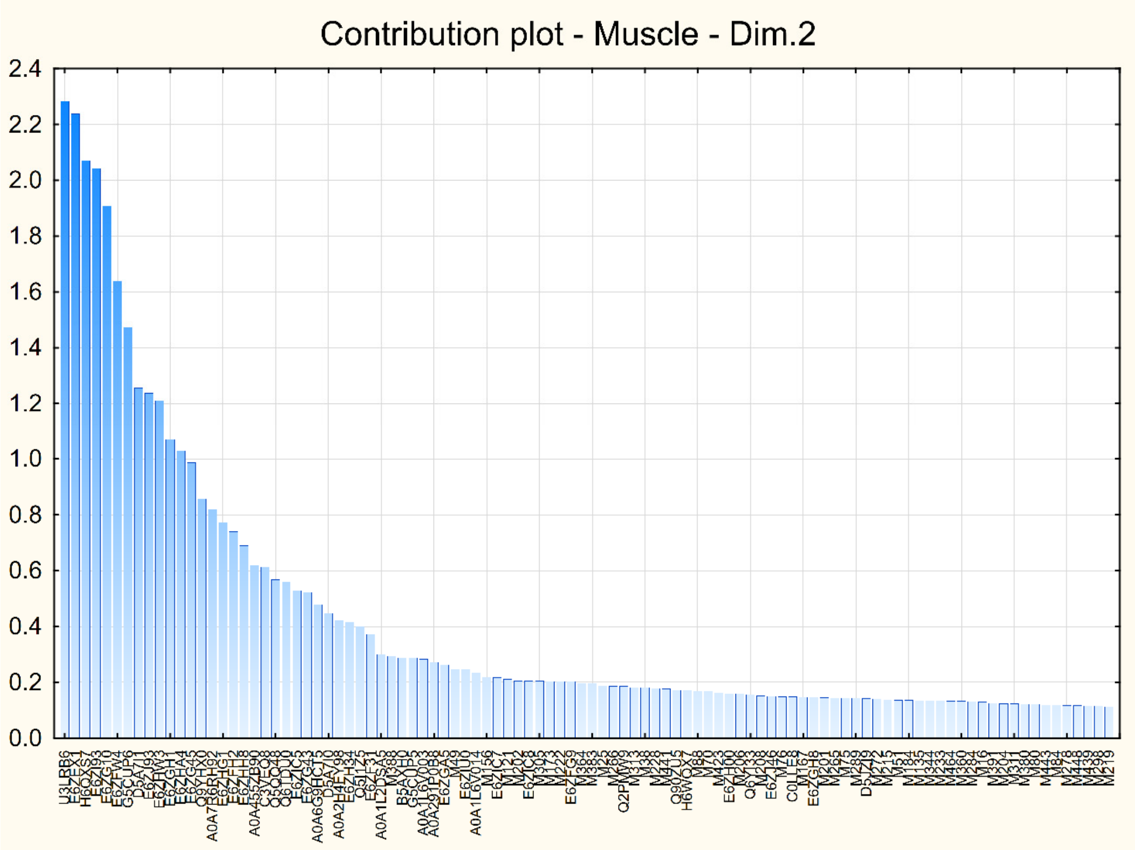
Contribution plot - Muscle - Dim.2

SKIN

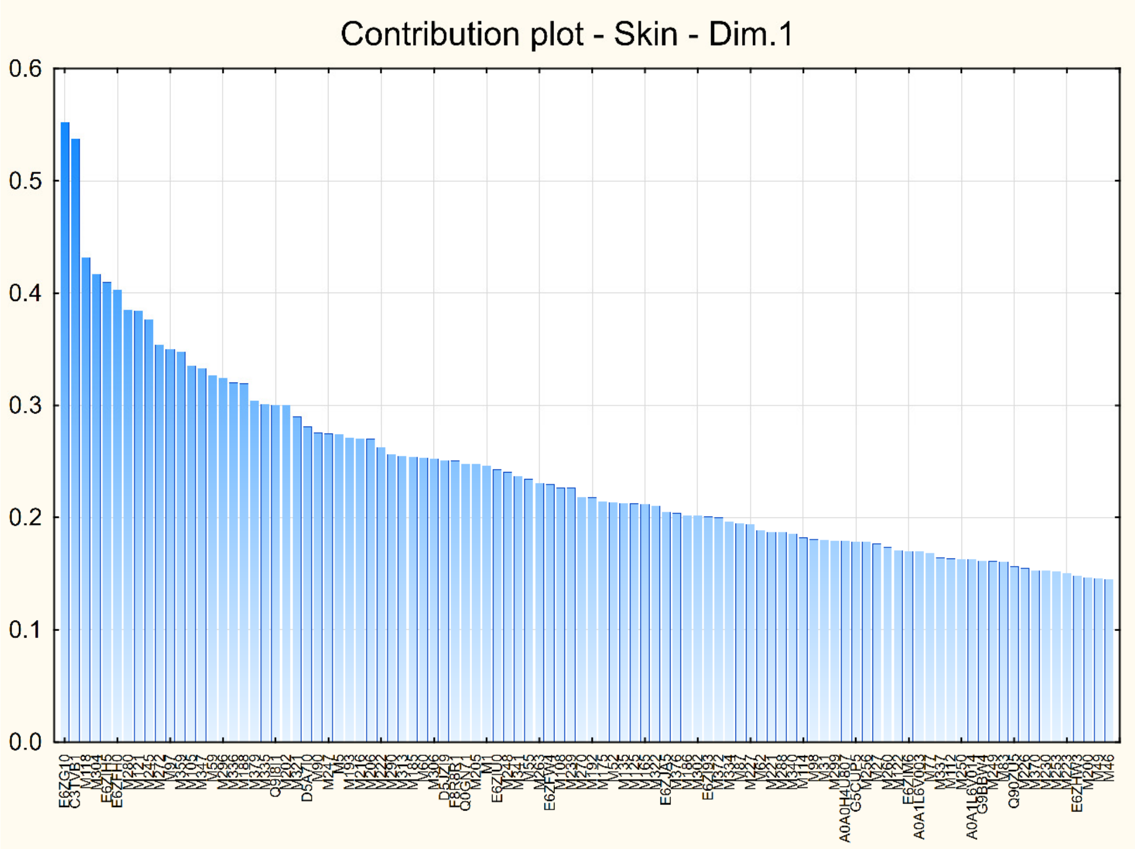
Contribution plot - Skin - Dim.1

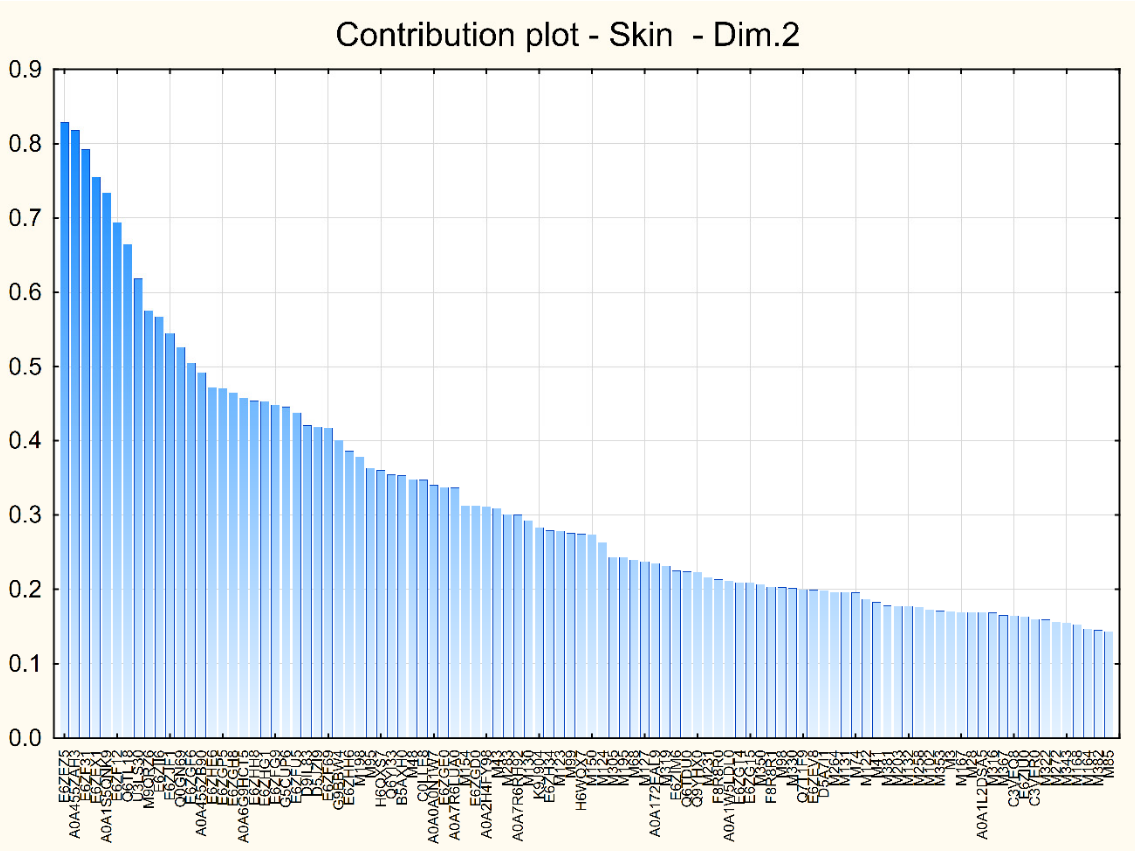

GILLS

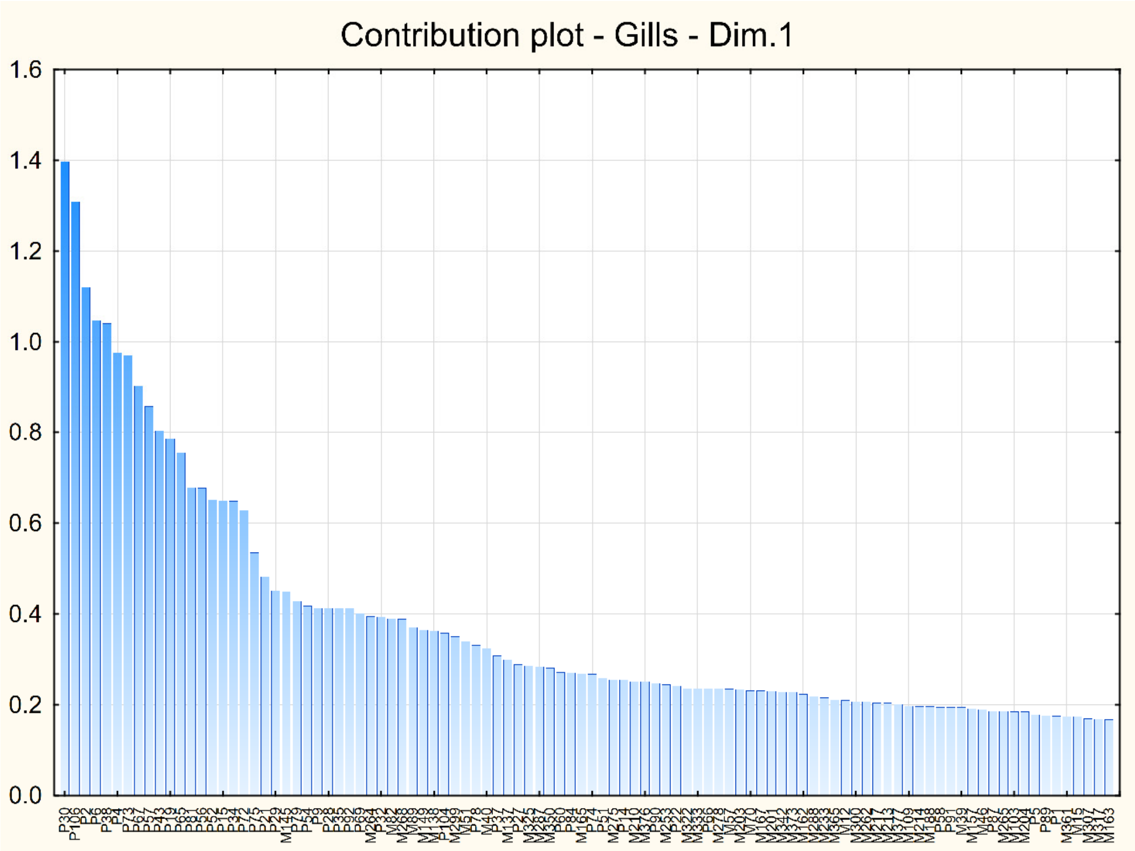

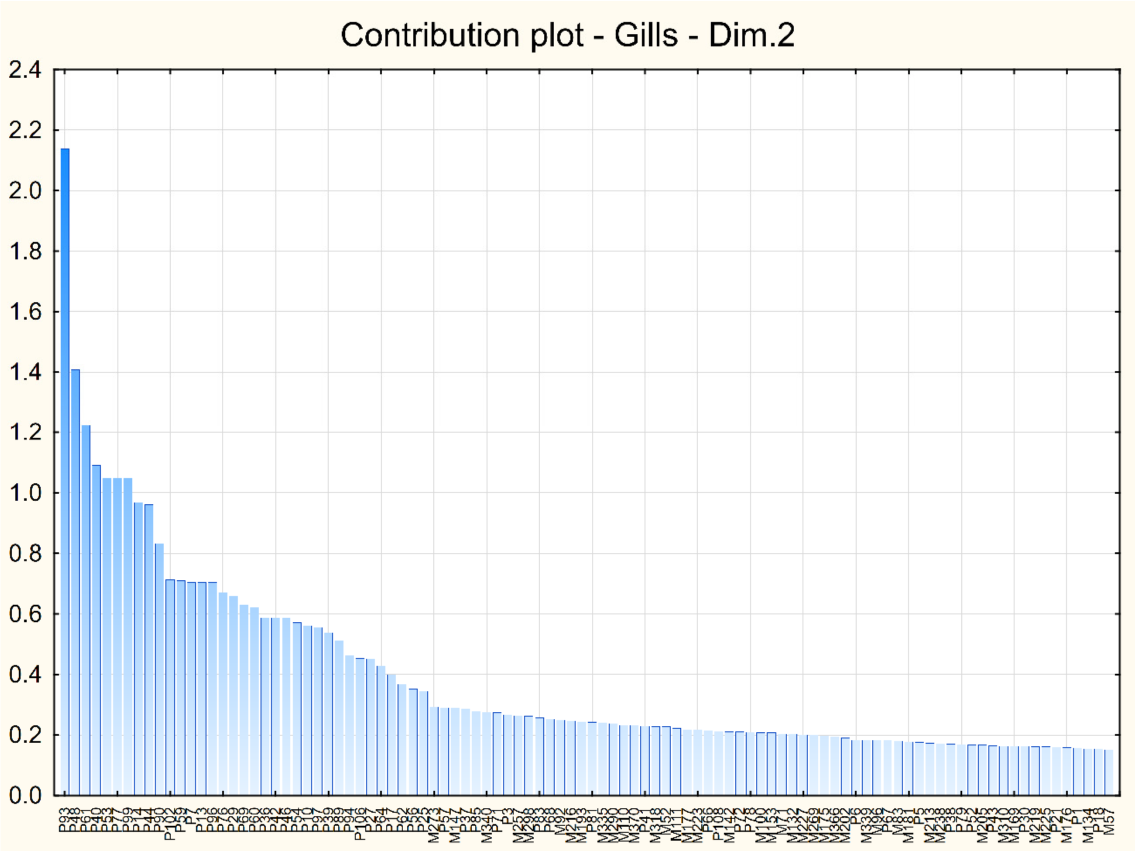
Contribution plot - Gills - Dim.2
2.4
2.2
2.0
1.8
1.6
1.4
1.2
1.0
0.8
0.6
0.4
0.2
0.0

EYE

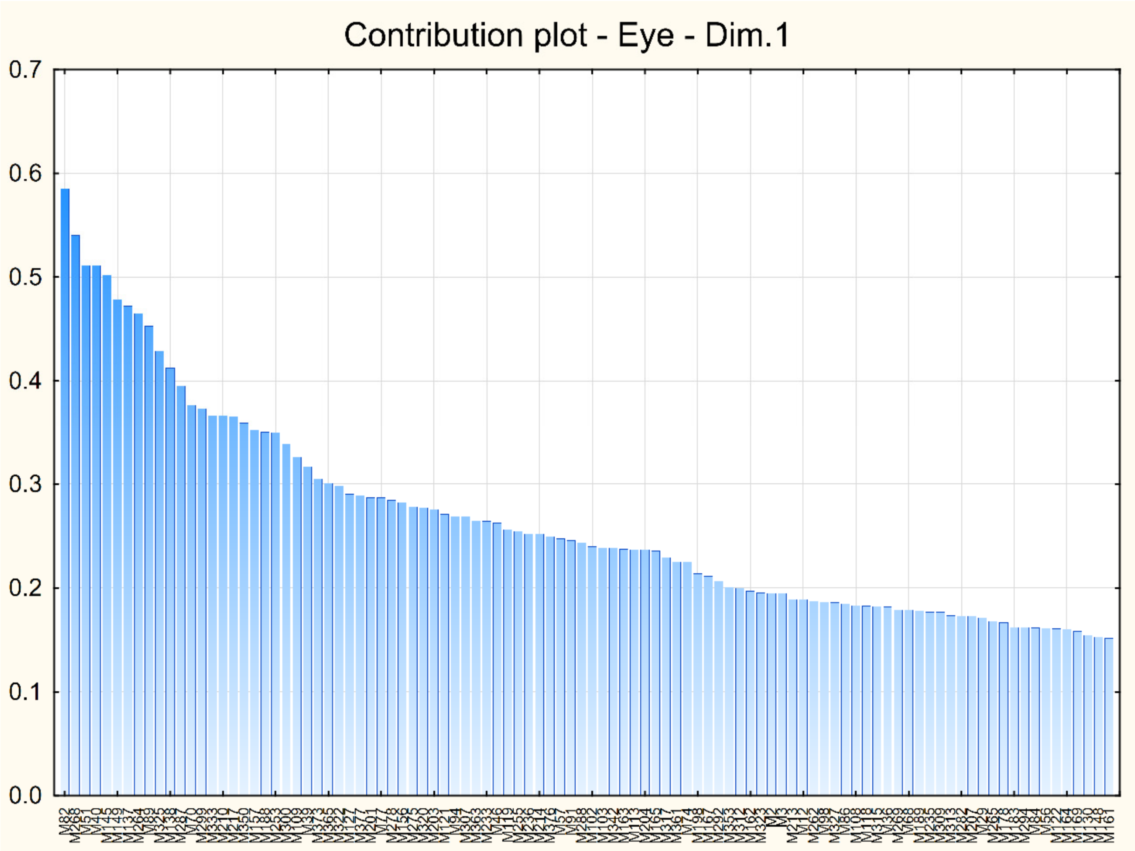
Contribution plot - Eye - Dim.1
0.7
0.6
0.5
0.4
0.3
0.2
0.1
0.0

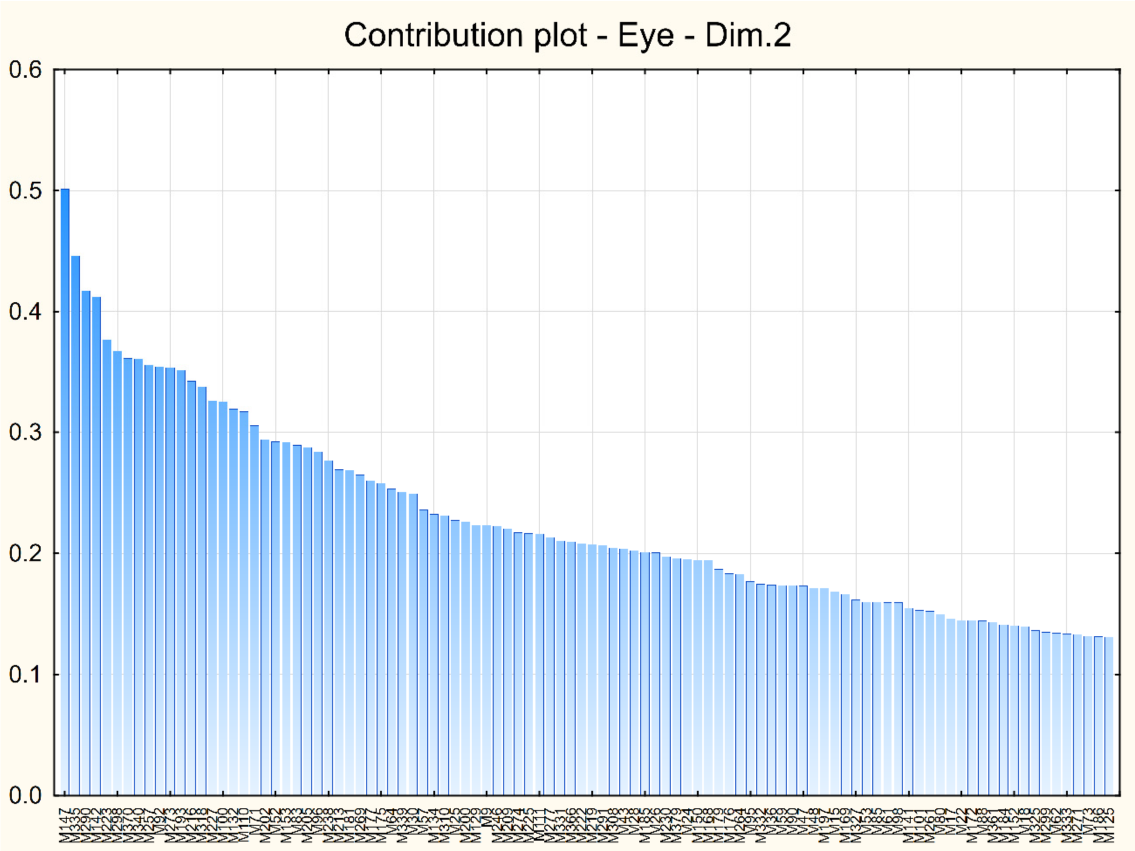
Contribution plot - Eye - Dim.2
0.6
0.5
0.4
0.3
0.2
0.1
0.0
